# Genome-Wide Inference of Ancestral Recombination Graphs

**DOI:** 10.1371/journal.pgen.1004342

**Published:** 2014-05-15

**Authors:** Matthew D. Rasmussen, Melissa J. Hubisz, Ilan Gronau, Adam Siepel

**Affiliations:** 1 Department of Biological Statistics and Computational Biology, Cornell University, Ithaca, New York, United States of America; 2 European Molecular Biology Laboratory, European Bioinformatics Institute, Wellcome Trust Genome Campus, Hinxton, Cambs, United Kingdom; University of California Davis, United States of America

## Abstract

The complex correlation structure of a collection of orthologous DNA sequences is uniquely captured by the “ancestral recombination graph” (ARG), a complete record of coalescence and recombination events in the history of the sample. However, existing methods for ARG inference are computationally intensive, highly approximate, or limited to small numbers of sequences, and, as a consequence, explicit ARG inference is rarely used in applied population genomics. Here, we introduce a new algorithm for ARG inference that is efficient enough to apply to dozens of complete mammalian genomes. The key idea of our approach is to sample an ARG of 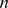 chromosomes conditional on an ARG of 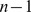 chromosomes, an operation we call “threading.” Using techniques based on hidden Markov models, we can perform this threading operation exactly, up to the assumptions of the sequentially Markov coalescent and a discretization of time. An extension allows for threading of subtrees instead of individual sequences. Repeated application of these threading operations results in highly efficient Markov chain Monte Carlo samplers for ARGs. We have implemented these methods in a computer program called *ARGweaver*. Experiments with simulated data indicate that *ARGweaver* converges rapidly to the posterior distribution over ARGs and is effective in recovering various features of the ARG for dozens of sequences generated under realistic parameters for human populations. In applications of *ARGweaver* to 54 human genome sequences from Complete Genomics, we find clear signatures of natural selection, including regions of unusually ancient ancestry associated with balancing selection and reductions in allele age in sites under directional selection. The patterns we observe near protein-coding genes are consistent with a primary influence from background selection rather than hitchhiking, although we cannot rule out a contribution from recurrent selective sweeps.

## Author Summary

The unusual and complex correlation structure of population samples of genetic sequences presents a fundamental statistical challenge that pervades nearly all areas of population genetics. Historical recombination events produce an intricate network of intertwined genealogies, which impedes demography inference, the detection of natural selection, association mapping, and other applications. It is possible to capture these complex relationships using a representation called the ancestral recombination graph (ARG), which provides a complete description of coalescence and recombination events in the history of the sample. However, previous methods for ARG inference have not been adequately fast and accurate for practical use with large-scale genomic sequence data. In this article, we introduce a new algorithm for ARG inference that has vastly improved scaling properties. Our algorithm is implemented in a computer program called *ARGweaver*, which is fast enough to be applied to sequences megabases in length. With the aid of a large computer cluster, *ARGweaver* can be used to sample full ARGs for entire mammalian genome sequences. We show that *ARGweaver* performs well in simulation experiments and demonstrate that it can be used to provide new insights about both demographic processes and natural selection when applied to real human genome sequence data.

## Introduction

At each genomic position, orthologous DNA sequences drawn from one or more populations are related by a branching structure known as a genealogy [1], [2]. Historical recombination events lead to changes in these genealogies from one genomic position to the next, resulting in a correlation structure that is complex, analytically intractable, and poorly approximated by standard representations of high-dimensional data. Over a period of many decades, these unique features of genetic data have inspired numerous innovative techniques for probabilistic modeling and statistical inference [3]–[9], and, more recently, they have led to a variety of creative approaches that achieve computational tractability by operating on various summaries of the data [10]–[17]. Nevertheless, none of these approaches fully captures the correlation structure of collections of DNA sequences, which inevitably leads to limitations in power, accuracy, and generality in genetic analysis.

**Figure 1:**
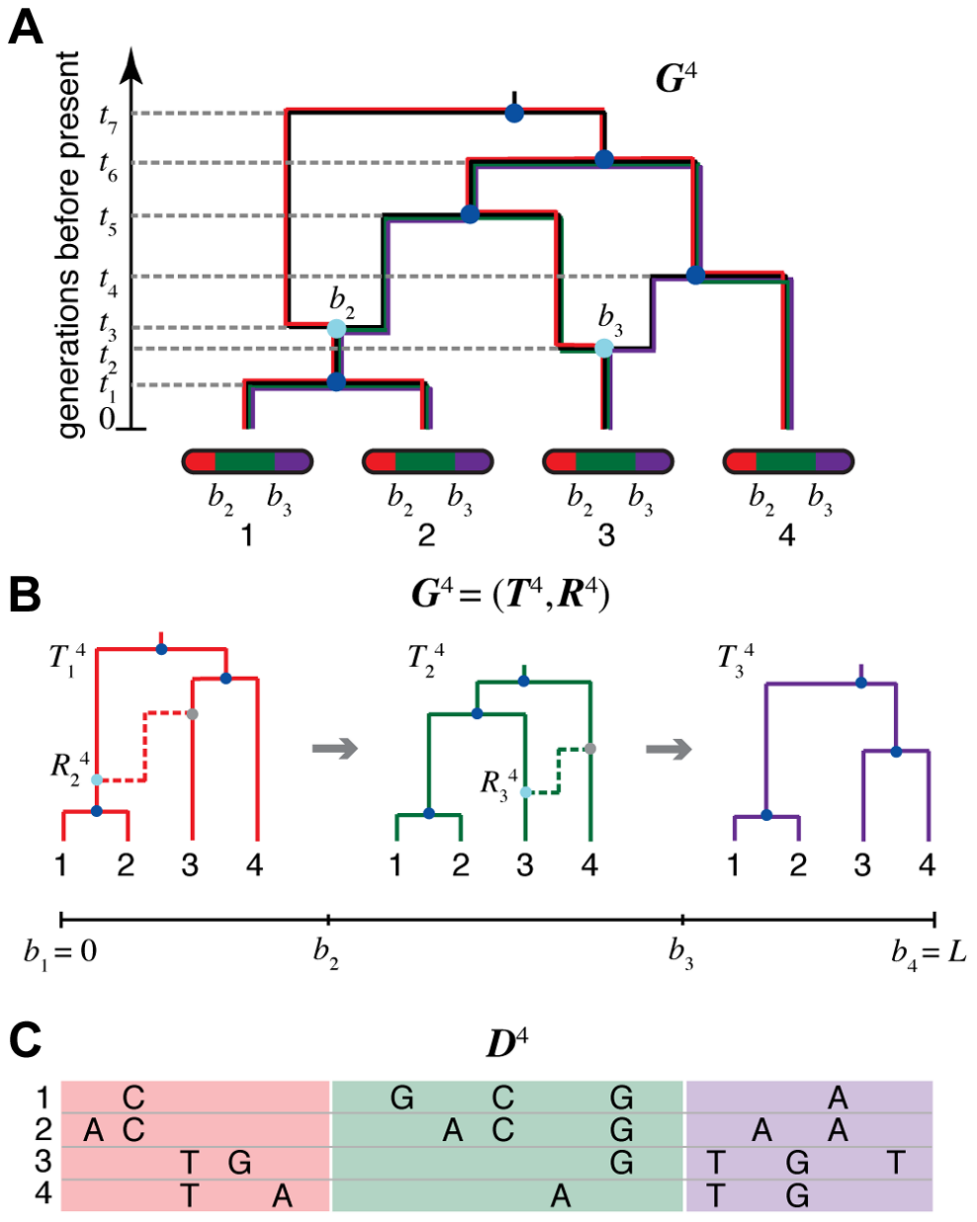
An ancestral recombination graph (ARG) for four sequences. (A) Going backwards in time (from bottom to top), the graph shows how lineages that lead to modern-day chromosomes (bottom) either “coalesce” into common ancestral lineages (dark blue circles), or split into the distinct parental chromosomes that were joined (in forward time) by recombination events (light blue circles). Each coalescence and recombination event is associated with a specific time (dashed lines), and each recombination event is also associated with a specific breakpoint along the chromosomes (here, 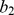 and 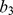). Each non-recombining interval of the sequences (shown in red, green, and purple) corresponds to a “local tree” embedded in the ARG (shown in matching colors). Recombinations cause these trees to change along the length of the sequences, making the correlation structure of the data set highly complex. The ARG for four sequences is denoted 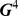 in our notation. (B) Representation of 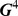 in terms of a sequence of local trees 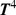 and recombination events 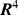. A local tree 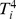 is shown for each nonrecombining segment in colors matching those in (A). Each tree, 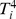, can be viewed as being constructed from the previous tree, 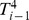, by placing a recombination event along the branches of 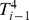 (light blue circles), breaking the branch at this location, and then allowing the broken lineage to re-coalesce to the rest of the tree (dashed lines in matching colors; new coalescence points are shown in gray). Together, the local trees and recombinations provide a complete description of the ARG. The Sequentially Markov Coalescent (SMC) approximate the full coalescent-with-recombination by assuming that 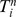 is statistically independent of all previous trees given 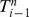. (C) An alignment of four sequences, 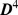, corresponding to the linearized ARG shown in (B). For simplicity, only the derived alleles at polymorphic sites are shown. The sequences are assumed to be generated by a process that samples an ancestral sequences from a suitable background distribution, then allows each nonrecombining segment of this sequence to mutate stochastically along the branches of the corresponding local tree. Notice that the correlation structure of the sequences is fully determined by the local trees; that is, 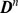 is conditionally independent of the recombinations 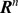 given the local trees 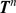.

In principle, the correlation structure of a collection of colinear orthologous sequences can be fully described by a network known as an *ancestral recombination graph* (ARG) [18]–[20]. An ARG provides a record of all coalescence and recombination events since the divergence of the sequences under study and specifies a complete genealogy at each genomic position (Figure 1A). In many senses, the ARG is the ideal data structure for population genomic analysis. Indeed, if an accurate ARG could be obtained, many problems of interest today—such as the estimation of recombination rates or ancestral effective population sizes—would become trivial, while many other problems—such as the estimation of population divergence times, rates of gene flow between populations, or the detection of selective sweeps—would be greatly simplified. Various data representations in wide use today, including the site frequency spectrum, principle components, haplotype maps, and identity by descent spectra, can be thought of as low-dimensional summaries of the ARG and are strictly less informative.

An extension of the widely used coalescent framework [1], [2], [9] that includes recombination [21] is regarded as an adequately rich generative process for ARGs in most settings of interest. While simulating an ARG under this model is fairly straightforward, however, using it to reconstruct an ARG from sequence data is notoriously difficult. Furthermore, the data are generally only weakly informative about the ARG, so it is often desirable to regard it as a “nuisance” variable to be integrated out during statistical inference (e.g., [22]). During the past two decades, various attempts have been made to perform explicit inference of ARGs using techniques such as importance sampling [19], [22] (see also [23]) and Markov chain Monte Carlo sampling [24]–[27]. There is also a considerable literature on heuristic or approximate methods for ARG reconstruction in a parsimony framework [28]–[35]. Several of these approaches have shown promise, but they are generally highly computationally intensive and/or limited in accuracy, and they are not suitable for application to large-scale data sets. As a result, explicit ARG inference is rarely used in applied population genomics.

The coalescent-with-recombination is conventionally described as a stochastic process in time [21], but Wiuf and Hein [36] showed that it could be reformulated as a mathematically equivalent process along the genome sequence. Unlike the process in time, this “sequential” process is not Markovian because long-range dependencies are induced by so-called “trapped” sequences (genetic material nonancestral to the sample flanked by ancestral segments). As a result, the full sequential process is complex and computationally expensive to manipulate. Interestingly, however, simulation processes that simply disregard the non-Markovian features of the sequential process produce collections of sequences that are remarkably consistent in most respects with those generated by the full coalescent-with-recombination [37], [38]. In other words, the coalescent-with-recombination is almost Markovian, in the sense that the long-range correlations induced by trapped material are fairly weak and have a minimal impact on the data. The original Markovian approximation to the full process [37] is known as the *sequentially Markov coalescent* (SMC), and an extension that allows for an additional class of recombinations [38] is known as the SMC'.

In recent years, the SMC has become favorite starting point for approximate methods for ARG inference [39]–[42]. The key insight behind these methods is that, if the continuous state space for the Markov chain (consisting of all possible genealogies) is approximated by a moderately sized finite set—typically by enumerating tree topologies and/or discretizing time—then inference can be performed efficiently using well-known algorithms for hidden Markov models (HMMs). Perhaps the simplest and most elegant example of this approach is the pairwise sequentially Markov coalescent (PSMC) [42], which applies to pairs of homologous chromosomes (typically the two chromosomes in a diploid individual) and is used to reconstruct a profile of effective population sizes over time. In this case, there is only one possible tree topology and one coalescence event to consider at each genomic position, so it is sufficient to discretize time and allow for coalescence within any of 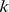 possible time slices. Using the resulting 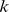-state HMM, it is possible to perform inference integrating over all possible ARGs. A similar HMM-based approach has been used to estimate ancestral effective population sizes and divergence times from individual representatives of a few closely related species [39]–[41]. Because of their dependency on a complete characterization of the SMC state space, however, these methods can only be applied to small numbers of samples. This limits their utility with newly emerging population genomic datasets and leads to reduced power for certain features of interest, such as recent effective population sizes, recombination rates, or local signatures of natural selection.

An alternative modeling approach, with better scaling properties, is the product of approximate conditionals (PAC) or “copying” model of Li and Stephens [43]. The PAC model is motivated primarily by computational tractability and is not based on an explicit evolutionary model. The model generates the 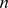
th sequence in a collection by concatenating (noisy) copies of fragments of the previous 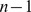 sequences. The source of each copied fragment represents the “closest” (most recently diverged) genome for that segment, and the noise process allows for mutations since the source and destination copies diverged. The PAC framework has been widely used in many applications in statistical genetics, including recombination rate estimation, local ancestry inference, haplotype phasing, and genotype imputation (e.g., [44]–[48]), and it generally offers good performance at minimal computational cost. Recently, Song and colleagues have generalized this framework to make use of conditional sampling distributions (CSDs) based on models closely related to, and in some cases equivalent to, the SMC [49]–[52]. They have demonstrated improved accuracy in conditional likelihood calculations [49], [50] and have shown that their methods can be effective in demographic inference [51], [52]. However, their approach avoids explicit ARG inference and therefore can only be used to characterize properties of the ARG that are directly determined by model parameters (see Discussion).

In this paper, we introduce a new algorithm for ARG inference that combines many of the benefits of the small-sample SMC-based approaches and the large-sample CSD-based methods. Like the PSMC, our algorithm requires no approximations beyond those of the SMC and a discretization of time, but it improves on the PSMC by allowing multiple genome sequences to be considered simultaneously. The key idea of our approach is to sample an ARG of 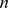 sequences conditional on an ARG of 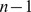 sequences, an operation we call “threading.” Using HMM-based methods, we can efficiently sample new threadings from the exact conditional distribution of interest. By repeatedly removing and re-threading individual sequences, we obtain an efficient Gibbs sampler for ARGs. This basic Gibbs sampler can be improved by including operations that rethread entire subtrees rather than individual sequences. Our implementation of these methods, called *ARGweaver*, is efficient enough to sample full ARGs on a genome-wide scale for dozens of diploid individuals. Simulation experiments indicate that *ARGweaver* converges rapidly and is able to recover many properties of the true ARG with good accuracy. In addition, our explicit characterization of the ARG enables us to examine many features not directly described by model parameters, such as local times to most recent common ancestry, allele ages, and gene tree topologies. These quantities, in turn, shed light on both demographic processes and the influence of natural selection across the genome. For example, we demonstrate, by applying *ARGweaver* to 54 individual human sequences from Complete Genomics, that it provides insight into the sources of reduced nucleotide diversity near functional elements, the contribution of balancing selection to regions containing very old polymorphisms, and the relative influences of direct and indirect selection on allele age. Our *ARGweaver* software (https://github.com/mdrasmus/argweaver), our sampled ARGs (http://compgen.bscb.cornell.edu/ARGweaver/CG_results), and genome-browser tracks summarizing these ARGs (http://genome-mirror.bscb.cornell.edu; assembly hg19) are all freely available.

## Results

### The Sequentially Markov Coalescent

The starting point for our model is the Sequentially Markov Coalescent (SMC) introduced by McVean and Cardin [37]. We begin by briefly reviewing the SMC and introducing notation that will be useful below in describing a general discretized version of this model.

The SMC is a stochastic process for generating a sequence of local trees, 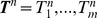 and corresponding genomic breakpoints 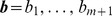, such that each 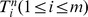 describes the ancestry of a collection of 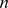 sequences in a nonrecombining genomic interval 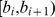, and each breakpoint 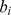 between intervals 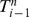 and 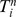 corresponds to a recombination event (Figure 1B). The model is continuous in both space and time, with each node 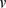 in each 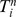 having a real-valued age 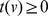 in generations ago, and each breakpoint 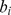 falling in the continuous interval 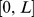, where 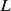 is the total length of the genomic segment of interest in nucleotide sites. The intervals are exhaustive and nonoverlapping, with 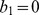, 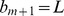, and 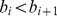 for all 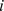. Each 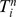 is a binary tree with 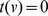 for all leaf nodes 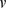. We will use the convention of indexing branches in the trees by their descendant nodes; that is, branch 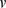 is the branch between node 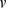 and its parent.

As shown by Wiuf and Hein [36], the correlation structure of the local trees and recombinations under the full coalescent-with-recombination is complex. The SMC approximates this distribution by assuming that 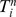 is conditionally independent of 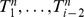 given 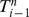, and, similarly, that 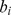 depends only on 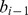 and 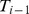, so that,
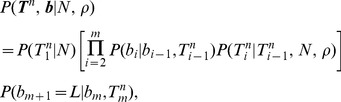
(1)where 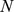 is the effective population size, 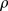 is the recombination rate, and it is understood that 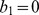. Thus, the SMC can be viewed as generating a sequence of local trees and corresponding breakpoints by a first-order Markov process. The key to the model is to define the conditional distributions 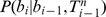 and 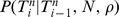 such that this Markov process closely approximates the coalescent-with-recombination. Briefly, this is accomplished by first sampling the initial tree 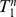 from the standard coalescent and setting 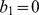, and then iteratively (i) determining the next breakpoint, 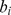, by incrementing 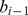 by an exponential random variate with rate 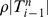, where 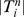 denotes the total branch length of 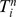; (ii) sampling a recombination point 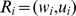 uniformly along the branches beneath the root of 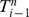, where 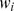 is a branch and 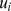 is a time along that branch; (iii) dissolving the branch 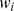 above point 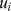; and (iv) allowing 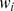 to rejoin the remainder of tree 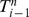 above time 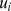 by the standard coalescent process, creating a new tree 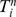 (Figure 1B). As a generative process for an arbitrary number of genomic segments, the SMC can be implemented by simply repeating the iterative process until 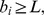 then setting 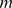 equal to 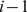 and 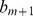 equal to 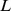.

Notice that, if the sampled recombination points 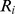 are retained, this process generates not only a sequence of local trees but a complete ARG. In addition, a sampled sequence of local trees, 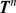, is sufficient for generation of 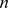 aligned DNA sequences corresponding to the leaves of the trees (Figure 1C). Augmented in this way, the SMC can be considered a full generative model for ARGs and sequence data.

### The Discretized Sequentially Markov Coalescent

We now define an approximation of the SMC that is discrete in both space and time, which we call the Discretized Sequentially Markov Coalescent (DSMC). The DSMC can be viewed as a generalization to multiple genomes of the discretized pairwise sequentially Markov coalescent (PSMC) used by Li and Durbin [42]. It is also closely related to several other recently described discretized Markovian coalescent models [39], [40], [50].

The DSMC assumes that time is partitioned into 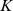 intervals, whose boundaries are given by a sequence of time points 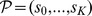, with 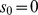, 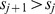 for all 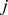 (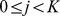), and 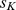 equal to a user-specified maximum value. (See Table 1 for a key to the notation used in this paper.) Every coalescence or recombination event is assumed to occur precisely at one of these 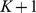 time points. Various strategies can be used to determine these time points (see, e.g., [50]). In this paper, we simply distribute them uniformly on a logarithmic scale, so that the resolution of the discretization scheme is finest near the leaves of the ARG, where the density of events is expected to be greatest (see Methods). Each local block is assumed to have an integral length measured in base pairs, with all recombinations occurring between adjacent nucleotides. The DSMC approaches the SMC as the number of intervals 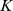 and the sequence length 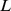 grow large, for fixed 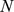 and 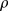.

**Table 1:** Key to notation.

**Population Genetic Parameters**	
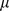	Mutation rate, in events per site per generation
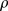	Recombination rate, in events per site per generation
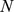	Effective population size, in number of individuals^a^
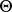	Full parameter set, 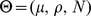
**Time Discretization**	
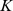	Total number of time intervals (user-defined)
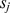	Time point 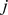 ( 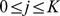 ), defining a boundary between time intervals (generations before present)
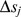	Length of 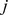 th time interval, 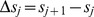
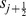	Midpoint of 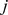 th time interval
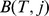	Set of branches in a tree 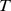 associated with time interval 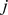
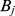	Number of branches associated with time interval 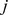 , 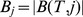 (with 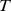 determined by context)
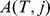	Set of “active” branches at time point 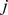
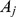	Number of “active” branches at time point 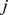 , 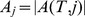 (with 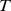 determined by context)
**Ancestral Recombination Graph**	
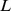	Length of analyzed sequence alignment in nucleotides
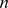	Number of sequences in alignment
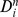	Alignment column at 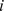 th position; cumulatively, 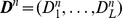
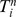	Local tree for 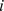 th position; cumulatively, 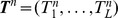
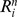	Recombination point between 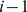 st and 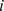 th position; cumulatively, 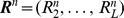
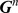	Full ARG for 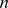 sequences, 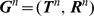
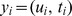	Coalescence point for threaded sequence at 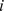 th position, defined by a branch 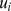 and a time point 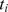 ; cumulatively, 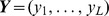
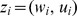	Recombination point for threaded sequence between positions 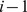 and 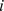 , defined by a branch 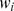 and a time point 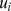 ; cumulatively, 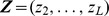
**Hidden Markov Model**	
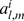	Transition probability from state 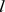 to state 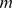 between position 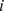 and 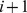
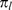	Initial state probability for state 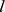
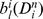	Emission probability for alignment column 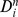 in state 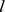 at position 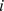

a Model allows for a separate 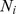 for each time interval l but all analyses in this paper assume a constant 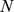 across time intervals.

Like the SMC, the DSMC generates an ARG 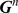 for 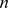 (haploid) sequences, each containing 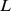 nucleotides (Figure 1B). In the discrete setting, it is convenient to define local trees and recombination events at the level of individual nucleotide positions. Assuming that 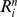 denotes a recombination between 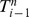 and 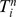, we write 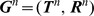, with 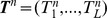 for positions 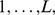 and 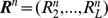. Notice that it is possible in this setting that 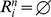 and 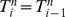. Where a recombination occurs (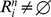), we write 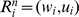 where 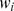 is the branch in 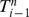 and 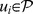 is the time point of the recombination. For simplicity and computational efficiency, we assume that at most one recombination occurs between each pair of adjacent sites. Given the sparsity of variant sites in most data sets, this simplification is likely to have, at most, a minor effect during inference (see Discussion).

Like the SMC, the DSMC can additionally be used to generate an alignment of DNA sequences (Figure 1C). We denote such an alignment by 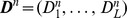, where each 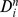 represents an alignment column of height 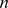. Each 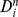 can be generated, in the ordinary way, by sampling an ancestral allele from an appropriate background distribution, and then allowing this allele to mutate stochastically along the branches of the corresponding local tree, in a branch-length-dependent manner. We denote the induced conditional probability distribution over alignment columns by 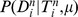, where 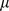 is the mutation rate. In this work, we assume a Jukes-Cantor model [53] for nucleotide mutations along the branches of the tree, but another mutation model can easily be used instead. Notice that, while the recombinations 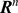 are required to define the ARG completely, the probability of the sequence data given the ARG depends only on the local trees 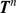.

### The Threading Problem

In the case of an observed alignment, 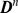, and an unobserved ARG, 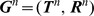, the DSMC can be viewed as a hidden Markov model (HMM) with a state space given by all possible local trees, transition probabilities given by expressions of the form 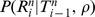 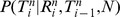, and emission probabilities given by the conditional distributions for alignment columns, 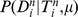. The complete data likelihood function of this model—that is, the joint probability of an ARG 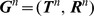 and a sequence alignment 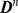 given model parameters 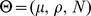
—can be expressed as a product of these terms over alignment positions (see Methods for further details):
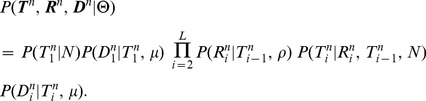
(2)This HMM formulation is impractical as a framework for direct inference, however, because the set of possible local trees—and hence the state space—grows super-exponentially with 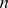. Even with additional assumptions, similar approaches have only been able to accommodate small numbers of sequences [32], [35], [54].

**Figure 2:**
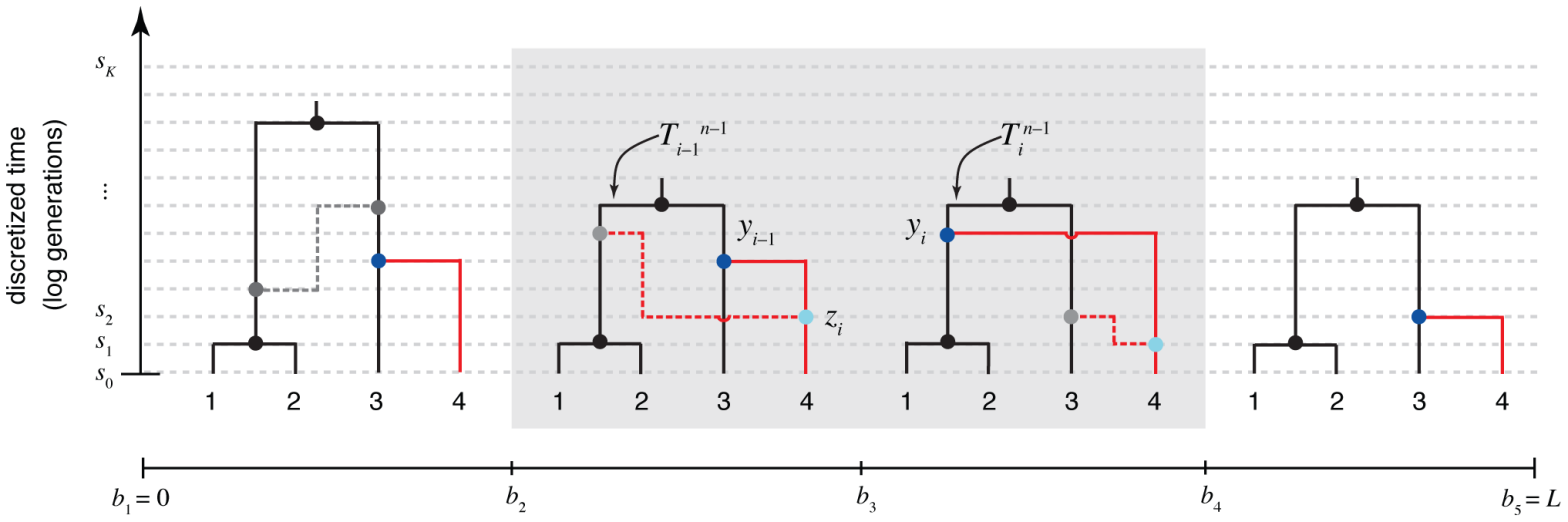
The “threading” operation. The threading operation adds an 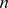
th sequence to an ARG of 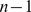 sequences under a discretized version of the SMC (the DSMC) that requires all coalescence and recombination events to occur precisely at pre-defined time points, 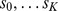 (horizontal dashed lines). In this example, the fourth sequence has been removed from ARG 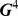 from Figure 1, leaving a tree with 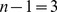 leaves at each position 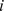 (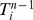; shown in black). The fourth sequence (shown in red) is re-threaded through the remaining portion of the ARG by a two-step process that first samples a coalescence point 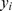 for this sequence at each 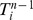 (dark blue points), thereby defining a new tree 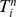, and second, samples a recombination point 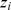 to reconcile each adjacent pair of trees, 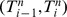 (light blue points). For simplicity, only the distinct local trees for the four nonrecombining segments (after threading) are shown. The gray box highlights the pair of trees immediately flanking the breakpoint 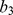. Notice that the first recombination from Figure 1 is retained (dark gray nodes and dashed line in left-most tree). In general, new recombinations are prohibited at the locations of “given” recombinations 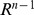 (see text). Note that it is possible for the attachment point of the 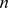
th sequence in the local trees to move due to old recombinations as well as new ones (not shown in this example).

Instead, we use an alternative strategy with better scaling properties. The key idea of our approach is to sample the ancestry of only one sequence at a time, while conditioning on the ancestry of the other 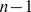 sequences. Repeated applications of this “threading” operation form the basis of a Markov chain Monte Carlo sampler that explores the posterior distribution of ARGs. In essence, the threading operation adds one branch to each local tree in a manner that is consistent with the assumed recombination process and the observed data (Figure 2). While conditioning on a given set of local trees introduces a number of technical challenges, the Markovian properties of the DSMC are retained in the threading problem, and it can be solved using standard dynamic programming algorithms for HMMs.

The threading problem can be precisely described as follows. Assume we are given an ARG for 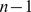 sequences, 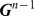, a corresponding data set 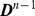, and a set of model parameters 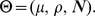 Assume further that 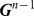 is consistent with the assumptions of the DSMC (for example, all of its recombination and coalescent events occur at time points in 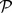 and it contains at most one recombination per position). Finally, assume that we are given an 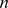
th sequence 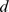, of the same length of the others, and let 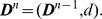 The threading problem is to sample a new ARG 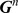 from the conditional distribution 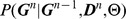 under the DSMC.

The problem is simplified by recognizing that 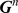 can be defined by augmenting 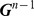 with the additional recombination and coalescence events required for the 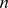
th sequence. First, let 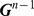 be represented in terms of its local trees and recombination points: 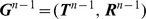. Now, observe that specifying the new coalescence events in 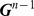 is equivalent to adding one branch to each local tree, 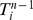 for 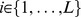, to obtain a new tree 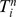 (Figure 2). Let us denote the point at which each of these new branches attaches to the smaller subtree at each genomic position 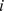 by 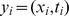, where 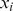 indicates a branch in 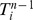 and 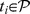 indicates the coalescence time along that branch. Thus, the *coalescence threading* of the 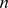
th sequence is given by the sequence 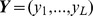.

**Figure 3:**
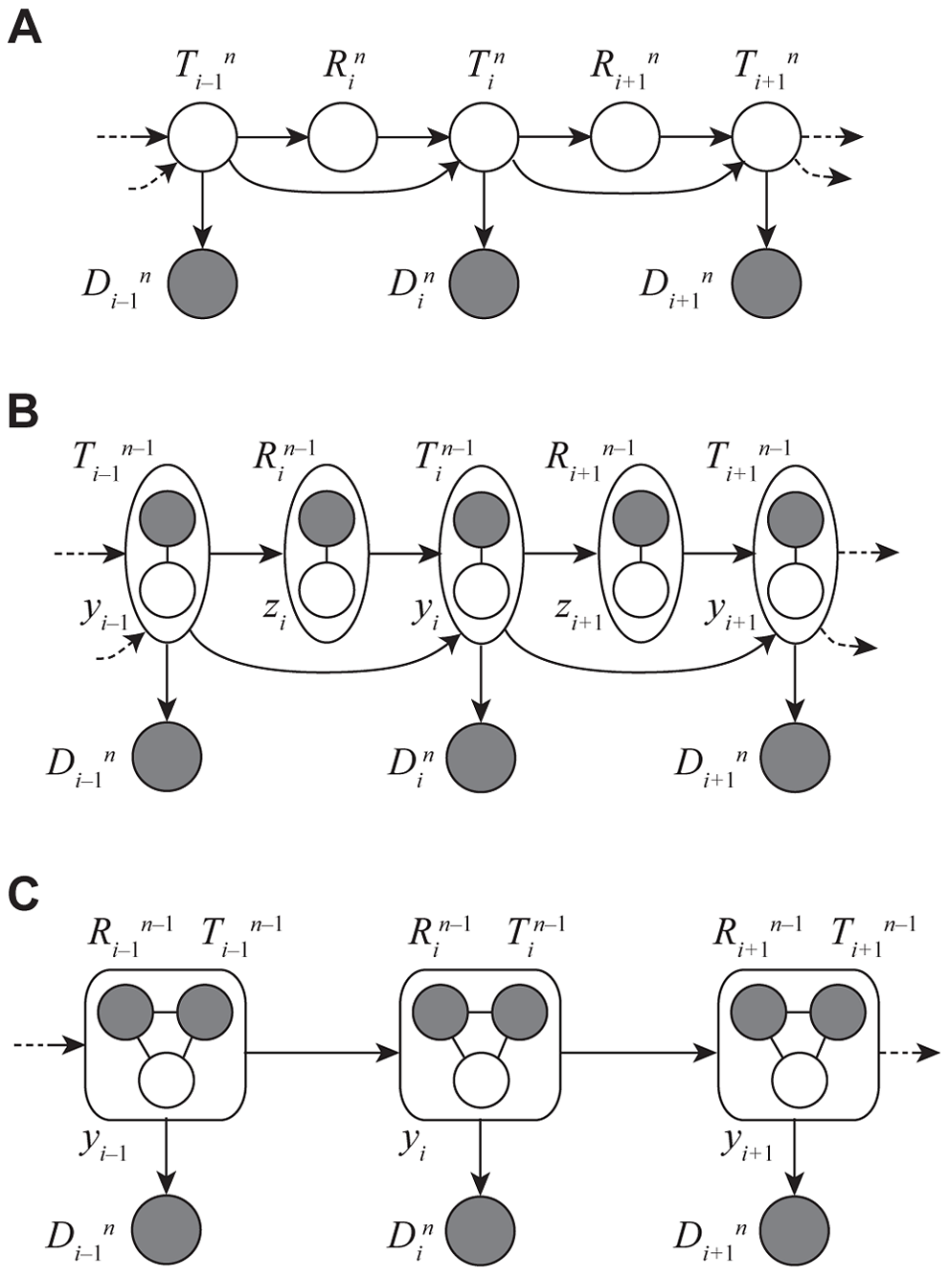
Graphical models for Discretized Sequentially Markov Coalescent (DSMC) models. (A) Full DSMC model for 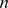 samples with local trees, 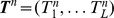, recombinations, 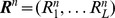, and alignment columns, 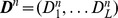. Together, 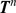 and 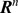 define an ancestral recombination graph, 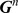. Solid circles indicate observed variables and empty circles indicate latent variables. Arrows indicate direct dependencies between variables and correspond to conditional probability distributions described in the text. Notice that the 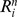 variables can be integrated out of this model, leading to the conventional graph topology for a hidden Markov model. (B) The same model as in (A), but now partitioning the latent variables into components that describe the history of the first 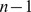 sequences (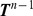 and 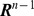) and components specific to the 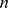
th sequence (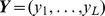 and 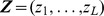). The 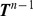 and 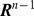 variables are represented by solid circles because they are now “clamped” at specific values. A sample of 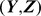 represents a threading of the 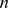
th sequence through the ARG. (C) Reduced model after elimination of 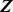 by integration, enabling efficient sampling of coalescent threadings 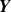. This is the model used by the first step in our two-step sampling approach. In the second step, the 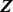 variables are sampled conditional on 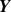, separately for each 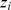. In this model, the grouped nodes have complex joint dependencies, leading to a heterogeneous state space and normalization structure, but the linear conditional independence structure of an HMM is retained.

To complete the definition of 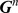, we must also specify the precise locations of the additional recombinations associated with the threading—that is, the specific time point at which each branch in a local tree 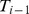 was broken before the branch was allowed to re-coalesce in a new location in tree 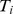. Here it is useful to partition the recombinations into those that are given by 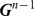, denoted 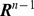, and those new to 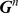, which we denote 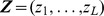 (Figure 3A&B). Each 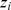 is either null (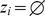), meaning that there is no new recombination between 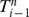 and 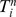, or defined by 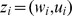, where 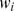 is a branch in 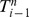 and 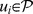 is the time along that branch at which the recombination occurred. We call 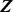 the *recombination threading* of the 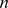
th sequence. For reasons of efficiency, we take a two-step approach to threading: first, we sample the coalescence threading 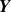, and second, we sample the recombination threading 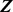 conditional on 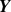. This separation into two steps allows for a substantially reduced state space during the coalescence threading operation, leading to significant savings in computation. When sampling the coalescence threading (step one), we integrate over the locations of the new recombinations 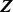, as in previous work [42], [50]. Sampling the recombination threading (step two) can be accomplished in a straightforward manner independently for each recombination event, by taking advantage of the conditional independence structure of the DSMC model (see Methods for details).

The core problem, then, is to accomplish step one by sampling the coalescence threading 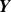 from the distribution,
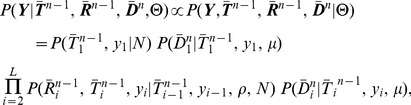
(3)where the notation 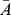 indicates that random variable 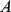 is held fixed (“clamped”) at a particular value throughout the procedure. This equation defines a hidden Markov model with a state space given by the possible values of each 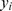, transition probabilities given by 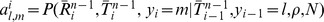 and emission probabilities given by 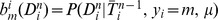 (Figure 3C). Notice that the location of each new recombination, 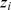, is implicitly integrated out in the definition of 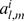. Despite some unusual features of this model—for example, it has a heterogeneous state space and normalization structure along the sequence—its Markovian dependency structure is retained, and the problem of drawing a coalescent threading 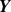 from the desired conditional distribution can be solved exactly by dynamic programming using the stochastic traceback algorithm for HMMs. Additional optimizations allow this step to be completed in time linear in both the number of sequences 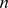 and the alignment length 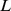 and quadratic only in the number of time intervals 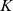 (see Methods for details).

### Markov Chain Monte Carlo Sampling

The main value of the threading operation is in its usefulness as a building block for Markov chain Monte Carlo methods for sampling from an approximate posterior distribution over ARGs given the data. We employ three main types of sampling algorithms based on threading, as described below.

#### Sequential sampling

First, the threading operation can be applied iteratively to a series of orthologous sequences to obtain an ARG of size 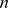 from sequence data alone. This method works by randomly choosing one sequence and constructing for it a trivial ARG 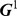 (i.e. every local tree is a single branch). Additional sequences are then threaded into the ARG, one at a time, until an ARG 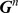 of 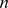 sequences has been obtained. Notice that an ARG derived in this manner is not a valid sample from the posterior distribution, because each successive 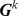 (for 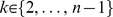) is sampled conditional on only 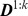 (the first 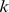 sequences). Nevertheless, the sequential sampling algorithm is an efficient heuristic method for obtaining an initial ARG, which can subsequently be improved by other methods. If desired, this operation can be applied multiple times, possibly with various permutations of the sequences, to obtain multiple initializations of an MCMC sampler. Heuristic methods can also be used to choose a “smart” initial ordering of sequences. For example, one might begin with one representative of each of several populations, to first approximate the overall ARG structure, and subsequently add more representatives of each population.

#### Gibbs sampling for single sequences

Second, the threading operation can serve as the basis of a Gibbs sampler for full ARGs. Starting with an initial ARG of 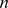 sequences, individual sequences can be removed, randomly or in round-robin fashion, and rethreaded. Since the threading procedure samples from the conditional distribution 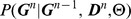, this produces a valid Gibbs sampler for the ARG up to the assumptions of the DSMC. The ergodicity of the Markov chain follows, essentially, from the fact that any tree is reachable from any other by a finite sequence of branch removals and additions (see Text S1 for details).

The main limitation of this method is that it leads to poor mixing when the number of sequences grows large. The essential problem is that rethreading a single sequence is equivalent to resampling the placement of external branches in the local trees, so this method is highly inefficient at rearranging the “deep structure” (internal branches) of the ARG. Furthermore, this mixing problem becomes progressively worse as 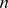 grows larger. Indeed, as 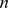 approaches infinity, the single-sequence threading operation reduces to a procedure that selects a sequences of short genealogy “tips” leading to other sequences in the data set, leaving all other aspects of the ARG unchanged; in effect, it approaches the “copying” model of Li and Stephens [43]. As a result, an alternative strategy for ARG sampling is needed for large numbers of sequences.

#### Subtree sampling

The third sampling strategy addresses the mixing limitations of the single-sequence Gibbs sampler by generalizing the threading operation to accommodate not only individual sequences but subtrees with arbitrary numbers of leaves. As a result, internal branches in the local trees can be resampled and the full ARG can be perturbed, including the deep branches near the roots of the local trees.

In principle, one could address the subtree threading problem by arbitrarily selecting an internal branch for each nonrecombining segment of the ARG and resampling its attachment point to the remainder of the tree, by essentially the same procedure used for the reattachment of external branches in single-sequence threading. The problem is that, because the local trees change along the sequence, it is impossible in general to select a sequence of internal branches whose subtrees are maintained across the entire ARG (this is possible only for external branches). Furthermore, if a poor sequence of internal branches is selected, the attachment points at *both* ends of each segment will be constrained by the flanking local trees, creating a strong tendency to resample the original attachment points, which would result in poor mixing of the sampler.

To address this problem, we devised a novel method for selecting sequences of subtrees guaranteed to have good continuity properties. Once such a sequence is selected, the subtree threading operation can be accomplished efficiently using the stochastic traceback algorithm, in a similar manner as with single sequences. Our algorithm for selecting sequences of internal branches is fairly technical in nature and a detailed description is left for Text S1. Briefly, to select sequences of subtrees, we use a data structure called a *branch graph*, which traces the shared ancestry among branches across genomic positions. Using dynamic programming, we are able to identify paths through the branch graph that correspond to sequences of internal branches with good continuity properties. After a sequence of internal branches is identified, the selected branch is removed from each local tree, splitting it into a main tree and a subtree. A new branch is then added above the root of every subtree and allowed to re-coalesce with the corresponding main tree in a manner consistent with the DSMC.

One important limitation of the algorithm is worth noting. As in the single-sequence case, the stochastic traceback algorithm samples from the desired conditional distribution over subtree threadings. However, since the number of ways of removing internal branches depends on the current structure of the ARG, the Hastings ratio is not equal to one in this case, and a more general Metropolis-Hastings algorithm (with rejection of some proposed threadings) is required (see Text S1 for details). In practice, the acceptance rates for proposed threadings are fairly high (∼40% for typical human data), and despite this limitation, Metropolis-Hastings subtree threading considerably improves the mixing properties of the Gibbs sampler for moderately large values of 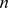 (see below).

### *ARGweaver* Program and Visualization

We implemented these sampling strategies in a computer program called *ARGweaver*, that “weaves” together an ARG by repeated applications of the threading operation. The program has subroutines for threading of both individual sequences and subtrees. Options allow it to be run as a Gibbs sampler with single-sequence threading or a general Metropolis-Hastings sampler with subtree threading. In either case, sequential sampling is used to obtain an initial ARG. Options to the program specify the number of sampling iterations and the frequency with which samples are recorded. The program is written in a combination of C++ and Python and is reasonably well optimized. For example, it requires about 1 second to sample a threading of a single 1 Mb sequence in an ARG of 20 sequences with 20 time steps. Our source code is freely available via GitHub (https://github.com/mdrasmus/argweaver).

To summarize and visualize samples from the posterior distribution over ARGs, we use two main strategies. First, we summarize the sampled ARGs in terms of the time to most recent common ancestor (TMRCA) and total branch length at each position along the genome. We also consider the estimated age of the derived alleles at polymorphic sites, which we obtain by mapping the mutation to a branch in the local tree and calculating the average time for that branch (see Methods). We compute posterior mean and 95% credible intervals for each of these statistics per genomic position, and create genome browser tracks that allow these values to be visualized together with other genomic annotations.

Second, we developed a novel visualization device for ARGs called a “leaf trace.” A leaf trace contains a line for each haploid sequence in an analyzed data set. These lines are ordered according to the local genealogy at each position in the genome, and the spacing between adjacent lines is proportional to their TMRCAs (Figure S2). The lines are parallel in nonrecombining segments of the genome, and change in order or spacing where recombinations occur. As a result, several features of interest are immediately evident from a leaf trace. For example, recombination hot spots show up as regions with dense clusters of vertical lines, whereas recombination cold spots are indicated by long blocks of parallel lines.

### Simulation Study

#### Effects of discretization and convergence of sampler

Before turning to inference, we performed a series of preliminary experiments to verify that our discretization strategy allowed for an adequate fit to the data and that *ARGweaver* converged to a plausible posterior distribution for realistic simulated data sets. Briefly, we found that the DSMC produces similar numbers of recombination counts and segregating sites as the coalescent-with-recombination and SMC, when generating data under various recombination rates and effective population sizes (see Text S1 and Supplementary Figure S1). With small numbers of sequences, the Gibbs sampler based on the single-sequence threading operation appeared to converge rapidly, according to both the log likelihood of the sampled ARG and the inferred numbers of recombination events. When the number of sequences grew larger than about 6–8 (depending on the specific details of the simulation), the Gibbs sampling strategy was no longer adequate. However, the subtree threading operation and Metropolis-Hastings sampler appeared to address this problem effectively, allowing the number of sequences to be pushed to 20 or more. With 20 sequences 1 Mb in length, the sampler converges within about 500 sampling iterations, which takes about 20 minutes on a typical desktop computer (Supplementary Figure S3).

#### Recovery of global ARG features

Next, we systematically assessed the ability of *ARGweaver* to recover several features of interest from simulated ARGs over a range of plausible ratios of mutation to recombination rates (see Methods for simulation parameters). In these experiments, we considered three “global” features of the ARG: (i) the log joint probability of the ARG and the data (log of equation 2), (ii) the total number of recombinations, and (iii) the total branch length of the ARG. We define the total branch length of the ARG to be the sum of the total branch lengths of the local trees at all sites (in generations), a quantity proportional to the expected number of mutations in the history of the sample. We applied *ARGweaver* to each simulated data set with 500 burn-in iterations, followed by 1000 sampling iterations, with every tenth sample retained (100 samples total).

**Figure 4:**
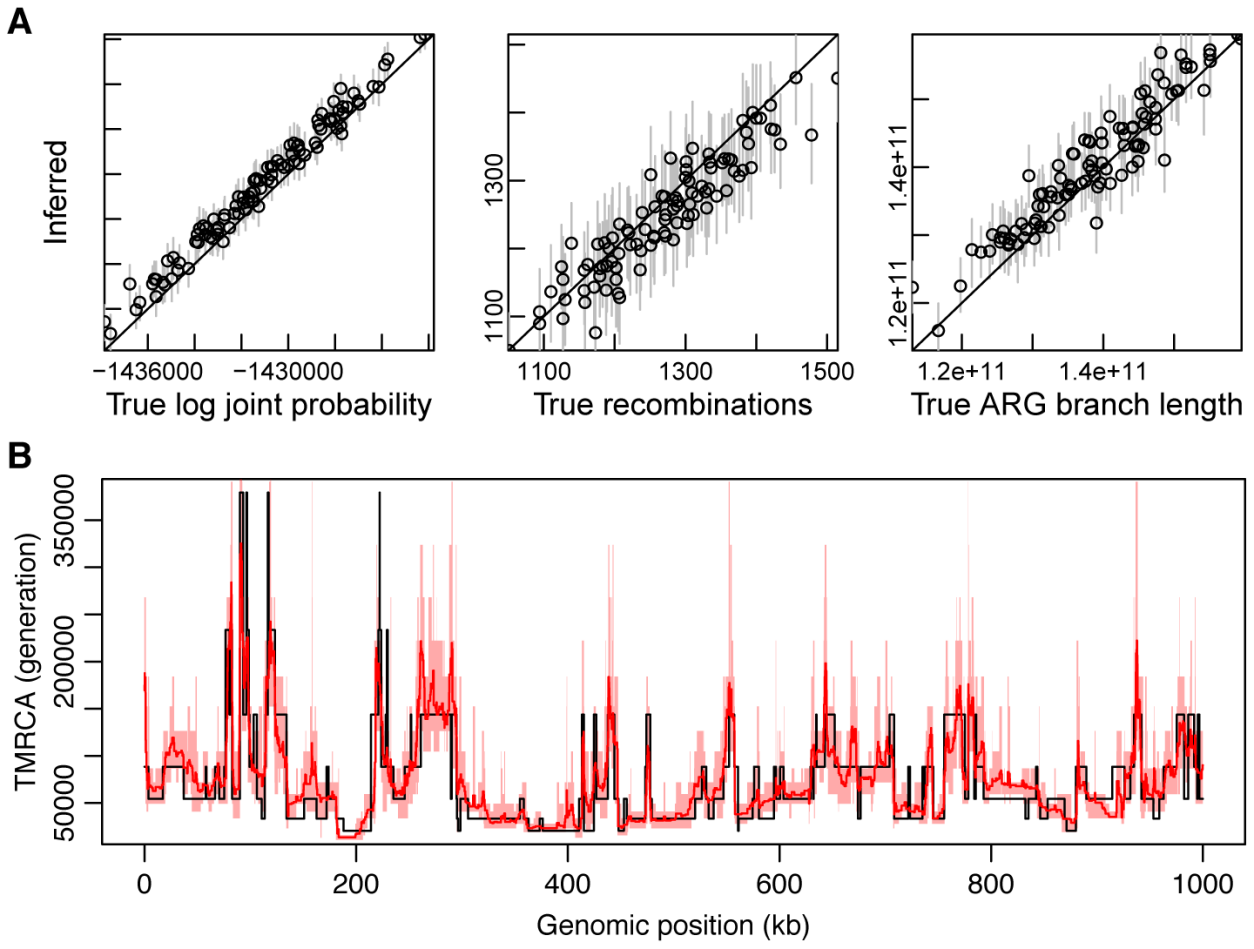
Simulation results. (A) Recovery of global features of simulated ARGs from sequence data. This plot is based on sets of 20 1-Mb sequences generated under our standard simulation parameters (see Methods) with 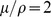 (see Supplementary Figure 10 for additional results). From left to right are shown true (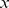-axis) versus inferred (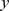-axis) values of the log joint probability (the logarithm of equation 2), the total number of recombinations, and the total branch length of the ARG. Each data point in each plot represents one of 100 simulated data sets. In the vertical dimension, circles represent averages across 100 sampled ARGs based on the corresponding data sets, sampled at intervals of 10 after a burn-in of 200 iterations, and error bars represent the interval between the 2.5 and 97.5 percentiles. In the second and third plots, circles are interpretable as posterior expected values and error bars as 95% Bayesian credible intervals. (B) Posterior mean TMRCA (dark red line, with 95% credible intervals in light red) versus true TMRCA (black line) along a simulated genomic segment of 1 Mb. This plot is based on a single representative data set of 20 1-Mb sequences generated under our standard simulation parameters with 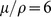 (see Supplementary Figure S5 for additional results).

We found that *ARGweaver* was able to recover the features of interest with fairly high accuracy at all parameter settings (Figure 4A and Supplementary Figure S4). In addition, the variance of our estimates is generally fairly low, but does show a clear reduction as 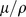 increases from 1 to 6, corresponding to an increase in the phylogenetic information per nonrecombining segment. Most current estimates of average rates would place the true value of 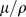 for human populations between 1 and 2 [55]–[57], but the concentration of recombination events in hot spots implies that the ratio should be considerably more favorable for our methods across most of the genome. Notably, we do observe a slight tendency to under-estimate the number of recombinations, particularly at low values of 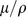. This underestimation is paired with an over-estimation of the joint probability (left column), suggesting that it reflects model misspecification of the DSMC. It is possible that this bias could be improved by the use of the SMC' rather than the SMC, or by a finer-grained discretization scheme (see Discussion).

#### Recovery of local ARG features

An advantage of explicitly sampling full ARGs is that it enables inferences about local features of the ARG that are not directly determined by model parameters. Using the same simulated data and inference procedure as in the previous section, we evaluated the performance of *ARGweaver* in estimating three representative quantities along the genome sequence: (i) time to most recent common ancestry (TMRCA), (ii) recombination rate, and (iii) allele age. We estimated each quantity using an approximate posterior expected value, computed by averaging across sampled ARGs. With 20 sequences, we found that *ARGweaver* was able to recover the TMRCA with fairly high accuracy and resolution (Figure 4B). The quality of the estimates degrades somewhat at lower values of the ratio 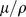 but remains quite good even with 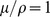 (Supplementary Figure S5). We found that our power for recombination rates was weak with only 20 sequences, but with 100 sequences the reconstructed ARGs clearly displayed elevated rates of recombination in simulated hotspots compared with the flanking regions (Supplementary Figure S6). Estimates of allele ages appeared to be unbiased, with good concordance between true and estimated values, although the variance in the estimates was fairly high (Supplementary Figure S7, left column). Notably, the ARG-based estimates of allele age appear to be considerably better than estimates based on allele frequency alone (Supplementary Figure S7, right column). Together, these results suggest that, even with modest numbers of sequences, the distributions of ARGs inferred by our methods may be informative about loci under natural selection, local recombination rates, and other local features of evolutionary history.

#### Accuracy of local tree topologies

In our next experiment, we evaluated the accuracy of *ARGweaver* in inferring the topology of the local trees, again using the same simulated data. The local trees are a more complex feature of the ARG but are of particular interest for applications such as genotype imputation and association mapping. For comparison, we also inferred local trees using the heuristic *Margarita* program [34], which is, to our knowledge, the only other published ARG-inference method that can be applied at this scale. In addition, we applied an unpublished method, called *treesim* (http://niallcardin.com/treesim/index.html), that samples genealogies using heuristic extensions of the Monte Carlo methods of Fearnhead and Donnelly [22]. To compare these programs, we identified 100 evenly spaced locations in our simulated data sets, and extracted the local trees reconstructed by all three methods at these positions. We found that *ARGweaver* produced more accurate local tree topologies than both *Margarita* and *treesim* across most values of 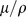, except for the case of 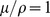, where *treesim* performed slightly better (Supplementary Figure S8). The improvements were most pronounced at high 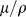 values, where topological information is greatest. In addition, the absolute accuracy of the trees inferred by *ARGweaver* was fairly high, given the sparseness of informative sites in these data sets. For example, at 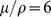, more than 80% of predicted branches were correct and Maximum Agreement Subtree (MAST) percentages approached 75%, and even in the challenging case of 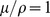, over 60% of branches were correct and MAST percentages exceeded 50%. These results indicate that the sampler is effectively pooling information from many sites across the multiple alignment in making inferences about local tree topologies.

Finally, we evaluated the accuracy of *ARGweaver*'s assessment of the uncertainty about the local trees given the data. We grouped individual branches into bins according to their estimated posterior probabilities (i.e., the fraction of sampled local trees in which each branch is found), and compared these values with the relative frequencies with which the same branches were observed in the true trees. We found that the predicted and actual probabilities of correctness were closely correlated, indicating that *ARGweaver* is accurately measuring the uncertainty associated with the local trees (Supplementary Figure S9). By contrast, the heuristic *Margarita* sampler shows a clear tendency to overestimate the confidence associated with branches in the local trees, often by 10–20%. This comparison is not entirely fair, because the authors of *Margarita* do not claim that it samples from the posterior distribution, but it nevertheless highlights an important advantage of the Bayesian approach. Notably, the unpublished *treesim* program performed remarkably well on this test.

### Analysis of Real Data

Having demonstrated that *ARGweaver* was able to recover many features of simulated ARGs with reasonable accuracy, we turned to an analysis of real human genome sequences. For this analysis we chose to focus on sequences for 54 unrelated individuals from the “69 genomes” data set from Complete Genomics (http://www.completegenomics.com/public-data/69-Genomes) [58]. The 54 genome sequences were computationally phased using SHAPEIT v2 [59] and were filtered in various ways to minimize the influence from alignment and genotype-calling errors. They were partitioned into ∼2-Mb blocks and *ARGweaver* was applied to these blocks in parallel using the Extreme Science and Engineering Discovery Environment (XSEDE). For this analysis, we assumed 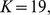 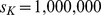 generations, 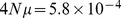, and 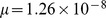, implying 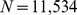. We allowed for variation across loci in mutation and recombination rates. For each ∼2-Mb block, we collected samples for 2,000 iterations of the sampler and retained every tenth sample, after an appropriate burn-in (see Methods for complete details). The entire procedure took ∼36 hours for each of the 1,376 2-Mb blocks, or 5.7 CPU-years of total compute time. The sampled ARGs were summarized by UCSC Genome Browser tracks describing site-specific times to most recent common ancestry (TMRCA), total branch length, allele ages, leaf traces, and other features across the human genome. These tracks are publicly available from our local mirror of the UCSC Genome Browser (http://genome-mirror.bscb.cornell.edu, assembly hg19).

#### Distortions in the ARG due to natural selection

**Figure 5:**
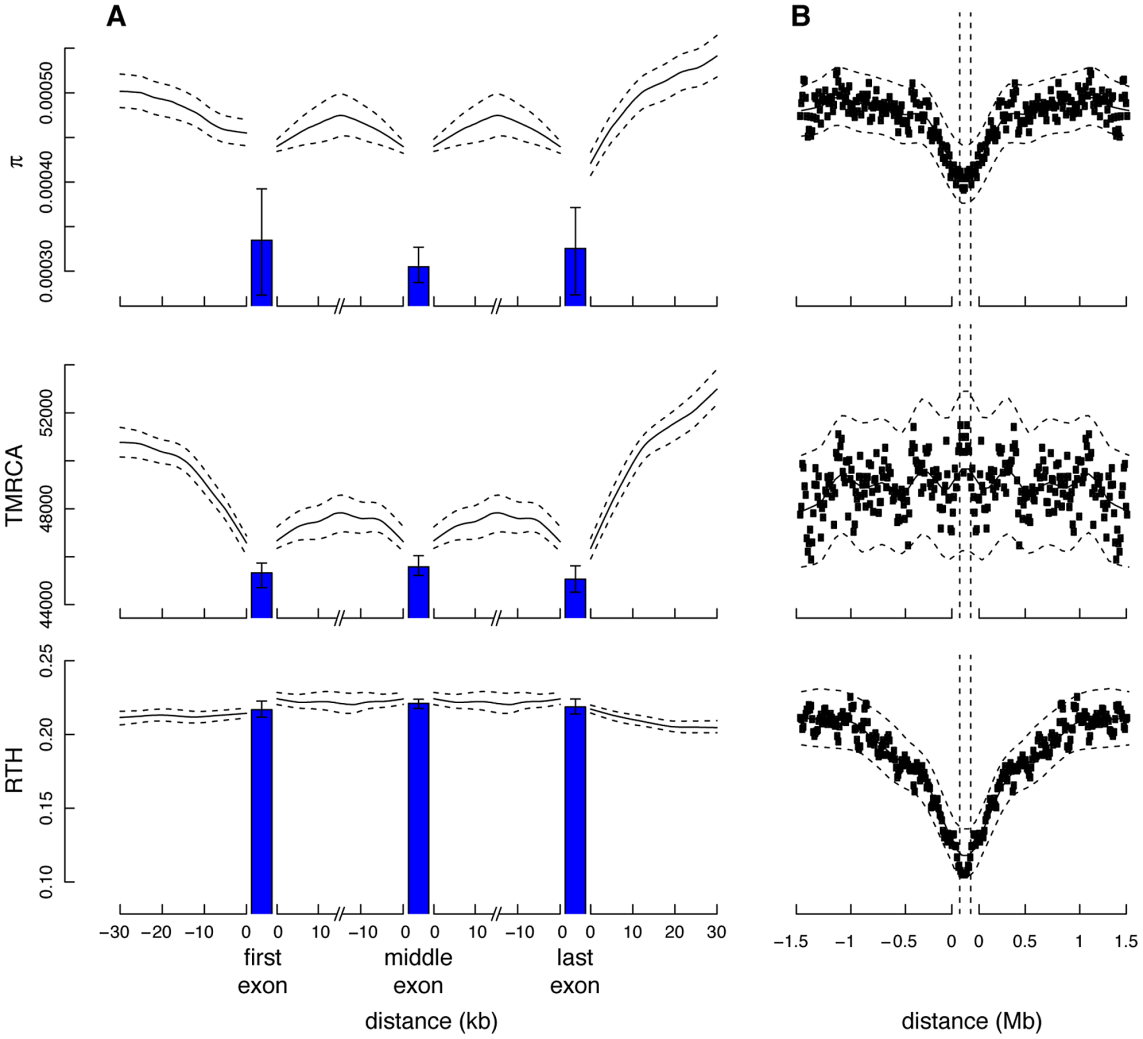
Measures of genetic variation near protein-coding genes and partial selective sweeps. Shown (from top to bottom) are nucleotide diversity (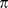), time to most recent common ancestry (TMRCA), and relative TMRCA halflife (RTH) for the 13 individuals (26 haploid genomes) of European descent (CEU and TSI populations) in the Complete Genomics data set (similar plots for African population are shown in Supplementary Figure S11). Nucleotide diversity 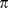 was computed as the average rate of nucleotide differences per site across all pairs of chromosomes, whereas sitewise values of the TMRCA and RTH were computed by averaging over local trees sampled by *ARGweaver*. (A) Estimates for 17,845 protein-coding genes from the Consensus Coding Sequence (CCDS) track in the UCSC Genome Browser (hg19). Estimates for noncoding regions were computed by averaging in a sliding window of size 300 bp then averaging across genes. Estimates for coding exons were computed by first averaging over fourfold degenerate (4d) sites of each exonic type (first, middle, last), then averaging across genes (see Methods). Only 4d sites were considered to focus on the influence of selection from linked sites rather than direct selection. Nevertheless, the decreased values for the exons suggest some influence from direct selection. The differences between exons and flanking sites may also be influenced by windowing in the noncoding regions. “First exon” is taken to begin at the annotated start codon and “last exon” to end at the stop codon, so that both exclude untranslated regions. The TMRCA is measured in thousands of generations. RTH is ratio of the time required for the first 50% of lineages to find a most recent common ancestor to the full TMRCA (see Supplementary Figure S10). Error bars (dashed lines for noncoding regions) indicate 95% confidence intervals as estimated by bootstrapping over regions. (B) Similar plots for 255 100-kb regions predicted to have undergone partial selective sweeps in the CEU population based on the iHS statistic [72]. In this case, all measures are computed in a sliding window of 10,000 bases. Notice that both protein-coding genes and putative selective sweeps display substantial reductions in nucleotide diversity, but the genes show a much more prominent reduction in TMRCA, whereas the sweeps show a much more prominent reduction in RTH. These signatures are consistent with a dominant influence from background selection rather than hitchhiking in protein-coding genes (see text).

While our prior distribution over ARGs is based on the neutral coalescent, we were interested in exploring whether natural selection produces a sufficiently strong signal in the data to create detectable distortions in the ARG near functional elements. We began by examining the estimated posterior expected values of the TMRCA around known protein-coding genes, focusing on fourfold degenerate (4d) sites within coding exons and noncoding sites flanking exons. For comparison with our ARG-based measures, we also computed a simple measure of nucleotide diversity, 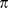. Both 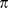 and the ARG-based TMRCA behave in a qualitatively similar manner near genes, achieving minimal values in coding exons and gradually increasing with distance from exon boundaries (Figure 5A). These observations are consistent with several recent studies indicating reduced neutral diversity near both coding and noncoding functional elements, which has been attributed to indirect effects from selection at linked sites [60]–[64]. However, it has been difficult to distinguish between two alternative modes of selection both predicted to have similar influences on patterns of neutral diversity: “background selection” (BGS) associated with negative or purifying selection at linked sites [65]–[68], and “hitchhiking” (HH) (selective sweeps) associated with linked mutations under positive selection [69]. In principle, explicit ARG inference could help to resolve this controversy, because BGS and HH lead to different predictions for the structure of genealogies (e.g., [70], [71]).

To examine these questions further, we computed the same statistics for 255 putative partial selective sweeps identified in CEU populations and 271 partial sweeps identified in YRI populations based on the integrated extended haplotype homozygosity statistic (iHS) [72]. As expected, the sweep regions were broadly similar to the protein-coding genes in terms of nucleotide diversity 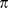 (Figure 5B). However, unlike the protein-coding genes, the sweep regions displayed no clear depression in TMRCA. One possible way of understanding this observation is that, while sweeps tend to be enriched overall for recent coalescence events (as indicated by the reductions in 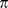), the oldest coalescence events are relatively unaffected by selective sweeps, perhaps because some lineages tend to “escape” each sweep, leading to near-neutral patterns of coalescence near the roots of genealogies (where the contribution to the TMRCA is greatest). This may be particularly true for the partial sweeps identified by the iHS method, but a similar phenomenon should occur in flanking regions of the causal mutations for complete sweeps. BGS, by contrast, is expected to affect both the total branch length and TMRCA approximately equally, by effectively reducing the time scale of the coalescence process, but to have a minimal influence on the relative intervals between coalescence events.

In an attempt to distinguish further between BGS and HH, we introduced a statistic called the *relative TMRCA halflife* (RTH), defined as the ratio between the time to most recent common ancestry for the first 50% of chromosomes and the full TMRCA. The RTH captures the degree to which coalescence events are skewed toward the recent past, in a manner that does not depend on the overall rate of coalescence. Thus, the RTH should be relatively insensitive to BGS, but sensitive to HH if, as proposed above, sweeps tend to affect many but not all lineages (see Supplementary Figure S10). In the European populations, the statistic showed a pronounced valley near selective sweeps (Figure 5B), as expected, but it was much more constant across genic regions (Figure 5A). Its behavior was similar in the African populations, except that it showed somewhat more variability near genes, yet in an opposite pattern from the sweeps (Supplementary Figure S11). Overall, these results suggest that, while the total rate of coalescence differs substantially across genic regions, the relative depths of middle and extreme coalescence events do not, on average, consistent with the predictions of a model in which BGS dominates in genes [60], [62], [64]. The sharply contrasting patterns for the iHS-identified sweeps suggest that partial sweeps of this kind make at most a minor contribution to the reduced diversity near protein-coding exons. Nevertheless, these observations do not rule out the possibility that alternative modes of hitchhiking for which iHS has low power—such as recurrent hard or soft sweeps—might make a non-negligible contribution to patterns of variation near human protein-coding genes (see Discussion).

#### Genomic regions with extremely ancient most recent common ancestry

The previous section showed that genomic regions with reduced TMRCAs are often associated with purifying selection. To see whether the opposite signal was also of interest, we computed the posterior expected TMRCA in 10-kb blocks across the human genome and examined the regions displaying the oldest shared ancestry. Not surprisingly, four of the top twenty 10-kb blocks by TMRCA fall in the human leukocyte antigen (HLA) region on chromosome 6 (see Table 2). It has been known for decades that the HLA region exhibits extraordinary levels of genetic diversity, which is believed to be maintained by some type of balancing selection (overdominance or frequency-dependent selection) associated with the immunity-related functions of the HLA system [73]–[75]. The four HLA-related high-TMRCA blocks include three regions near *HLA-F* and one region between *HLA-A* and *HLA-J* (Supplementary Figure S12). All four high-TMRCA regions exhibit more than 12 polymorphisms per kilobase of unfiltered sequence, 8–10 times the expected neutral rate after normalizing for local mutation rates (as detailed in Table 2; see also Supplementary Figure S13). The estimated TMRCAs for these regions range from ∼340,000–380,000 generations, or ∼8.5–9.5 My (assuming 25-year generations).

**Table 2:** Top twenty 10 kb regions in the human genome by estimated TMRCA.

#	Chr^a^	Start	End	TMRCA^b^	Poly/kb^c^	Npoly^d^	CNV^e^	Comments
1	chr4	190590001	190600000	615775	16.6	32.8	√	Part of large intergenic region near telomere of long arm of chr 4 (see [76])
2	chr5	21560001	21570000	503311	16.2	5.1	√	Intron of *GUSBP1*
3	chr3	97930001	97940000	479803	16.4	5.3		Intergenic region in cluster of olfactory receptor genes
4	chr6	57270001	57280000	479504	13.7	28.0	√	Intron of *PRIM2*
5	chr2	223940001	223950000	449728	19.8	4.3		Intergenic region downstream of *KCNE4*
6	chr5	21550001	21560000	412679	14.2	4.4	√	Intron of *GUSBP1*
7	chr6	57220001	57230000	399887	16.2	12.8	√	Intron of *PRIM2*
8	chr6	29680001	29690000	380228	15.3	10.0		Intergenic region upstream of *HLA-F*
9	chr1	94220001	94230000	377017	8.0	4.2		Intron of *BCAR3*
10	chr8	123070001	123080000	375128	15.3	4.2		Intron of *BC052578*
11	chr11	55670001	55680000	374537	12.0	4.3		Intergenic region between *TRIM51* and *OR5W2*
12	chr6	29950001	29960000	371110	17.6	7.6		Intergenic region between *HLA-A* and *HLA-J*
13	chr17	64010001	64020000	367842	8.6	5.5		Intron of *CEP112*
14	chr6	29670001	29680000	365313	15.8	10.1		Intergenic region upstream of *HLA-F*
15	chr11	55690001	55700000	361088	11.5	4.1		Intergenic region between *OR5W2* and *OR5I1*
16	chr6	158680001	158690000	345382	10.4	4.8		Intergenic region upstream of *TULP4*
17	chr6	29720001	29730000	341797	12.4	8.0		Intergenic region between *HLA-F* and *HLA-G*
18	chr17	43790001	43800000	335647	11.2	5.0		Intron of *CRHR1*
19	chr6	8470001	8480000	325656	10.1	4.5		Intron of noncoding RNA *LOC100506207*
20	chr4	141920001	141930000	325570	12.1	3.2		Intron of *RNF150*

a Genomic coordinates in hg19 assembly. The genome was simply partitioned into nonoverlapping 10 kb intervals in hg19 coordinates.

b Posterior expected TMRCA in generations, averaged across unfiltered genomic positions in region.

c Number of polymorphisms in Complete Genomics dataset in region per kilobase of unfiltered sequence.

d Normalized polymorphism rate: number of polymorphisms per unfiltered kilobase divided first by the local mutation rate (as estimated from divergence to nonhuman primate outgroup genomes) then by the average of the same polymorphism/divergence ratio in designated neutral regions. The resulting value can be interpreted as a fold increase in the mutation-normalized polymorphism rate compared with the expectation under neutrality. The same measure was computed from the much larger 1000 Genomes Project Phase 1 data set, and was significantly elevated in these 20 high-TMRCA regions (Supplementary Figure S13).

e Possible copy number variant (CNV), based on Complete Genomics “hypervariable” or “invariant” labels (see Methods). Polymorphism rates in these regions may be over-estimated.

Among these high-TMRCA blocks were two additional regions that displayed extraordinary levels of mutation-rate-normalized nucleotide diversity. The first of these, in a gene desert near the telomere of the long arm of chromosome 4, exhibits the deepest expected TMRCA in the genome, at >600,000 generations (15 My), and has >30 times the neutral polymorphism rate (Table 2). The second region is the *PRIM2* gene on chromosome 6, which contributes the 4th and 7th highest TMRCA blocks in the genome, exhibiting polymorphism rates 28.0 and 12.8 times the neutral expectation, respectively. Both of these regions were identified as extreme outliers in a recent study of coincident SNPs in humans and chimpanzees, and it was argued that the *PRIM2* gene was a likely target of balancing selection [76]. On closer inspection, however, we found that both regions were flagged by Complete Genomics as having “hypervariable” or “invariant” read depth across individuals, suggesting that the elevated SNP rates in our data are likely artifacts of copy number variation (CNV) at loci unduplicated in the reference genome. (Leffler et al. recently reached a similar conclusion about *PRIM2* [77].) Despite that these flags were associated with only ∼5% of genomic positions, they indicated that five of our top six regions were likely CNVs (Table 2). Thus, for all subsequent analyses reported in this paper and for our publicly available browser tracks, we filtered out all regions labeled as invariant or hypervariable.

Once these extreme outliers were excluded, several loci of interest remained. In addition to the four *HLA* loci, these included (#5 in Table 2) an apparent *cis*-regulatory region downstream of the *KCNE4* gene, which encodes a potassium voltage-gated channel (Supplementary Figure S14); (#9) an intronic interval in *BCAR3*, a gene involved in the development of anti-estrogen resistance in breast cancer (Supplementary Figure S15); (#16) an apparent regulatory region upstream of *TULP4*, a tubby-like protein that may be involved in ubiquitination and proteasomal degradation with a possible association with cleft lip (Supplementary Figure S16); and (#18) an intronic region in *CRHR1*, which encodes a GPCR that binds corticotropin releasing hormones, has roles in in stress, reproduction, immunity, and obesity, and is associated with alcohol abuse, asthma, and depression. Notably, all of these are predominantly noncoding regions that include multiple ChIP-seq-supported transcription factor binding sites. The estimated TMRCAs of these regions range from 335,000–450,000 generations (8.4–11.3 My), suggesting genetic variation in these loci considerably predates the human/chimpanzee divergence.

#### Segregating haplotypes shared between humans and chimpanzees

To explore the connection between extreme TMRCAs and balancing selection further, we examined 125 loci recently identified as having segregating haplotypes that are shared between humans and chimpanzees [77]. These loci are expected to be enriched for ancient polymorphisms maintained by balancing selection, although some may reflect independent occurrences of the same mutation in both species. We compared these putative balancing selection loci with neutral sequences having the same length distribution (see Methods), and found that their *ARGweaver*-estimated TMRCAs were clearly shifted toward higher values, with a mean value nearly twice as large as that of the neutral sequences (Supplementary Figure S17). In addition, the putative balancing selection loci that do not contain polymorphisms in CpG dinucleotides—which are less likely to have experienced parallel mutations—had slightly higher TMRCAs than the group as a whole.

If these loci are sorted by their estimated TMRCAs, several loci that were highlighted by Leffler et al. [77] for having more than two pairs of shared SNPs in high LD appear near the top of the list (Table 3). For example, the haplotype between the *FREM3* and *GYPE* genes (#11 in Table 3; Supplementary Figure S18) contains shared SNPs in almost perfect LD with several expression quantitative trait loci (eQTLs) for *GYPE*, a close paralog of a gene (*GYPA*) that encodes a receptor for *Plasmodium falciparum* and may be under balancing selection. Another haplotype (#3) contains shared SNPs in significant LD with an eQTL for *MTRR*, a gene implicated in the regulation of folate metabolism, including one SNP that is also segregating in gorillas. In a third case (#18), the shared SNPs occur in a likely enhancer in an intron of *IGFBP7*, a gene that plays a role in innate immunity, among other functions. Another example is a locus near the *ST3GAL1* gene (#7) that contains only one pair of shared SNPs but was suggested by a phylogenetic analysis to have an ancient origin [77]. Notably, all of these shared haplotypes fall outside of coding regions and several show signs of regulatory activity based on functional genomic data [77]. Their expected TMRCAs range from roughly 150,000 to 250,000 generations, or 3.8–6.3 My. Thus, the *ARGweaver* estimates of age are reasonably consistent with the hypothesis that these hapolotypes predate the human/chimpanzee divergence (estimated at 3.7–6.6 Mya [57]), an observation that is especially notable given that our analysis does not make direct use of data from chimpanzees.

**Table 3:** Top twenty regions of shared human/chimpanzee haplotypes by estimated TMRCA.

#	Chr^a^	Start	End	TMRCA^b^	Poly/kb^c^	Npoly^d^	CNV^e^	Comments
1	chr7	47799979	47803415	307590	10.5	2.9		First exon/intron of *LINC00525*
2	chr4	56144164	56148467	256051	14.4	4.0		Upstream of *SRD5A3*
3	chr5	8022829	8024476	249553	9.3	2.0		Downstream of *MTRR*
4	chr3	143684547	143688535	235598	9.8	2.9		Upstream of *C3orf58*
5	chr9	99546087	99550934	233492	8.6	2.6		Upstream of *ZNF510*
6	chr18	58437379	58439410	228782	8.5	1.8		Distally upstream of *MC4R*
7	chr8	134404327	134405512	227555	16.4	3.7		Downstream of *ST3GAL1*
8	chr21	22045484	22048252	215718	12.2	2.5		Downstream of *LINC00320*
9	chr7	45252745	45257527	201522	13.5	4.3		Downstream of *RAMP3*
10	chr2	241121578	241124345	200321	16.1	3.0	√	Upstream of *OTOS*
11	chr4	144654907	144662554	182348	11.9	2.5		Upstream of *FREM3*
12	chr3	36203964	36205036	173655	15.5	2.9		Upstream of *STAC*
13	chr2	101276944	101278537	173448	14.0	2.9		Downstream of *PDCL3*
14	chr1	157716093	157718074	170583	10.1	2.4		Exon and introns of *FCRL2*
15	chr14	22320920	22323473	159251	13.8	2.4		Intron of *TCRA*
16	chr14	88803535	88805909	155431	8.9	2.2		Upstream of *KCNK10*
17	chr20	5337103	5340864	149816	11.1	2.8		Upstream of *PROKR2*
18	chr4	57919549	57920587	146684	17.5	4.9		Intron of *IGFBP7*
19	chr14	86147042	86149069	143608	10.1	2.1		Downstream of *FLRT2*
20	chr11	81489342	81492793	143222	10.2	1.8		Downstream of *BC041900*

a Genomic coordinates in hg19 assembly.

b Posterior expected TMRCA in generations, averaged across unfiltered genomic positions in region.

c Number of polymorphisms in Complete Genomics dataset in region per kilobase of unfiltered sequence.

d Normalized polymorphism rate: number of polymorphisms per unfiltered kilobase divided first by the local mutation rate (as estimated from divergence to nonhuman primate outgroup genomes) then by the average of the same polymorphism/divergence ratio in designated neutral regions (see Methods). The resulting value can be interpreted as a fold increase in the mutation-normalized polymorphism rate compared with the expectation under neutrality.

e Possible copy number variant (CNV), based on Complete Genomics “hypervariable” or “invariant” labels (see Methods). Polymorphism rates in these regions may be inflated. Few of these regions were identified in the Leffler et al. data set, probably because the authors were careful to filter out duplicated regions from their analysis [77].

By contrast, the loci near the bottom of the list (with the shortest TMRCAs) appear to be much less convincing. For example, the bottom 20 have expected ages of only 25,000–50,000 generations (0.65–1.3 My), suggesting that they actually post-date the human/chimpanzee divergence by millions of years. In addition, many of these regions appear hundreds of kilobases from the nearest gene, and they typically do not overlap regions with strong functional or comparative genomic evidence of regulatory potential. Indeed, if our ARG-based estimates of the TMRCA are interpreted literally, a majority of the 125 segregating haplotypes may post-date the human/chimpanzee divergence, which current estimates would place at ≥150,000 generations ago (see Supplementary Figure S17). This observation is in general agreement with rough calculations by Leffler et al. suggesting that the false discovery rate for ancient balancing selection in this set could be as high as 75% [77]. Thus, it appears that our ARG-based methods may be useful in distinguishing true ancestral polymorphisms from shared haplotypes that occur by chance due to homoplasy.

#### Natural selection and allele age

**Figure 6:**
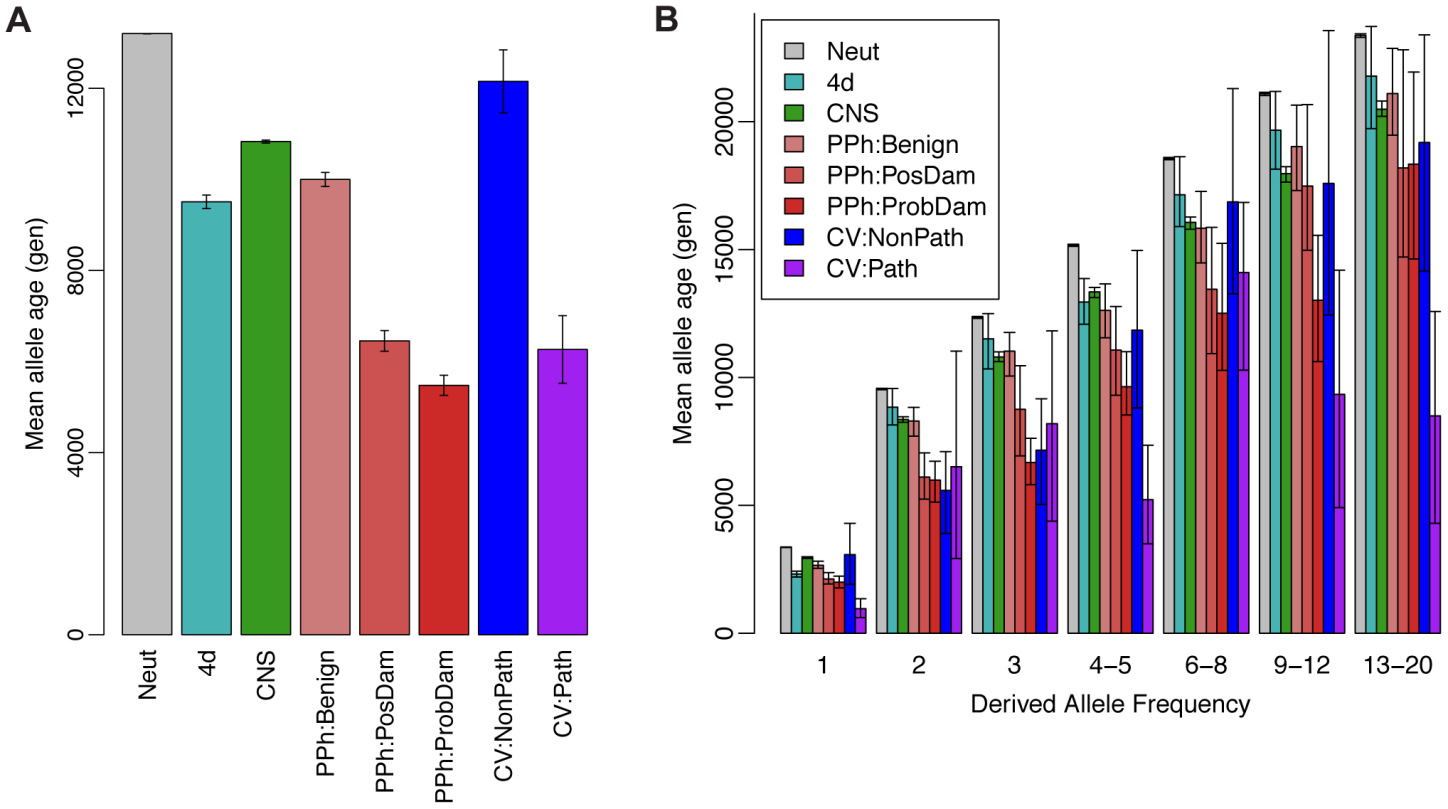
Mean allele age as a function of annotation class and derived allele frequency. (A) Estimated age of derived allele in generations, averaged across polymorphic sites of various annotation classes. Estimates were derived from ARGs sampled by *ARGweaver* based on the Complete Genomics data set (see Methods). Error bars represent one standard deviation above and below the mean. Neut = putatively neutral sites; 4d = fourfold degenerate sites in coding regions; CNS = conserved noncoding sequences identified by phastCons; PPh:{Benign,PosDam,ProbDam} = missense mutations identified by PolyPhen-2 as “benign”, “possibly damaging”, or “probably damaging”, respectively; CV:{NonPath,Path} = mutations in “nonpathogenic” (categories 1–3) or “pathogenic” (categories 4 & 5) classes in the ClinVar database, respectively. (B) Similar plot with categories further divided by derived allele frequencies (DAF) in numbers of chromosomes out of 108. Error bars represent 95% confidence intervals, as assessed by bootstrapping. In categories that combine multiple frequencies (e.g., 4–5, 6–8), a subsampling strategy was used to ensure that the relative contributions of the different frequencies matched those of the Neut class. Estimates for DAF>20 were excluded due to sparse data. Notice that ages generally increase with DAF, as expected (see Supplementary Figure S7), but at a considerably reduced rate in categories under strong selection.

Next we examined the ARG-based expected ages of derived alleles at polymorphic sites in various annotation classes. Classical theory predicts that both deleterious and advantageous alleles will not only have skewed population frequencies but will also tend to be younger than neutral alleles at the same frequency, because directional selection will tend to accelerate a new mutation's path to fixation or loss [78]. This idea has recently been used to characterize selection in the human genome based on a haplotype-based summary statistic that serves as a proxy for allele age [79]. We computed ARG-based estimates of allele age in putatively neutral regions (Neut), fourfold degenerate sites in coding regions (4d), conserved noncoding sequences (CNS), missense coding mutations predicted by PolyPhen-2 to be “benign” (PPh:Benign), “possibly damaging” (PPh:PosDam), or “probably damaging” (PPh:ProbDam), and coding or noncoding mutations classified by the ClinVar database (http://www.ncbi.nlm.nih.gov/clinvar) as “nonpathogenic” (categories 1–3; CV:NonPath) or “pathogenic” (categories 4 & 5; CV:Path) based on direct supporting evidence of phenotypic effects. We found, indeed, that the Neut mutations were significantly older, on average, than all other classes (Figure 6A). In addition, among the missense coding mutations, PPh:Benign mutations were the oldest, PPh:PosDam were significantly younger, and PPh:ProbDam mutations were the youngest. Similarly, mutations in the CV:NonPath class were significantly older than those in the CV:Path class. Interestingly, the 4d mutations showed substantially lower average ages (by >30%) than the Neut mutations. We attribute this reduction primarily to the effects of selection from linked sites (see [60]), although direct selection from mRNA secondary structure and exonic regulatory elements may also contribute to it.

In part, these differences in age simply reflect differences in the site frequency spectrum (SFS) across classes of mutations. For example, missense mutations are well known to be enriched for low-frequency derived alleles, which will tend to be younger, on average, than higher-frequency derived alleles. To account for the influence of allele frequency, we further grouped the sites in each annotation class by derived allele frequency and compared the average allele ages within each group (Figure 6B). As expected, the estimated ages increase with the derived allele frequency across all annotation classes. In addition, within each class we continue to observe approximately the expected rank-order in allele ages, with Neutral mutations being the oldest, 4d, PPh:Benign, CNS, and CV:NonPath mutations coming next, followed by PPh:PosDam, PPh:ProbDam, and CV:Path mutations. This analysis demonstrates that *ARGweaver* is able to obtain information about natural selection from allele ages beyond what can be obtained from the SFS alone.

Another way of viewing these results is to consider the reduction in allele age relative to the neutral expectation within each frequency group, across annotation classes (Supplementary Figure S19). As expected, these reductions are larger at higher allele frequencies, where sojourn times will tend to be longer. However, from this representation it is also clear that the reductions in age increase with frequency much more rapidly for the mutations under strong, direct selection than for the mutations at which selection from linked sites is expected to dominate. For example, at very low derived allele frequencies (singletons), the reduction in age of 4d mutations is roughly equal to that at PPh:PosDam mutations, whereas at higher derived allele frequencies the damaging mutations exhibit reductions in age 2–3 times larger. The reason for this observation is probably that the reduction in age for the nearly neutral sites is largely a consequence of reductions in the local effective population size due to selection at linked sites, while the reductions at sites under direct selection are driven by the influence of selection on sojourn times (see Supplementary Figure S19 for a detailed discussion). Consistent with this interpretation, CNS mutations show less reduction in age than 4d and PPh:Benign mutations at low frequencies, and more reduction at high frequencies, suggesting that CNS mutations are influenced less by selection at linked sites and more by direct selection.

## Discussion

Several decades have passed since investigators first worked out the general statistical characteristics of population samples of genetic markers in the presence of recombination [21], [80]–[83]. Nevertheless, solutions to the problem of explicitly characterizing this structure in the general case of multiple markers and multiple sequences—that is, of making direct inferences about the ancestral recombination graph (ARG) [19], [20]—have been elusive. Recent investigations have led to important progress on this problem based on the Sequentially Markov Coalescent (SMC) [17], [37]–[42], but existing methods are still either restricted to small numbers of sequences or require severe approximations. In this paper, we introduce a method that is faithful to the SMC yet has much better scaling properties than previous methods. These properties depend on a novel “threading” operation that can be performed in a highly efficient manner using hidden Markov modeling techniques. Inference does require the use of Markov chain Monte Carlo (MCMC) sampling, which has certain costs, but we have shown that the sampler mixes fairly well and converges rapidly, particularly if the threading operation is generalized from single sequences to subtrees. Our methods allow explicit statistical inference of ARGs on the scale of complete mammalian genomes for the first time. Furthermore, the sampling of ARGs from their posterior distribution has the important advantage of allowing estimation of any ARG-derived quantity, such as times to most recent common ancestry, allele ages, or regions of identity by descent.

Despite our different starting point, our methods are similar in several respects to the conditional sampling distribution (CSD)-based methods of Song and colleagues [49]–[52]. Both approaches consider a conditional distribution for the 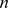
th sequence given the previous 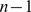 sequences, and in both cases a discretized SMC is exploited for efficiency of inference. However, the CSD-based methods consider the marginal distribution of the 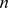
th sequence only given the other 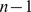 sequences and never explicitly reconstruct an ARG, while ours considers the joint distribution of an ARG of size 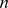 and the 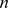
th sequence, given an ARG of size 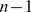 and the previous 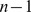 sequences. In a sense, we have employed a “data augmentation” strategy by explicitly representing full ARGs in our inference procedure. The main cost of this strategy is that it requires Markov chain Monte Carlo methods for inference, rather than allowing direct likelihood calculations and maximum-likelihood parameter estimation. The main benefit is that it provides an approximate posterior distribution over complete ARGs and many derived quantities, including times to most recent common ancestry, allele ages, and distributions of coalescence times. By contrast, the CSD-based methods provide information about only those properties of the ARG that are directly described by the model parameters. We view these two approaches as complementary and expect that they will have somewhat different strengths and weaknesses, depending on the application in question.

Our explicit characterization of genealogies can be exploited to characterize the influence of natural selection across the genome, as shown in our analysis of the Complete Genomics data set. In particular, we see clear evidence of an enrichment for ancient TMRCAs in regions of known and predicted balancing selection, reduced TMRCAs near protein-coding genes and selective sweeps, and reduced allele ages in sites experiencing both direct selection and selection at closely linked sites. Interestingly, the genealogical view appears to have the potential to shed light on the difficult problem of distinguishing between background selection and hitchhiking. Our initial attempt at addressing this problem relies on a genealogy-based summary statistics, the relative TMRCA halflife (RTH), that does appear to distinguish effectively between protein-coding genes and partial selective sweeps identified by iHS. However, more work will be needed to determine how well this approach generalizes to other types of hitchhiking (e.g., complete sweeps, soft sweeps, recurrent sweeps) and whether additional genealogical information can be used to characterize the mode of selection more precisely. Additional work is also needed to determine whether our ARG-based allele-age estimator—which is highly informative in bulk statistical comparisons but has high variance at individual sites—can be used to improve functional and evolutionary characterizations of particular genomic loci. A related challenge is to see whether our genome-wide ARG samples can be used to improve methods for association/LD mapping (see [34], [84]–[88]).

In addition to natural selection, our methods for ARG inference have the potential to shed light on historical demographic processes, an area of particular interest in the recent literature [16], [17], [51], [52], [89]. To explore the usefulness of *ARGweaver* in demography inference, we attempted to infer a population phylogeny with admixture edges for the 11 human populations represented in the Complete Genomics data set, based on the genealogies sampled under our naive (panmictic) prior distribution. We extracted 2,304 widely spaced loci from our inferred ARGs, obtained a consensus tree at each locus, and reduced this tree to a subtree with one randomly selected chromosome for each of the 11 populations (see Text S1 for details). We then analyzed these 11-leaf trees with the PhyloNet program [90], [91], which finds a population tree that minimizes the number of “deep coalescences” required for reconciliation with a given set of local trees, allowing for both incomplete lineage sorting and hybridization (admixture) events between groups. PhyloNet recovered the expected phylogeny for these populations in the absence of hybridization and generally detected complex patterns of gene flow where they are believed to have occurred, but it had difficulty reconstructing the precise relatinonships among source and admixed populations (Supplementary Figure S20). These experiments suggested that the posterior distribution of ARGs does appear to contain useful information about population structure even when a noninformative prior distribution is used, but that additional work will be needed to fully exploit the use of ARG inference in demographic analysis.

An alternative strategy would be to extend our methods to incorporate a full phylogenetic demographic model, such as the one used by G-PhoCS [92], thereby generalizing this fully Bayesian method to a setting in which recombination is allowed and complete genome sequences are considered. Importantly, the use of the complete ARG would allow information about demographic history from both patterns of mutation and patterns of linkage disequilibrium to be naturally integrated (see [92]). However, as with CSD-based methods [51], [52], an extension to a full, parametric multi-population model for application on a genome-wide scale would be technically challenging. In our case, it would require the ability to sample “threadings” consistent with the constraints of a population model (e.g., with no coalescent events between genetically isolated populations) and exploration of a full collection of population parameters, which would likely lead to slow convergence and long running times. Nevertheless, a version of this joint inference strategy may be feasible with appropriate heuristics and approximations. Our methods may also be useful for a wide variety of related applications, including local ancestry inference [47], [93], [94], haplotype phasing/genotype imputation [46], [48], [95], [96], and recombination rate estimation [22], [97].

Our initial implementation of *ARGweaver* relies on several simplying assumptions that appear to have minimal impact on performance with (real or simulated) human sequence data, but may produce limitations in other settings. Following Li and Durbin [42], we compute probabilities of recombination between discrete genomic positions under the assumptions of the continuous-space SMC [37]. When recombination rates are low, the discrete and continuous models are nearly identical, but the differences between them can become significant when recombination rates are higher [98]. Similarly, our assumption of at most one recombination event per site and our use of the SMC rather than the improved 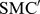 [38] may lead to biases in cases of higher recombination rates, larger numbers of sequences, or more divergent sequences. In addition, our heuristic approach of accommodating zero-length branches by randomly sampling among “active” branches for coalescence and recombination events (see Methods) may lead to biases when the discretization scheme is coarse relative to evolutionary events of interest. Finally, we currently assume haploid genome sequences as input, which, in most cases of current interest, requires computational phasing as a pre-processing step. Phasing errors may lead to over-estimation of recombination and mutation rates and associated biases, because the sampler will tend to compensate for them with additional recombination and/or mutation events. In principle, most of these limitations can be addressed within our framework. For example, it should be fairly straightforward to extend *ARGweaver* to use the SMC' and Hobolth and Jensen's finite-loci transition density. In addition, we believe it is possible to enable the program to work directly with unphased data and integrate over all possible phasings (see, e.g., [92], [99]).

The ability to perform explicit ARG inference on the scale of complete genomes opens up a wide range of possible applications, but the long running times required for these analyses and the unwieldy data structures they produce (numerous samples of ARGs) are potential barriers to practical usefulness. In our initial work, we have attempted to address this problem by precomputing ARGs for a highly informative public data set and releasing both our complete ARGs and various summary statistics as browser tracks for use by other groups. We have also developed a simple web interface that allows users to retrieve local trees and several useful summary statistics for specified genomic intervals, populations, and individuals (http://compgen.bscb.cornell.edu/ARGweaver/CG_results). In future work, it may be possible to improve data access by providing more sophisticated tools for data retrieval and visualization. For example, sampled ARGs could be stored in a database in a manner that allowed researchers to efficiently extract features such as regions of IBD or recombination maps for designated subsets of samples. A related possibility would be to support on-the-fly threading of user-specified query sequences into precomputed ARGs. This operation would be analogous to local ancestry inference [47], [93], [94], but would reveal not only the population sources of query sequence segments, but also additional information about recombination events, coalescence times, approximate mutation ages, and other features. The same operation could be used to allow our sampling methods to scale to thousands of genomes: one could infer ARGs for, say, 100 genomes, then simply thread in hundreds more, without full MCMC sampling. In general, we believe that posterior samples of ARGs will be a rich resource for genetic analysis, but additional work is needed on data storage and query interfaces for these samples to become practically useful to large numbers of genomic researchers.

## Methods

### Discretized Sequentially Markov Coalescent

#### Discretization scheme and notation

The Discretized Sequentially Markov Coalescent (DSMC) assumes that all coalescence and recombination events occur at 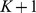 discrete time points, 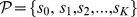, with 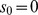 (the present time) and 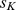 equal to a user-specified maximum value. These time points are defined in units of generations before the present time. We evenly distribute these time points on a logarithmic scale, so that the discretization scheme has finer resolution near the leaves of the ARG, where more events are expected to occur. Specifically, we define 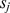 (for 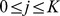) to be 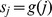, where
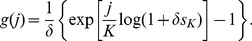
(4)Here, 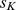 is the maximum time and 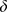 is a tuning parameter that, when increased, causes the time points to become more densely clustered near the leaves of the ARG. Notice that 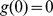 and 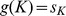. In this work, we have assumed 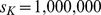 generations and 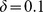. We denote the length of time interval 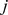 as 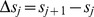. The DSMC process is defined such that it approaches the continuous SMC as a limit as 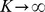 and each 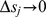, with 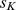 sufficiently large that the probability of a coalescence event older than 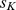 is close to zero.

It is useful to specify “midpoints” between time points (on a log scale), to facilitate rounding of continuous-valued times to the nearest discrete time point. We define the midpoint between times 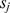 and 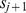 (for 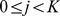) as 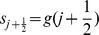. We can alternatively refer to the midpoint between times 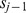 and 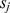 as 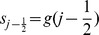 (for 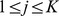), noting that 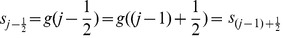. Coalescence events that occur between 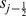 and 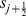 are “rounded” to time point 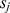. We found that it was less critical to round recombination events to the nearest time point, so they are simply rounded to the next most recent time point (see below). We denote the lengths of the half intervals between 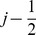 and 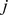, and between 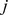 and 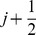, as 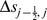 and 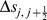, respectively.

Because all coalescence events must occur at the designated time points, the collection of branches is fixed for each interval 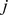 between time points 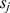 and 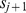. Given a local tree 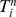 that is consistent with the DSMC, we denote the set of branches in time interval 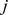 as 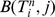. The size of this set, 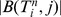, is of particular interest, and is abbreviated 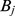 (with 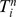 clear from context). In addition, it is often of interest to consider the branch sets for a tree 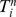 from which a branch 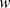 has been removed. We denote such a tree by 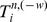 and abbreviate the number of branches in interval 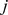 as 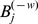 (again, with 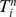 clear from context).

One consequence of discretizing time is that the DSMC will tend to generate ARGs that contain many branches of length zero (corresponding to polytomies in the local trees), which will have zero probability of recombination, coalesce, or mutation events. In effect, the rounding procedure will tend to shrink short branches to zero, which may lead to distortions in data generation and inference. We address this problem heuristically, by defining the DSMC to first sample the times of recombination and coalescence events, and then randomly select a branch from all of those “active” at the sampled time point. We define the set of active branches at a time point 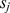, for a local tree 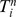, to be those branches in 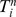 that start, end, or pass through 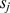. This set is denoted 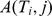 and its size is abbreviated as 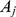. As above, we use 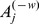 to indicate the active branches at 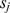 excluding branch 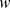. Simulations indicate that this heuristic solution to the problem of zero-length branches works fairly well in practice (see Figure S1).

#### Recombination process

As in the standard SMC, recombinations are assumed to occur according to a Poisson process with rate 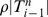, where 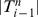 is the total branch length of local tree 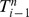 and 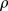 is the average number of recombinations/generation/site. Once a recombination occurs, the ordinary SMC process places the recombination uniformly along the branches of 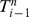. The analogous operation of sampling a recombination branch and time point, 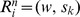, in the DSMC is accomplished by first sampling a time point 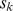 in proportion to the total branch length present during time interval 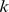, then randomly selecting one of the 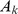 branches active at that time point. Consistent with the assumptions of the SMC, the recombination point cannot occur above the time point associated with the root 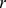 of tree 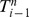, which we denote 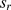. Thus, the sampling distribution for a recombination point 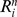 on a local tree 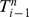 is given by,
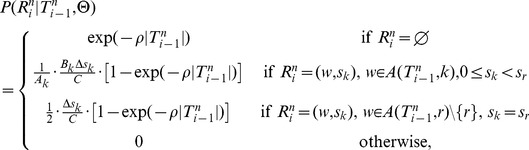
(5)where 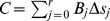 is a constant that explicitly normalizes the distribution over time points 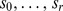. The special case for the time point at the root of the tree, 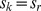, is required because the SMC does not allow recombinations to occur beyond this point, so the effective number of active branches is only two at this time point, despite that 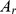 will have a value of three. The number of branches in the interval above the root, 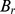, is necessarily one, so this term can be omitted in this case.

This sampling distribution effectively rounds the times of recombination events downward to the next most recent time point. However, a strict policy of downward rounding, together with a prohibition again recombination events above the root node, would make it impossible to sample recombination events at time point 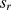, which turns out to have undesirable effects in inference (it makes some trees unreachable by the threading operation). Therefore, when sampling time points, we use the heuristic approach of imagining that recombinations can also occur in the time interval immediately above the root and assigning these events to the time point 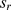. This has the effect of redistributing some of the probability mass from later time points to the root, without altering the overall rate at which recombinations occur (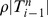). For this reason, the normalizing constant 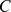 differs slightly from the total branch length 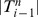; in particular, 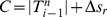. It would be slightly more elegant to allow upward as well as downward rounding of times for recombinations, as we do with coalescence events (see below), but as long as the time discretization is not too coarse these differences are of minor importance, and the approach we have used seems to be adequate.

#### Re-coalescence process

Once a recombination point 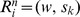 is sampled, the selected branch 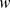 is removed from time points 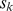 and older, and allowed to re-coalesce to the remainder of the tree, in a manner analogous to the SMC. Because we explicitly prohibit multiple recombinations between adjacent positions, the local tree 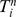 must be reachable from 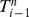 by a single “subtree pruning and regrafting” (SPR) operation corresponding to the recombination, i.e., an operation that cuts a branch of the tree at the recombination point and re-attaches it (and any descendant nodes) to the remainder of the tree. Thus, we can write,
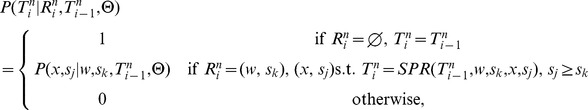
(6)where 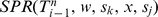 is a function that returns the new tree produced by an SPR operation on 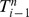 that cuts branch 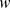 at time 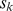 and re-attaches it to branch 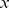 at time 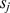, and 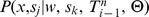 is a joint conditional distribution over re-coalescence branches and time points.

The main challenge is therefore to define the discrete re-coalescence distribution, 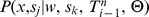, for 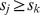 (as required by the SMC). There are two distinct cases to consider: 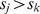 and 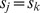. When 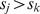, the unattached branch 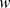 must first fail to re-coalesce during the interval between 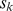 and 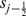, and then must re-coalesce between 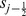 and 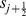 (because all such re-coalescence events will be rounded to 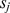). By contrast, when 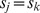, the branch 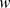 must simply re-coalesce between 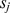 (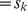) and 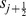 (because the re-coalescence time is strictly bounded by the recombination time).

In all cases, the instantaneous rate of re-coalescence in each interval 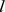 (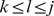) is given by 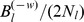, in the standard manner for the coalescent. (Note that we use 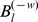 rather than 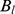, because we are concerned with the coalescence rate to the remainder of the tree, excluding branch 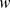. We also assume a diploid species throughout, so the total number of chromosomes per locus is 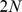.) The probability that a lineage starting at a time 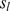 coalesces before 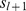 is given by the cumulative distribution function for exponentially distributed waiting times,
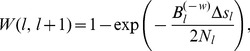
(7)and the probability of coalescence during a sequences of intervals, 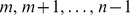 is given by,
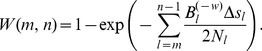
(8)Similarly, the probabilities of coalescence during the half intervals before and after time point 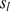 are given, respectively, by,
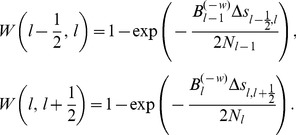
(9)Thus, the distribution of re-coalescence times for the case of 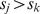 is given by,
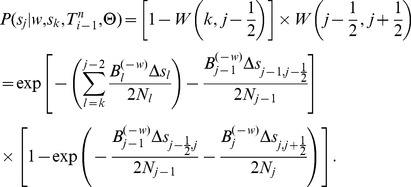
(10)The probability of re-coalescence for the case of 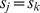 is simply,
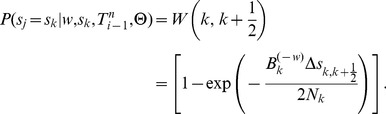
(11)Finally, the requirement for re-coalescence by the maximum time, 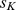, is enforced by explicitly normalizing the distribution:
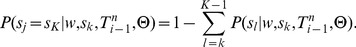
(12)


Once the coalescence time point 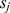 is chosen, a lineage 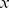 is uniformly chosen from the 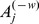 active lineages in 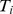 at that time point, similar to the process for recombination events. Thus, 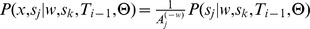, and equation 6 can be rewritten as,
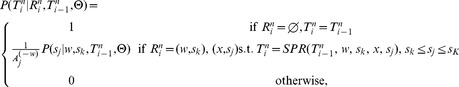
(13)where 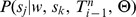 is given by equations 10–12.

#### Initial local tree

The DSMC begins by generating an initial local tree, 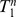, using a discretized version of the coalescent process. This process can be decomposed into two steps: (1) the generation of a sequence of branch counts, 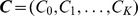 for time points 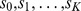, and (2) sampling of a topology 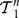 consistent with these branch counts. The probability of an observed initial tree 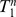 can therefore be calculated as,
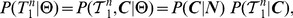
(14)where 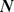 is a vector of effective population sizes, 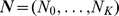. The branch count for time 0 is constrained to be equal to the number of samples, 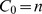, and the branch count for time 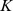 is required to be one, 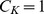 (see below).

Since the coalescent process is Markovian in time, the distribution for the vector of branch counts can be factored by time intervals,
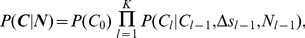
(15)with degenerate first and last terms, 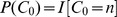 and 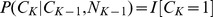.

The conditional distributions of the form 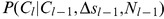, for 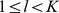, have been derived previously as [100],
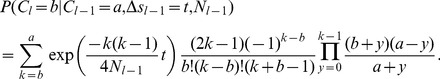
(16)


### Hidden Markov Model

#### Hidden Markov model for full threading problem

As noted in the Results section, the complete data likelihood function under the DSMC is given by equation 2. If the full ARG 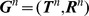 is regarded as a latent variable, this equation defines a hidden Markov model with a state space given by all possible pairs 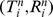, transition probabilities given by expressions of the form 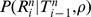 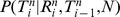 and emission probabilities given by 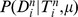 (see Figure 3A). The transition probabilities can be computed using equations 5 and 13, and the emission probabilities can be computed using Felsenstein's pruning algorithm. This model can be viewed as an instance of the “phylo-HMMs” that have been widely used in comparative genomics [101]. As discussed in the Results section, however, unless the number of sequences 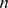 is very small, the state space of this HMM will be too large to allow it to be used directly for inference.

Instead, we constrain the inference problem by fixing the ARG for the first 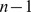 sequences, 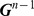, and sampling from the conditional distribution 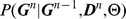. Using the notation 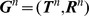 and 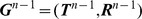, we define 
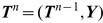
, where 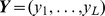 is a vector of coalescence points such that 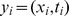 indicates a coalescence of the 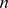
th sequence to branch 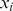 and time point 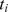 of local tree 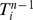, and 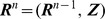, where 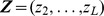 is a vector of recombination points such that 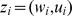 indicates a recombination at branch 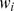 and time point 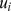 of local tree 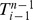 between positions 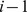 and 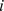. (Note that 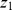 is undefined.) Thus, we can sample from the desired conditional distribution 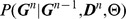 by sampling from 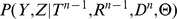. We refer to a sample 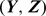 from this distribution as a *threading* of the 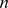
th sequence through the ARG (see Figure 3B). For now, we will consider a complete threading 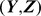, but in later sections we will describe our two-step process for sampling, first, the coalescent threading 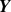, and second, the recombination threading 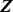 given 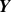.

Note that the restriction to one recombination event per position implies that 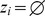 wherever 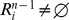, and that 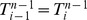 wherever 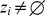. This restriction is not strictly required but it simplifies the description of new recombination events 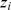, and in the setting of interest here it comes with little cost (see Discussion).

It turns out to be more convenient to work with the joint distribution 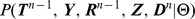 (the complete data likelihood) than with the conditional distribution 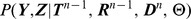. However, to emphasize that the variables 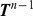 and 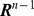 are held fixed (“clamped”) at pre-specified values throughout the threading operation, we denote them as 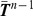 and 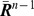, and refer to the distribution of interest as 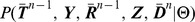. (Notice that the data 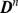 are also clamped, as usual for HMMs.) When 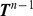, 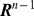, and 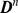 are clamped,
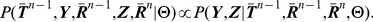
(17)Thus, samples of 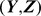 drawn in proportion to the unnormalized density 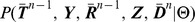 will be valid samples from the desired conditional distribution. In general, any clamped joint density function, 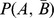, can be viewed as an unnormalized version of a corresponding conditional density function, 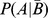, but sometimes the joint density is more convenient to manipulate.

We can now write the density function for the (unnormalized) sampling distribution for a threading 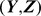 as,
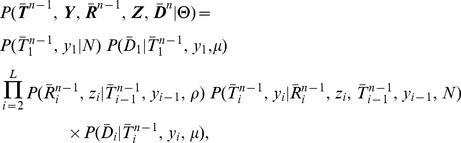
(18)where all terms are computable using previously described expressions, as for equation 2.

Notice that this threading HMM has the same conditional independence structure as the HMM for the full DSMC (equation 2, Figure 3), but its state space is now defined by sets of possible 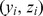 pairs rather than the set of possible 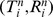 pairs, making it far more tractable for inference.

#### Reduced model for coalescent threading

The state space can be reduced further by proceeding in two steps. First, we sample a *coalescent threading* 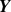 from the marginal distribution 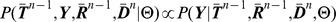. Then we sample a *recombination threading*, 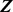, from the conditional distribution 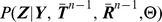. Notice that the data need not be considered when sampling the recombination threading, because 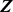 is conditionally independent of 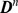 given 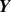, 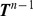, and 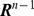.

The marginal distribution 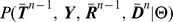 can be computed efficiently by changing the order of products and sums in the usual way for HMMs:
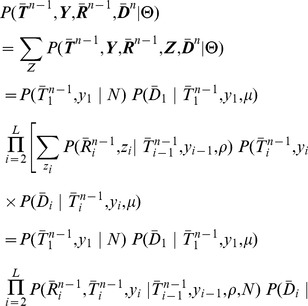
(19)This equation defines an HMM with a state space given by the possible values of 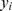 only, the size of which is bounded by 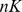, where 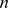 is the number of sequences and 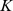 is the number of time intervals (see Figure 3C).

While this model has the conditional independence structure of a standard HMM, the state space is heterogeneous along the sequence, because the set of possible coalescent points at each position 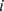 depends on the local tree, 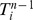. (The full threading HMM described above also has this property.) If we denote the state space at position 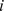 as 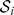, the transition probabilities between states in position 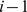 and states in position 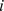 are defined by a 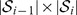 transition matrix 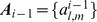 where 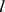 and 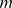 index the states of 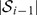 and 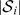, respectively, and 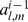 can be computed as,
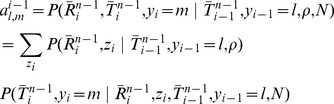
(20)using equations 5 and 13. The emission probability for alignment column 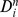 in state 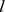 in 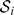 is denoted 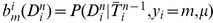 and can be computed using Felsenstein's pruning algorithm, as in all cases above. The initial state probabilities for the HMM are given by 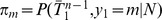 for 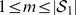 and can be computed using equations 14–16.

Notice that, unlike with a standard, locally normalized HMM, it is not true in this model that 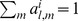. Furthermore, for two positions 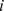 and 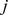, it is not true in general that 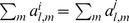, because of differences across positions in the local trees 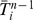 and recombination points 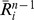. Similarly, it is not true that 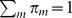. Thus, this model is not only globally unnormalized, but it also has a heterogeneous local normalization structure across positions. Importantly, this heterogeneity stems directly from differences in the 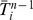 and 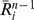 and is inherent in the threading problem—that is, it is not possible to express the desired conditional distribution, 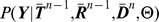, directly in terms of a locally normalized hidden Markov model (one in which all transition probabilities and all emission probabilities sum to one at each position in the sequence). For this reason, we find it most convenient to work with the unnormalized clamped joint distribution.

#### Stochastic traceback

Despite the unusual features of the HMM described in the previous section, it still permits the use of standard dynamic programming algorithms to integrate over all coalescent threadings 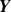 (the forward or backward algorithms), obtain a most likely threading 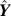 (the Viterbi algorithm), compute marginal posterior distributions for each 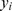 (forward-backward algorithm), and sample threadings in proportion to their conditional probability [102], [103]. These algorithms depend only on the linear conditional independence structure of the model (and, equivalently, on its factorization into local transition and emission probabilities) and on the use of nonnegative potential functions, both properties that are maintained in this model.

We are primarily interested in a dynamic programming algorithm for sampling from the posterior distribution over HMM paths that is sometimes referred to as the *stochastic traceback* algorithm [103]–[105]. In our case, each application of this algorithm is guaranteed to sample a coalescent threading 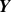 in proportion to the density 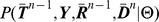, and equivalently, in proportion to the desired conditional distribution.

The stochastic traceback algorithm consists of a deterministic forward pass and a stochastic backward pass. The forward pass is identical to the forward algorithm. In our notation, the algorithm recursively fills out a matrix 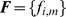, 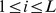, 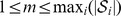. Each 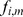 represents the probability of a prefix of the data joint with a constraint on the state path at position 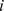. Here, 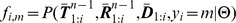, where the notation 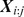 indicates the subsequence 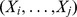. After an initialization of 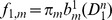, for 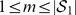, the algorithm proceeds iteratively for 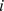 from 2 to 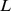 and sets each value 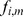 (for 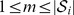) equal to,
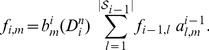
(21)Note that the heterogeneity of the state space along the sequence implies that portions of the matrix are left undefined.

In the backward pass, the algorithm samples a sequence 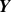 one element at a time, starting with 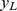 and working backward to 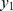. First, 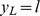 is simply sampled in proportion to 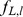. Then, for 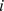 from 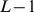 down to 1, each 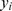 is sampled conditional on 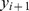 in proportion to,
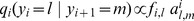
(22)The limiting step of the algorithm is the forward pass, which in general requires 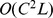 time, where 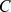 is the size of the state space, 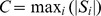. However, in our case the structure of the 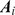 matrices can be exploited to reduce the running time to 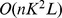 (see Text S1).

It can be shown by induction on suffixes of 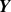 that this procedure will correctly sample from the target distribution, 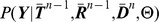. Briefly, in the base case, the suffix 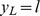 is by construction sampled from the density 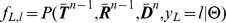, which is proportional to the desired conditional distribution, 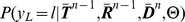. For the inductive case, assume 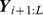 has been sampled from 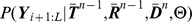. The procedure of sampling 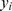 from 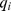 given 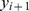 is equivalent to sampling from,
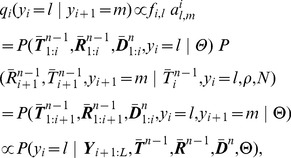
(23)where the last step is possible because 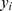 is conditionally independent of 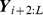, 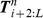, 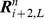, and 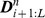 given 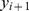. Thus, the algorithm will correctly sample from 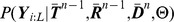 for all 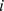 such that 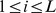.

#### Sampling a recombination threading

The final step in the threading operation is to sample a recombination threading 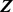 conditional on a coalescent threading 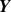 and the clamped parameters. This step is greatly simplified by the fact that the individual 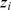 values are conditionally independent of one another given the 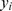 variables and the clamped 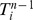 and 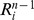 variables (see Figure 3B). Consequently, each 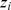 can be sampled separately from the distribution,
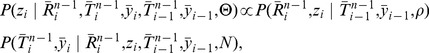
(24)where the 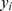 variables are now clamped along with the 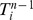 and 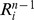 variables. Notice that the distribution on the RHS is the same one considered in equations 19 & 20. The normalizing constant for this distribution, for clamped values 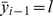 and 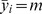, is given by the transition probability 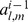.

Notice that this distribution is implicitly degenerate in the case in which 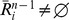, owing to the limitation of at most one recombination event per position. In particular, if 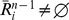, then 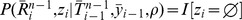, hence 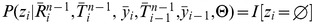. At the same time, notice that, if 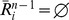, a new recombination is still possible (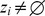) even if 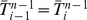 and 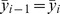, because a branch could be broken by a recombination event but then re-coalesce at precisely its original position in the local tree.

When 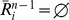, the efficiency of sampling from this distribution can be improved by noting that most possible 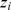 values still have zero probability. Let 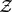 represent the set of 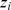 values having nonzero probability for given values of 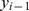, 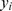, and 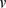, where 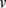 denotes the branch being threaded. There are two cases to consider, a main case and a special case. We will denote the corresponding subsets of 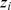 values 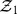 and 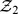, with 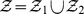. Recall that 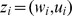 and 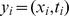, where 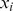 and 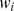 are branches in 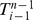 and 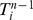, respectively, and 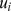 and 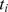 are time points from the set 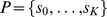. In the main case, the recombination occurs on the new branch 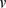. Here, the recombination time 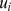 must be at least as recent as both the old and new re-coalescence times, 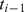 and 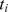. Thus, 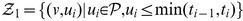. Notice that 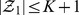.

The special case occurs when the recombination occurs not on the new branch, 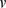, but instead on 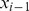, the branch to which 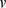 re-coalesces at position 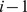. A recombination on branch 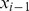, below the point at which 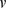 joins it, followed by a re-coalescence of 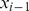 to 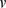 (meaning that 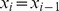) will produce a signature exactly like the symmetric case of a recombination on 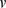 followed by a re-coalescence to 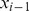 (Supplementary Figure S21), so this scenario must also be considered. This case can only occur when 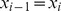 and in the interval of time between the start of branch 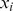 and 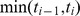. Recombinations on other branches need not be considered, because the existence of such a recombination would imply that 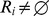, contrary to our assumption. Hence,
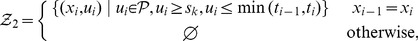
(25)where 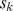 is the time point of the child node of branch 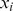. As with 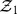, 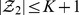.

By enumerating the elements of 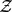, it is possible to sample each 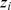 in 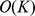 time. The same approach can be used to enable calculation of the 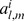 values (equation 20) in 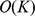 time.

### Data Preparation

#### Simulated data

Except where noted otherwise, simulations were performed under the full coalescent-with-recombination model [21]. After generation of local trees, sequence alignments were generated using a finite-sites Jukes-Cantor model [53]. All simulations were performed using custom computer programs. Our standard simulation scheme involved the generation of of twenty 1-Mb sequences, assuming an effective population size of *N* = 10,000, a mutation rate of 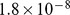 mutations/site/generation, and mutation-to-recombination rate ratios of 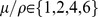 (i.e., recombination rates of 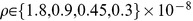 events/site/generation). One hundred replicate data sets were generated for each choice of 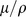. Alternative parameter values were used in certain cases, as noted in the text and figure captions.

#### Real data

Information about human polymorphisms came from the “69 Genomes” data set from Complete Genomics (CG) (http://www.completegenomics.com/public-data/69-Genomes). For each individual considered, we recorded the diploid genotype call reported for each position in the hg19 (Genome Reference Consortium Human Build 37) reference genome using CG's ‘masterVar’ files. We considered both “SNPs” and “length-preserving substitutions” in the masterVar file, and also noted positions where CG could not confidently assign a genotype. All other positions were assumed to be homozygous for the allele reported in the reference genome.

Borrowing from our previous work on demography inference [92], we applied several filters to these data to reduce the impact of technical errors from alignment, sequencing, genotype inference, and genome assembly. These filters include simple repeats, recent segmental duplications, and transposable elements. We phased the data using SHAPEIT v2 [59], guided by the pedigree information describing the relationships among the 69 individuals. After phasing, we removed the child in each trio, as well as all but the four grandparents in the 17-member CEU pedigree, leaving 54 unrelated individuals in our data set. From this set, we further filtered all CpG sites, sites with more than two observed alleles, and sites at which CG did not call a genotype in any of the 54 individuals. All genomic positions excluded by our filters were treated as missing data by *ARGweaver*, meaning that the program integrated over all possible nucleotides at these positions (as in [92]).

In order to account for region-specific variation in recombination and mutation rates, we used the HapMap phase II recombination map [106] and a mutation rate map estimated from alignments of several primate genomes, including chimpanzee (panTro2), orangutan (ponAbe2), and rhesus Macaque (rheMac2) [107]. Mutation rates were scaled to have an average of 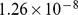 mutations/generation/site and were averaged over 100 kb non-overlapping windows. This value was obtained by assuming a genome-wide average of 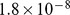 mutations/generation/site, and observing a 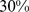 reduction in nucleotide diversity when the CpG filter was applied.

Calls of hypervariable and invariant regions were obtained from the CG FTP site (ftp://ftp2.completegenomics.com). Copy number variant calls for each individual were obtained from a file named cnvDetailsDiploidBeta, which was extracted from an ASM-VAR-files tar archive.

### Data Analysis

To sample ARGs genome-wide, we split each sequence alignment into non-overlapping segments of 2 Mb, flanked on each side by 100 kb of overlapping sequence. We chose a core set of 12 individuals (24 haplotypes) randomly such that each major population group was represented. We then used *ARGweaver* to sample ARGs for these genomes, assuming a population size of 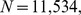 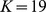 time steps, and a maximum time of 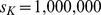 generations. Our prior estimate of 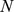 was based on an empirical estimate of 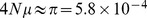 from the CG sequence data, and an assumption of 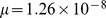 mutations per site per generation for non-CpG sites (see previous section). This initial step involved 500 sampling iterations, consisting of 100 initial iterations under an infinite sites assumption, and 400 iterations with the full finite sites model. The final sample from this initial step was used as a starting point for threading in the remaining genomes. Once these were threaded, we applied *ARGweaver* with infinite sites for 100 iterations, followed by 2400 iterations with the finite sites model. Samples were recorded every 10 iterations for the final 2000 iterations, for a total of 200 samples. For our genome-wide analyses, we integrated the separate 2.2 Mb analyses by setting a switchpoint at the middle of each overlapping 100 kb segment, in order to minimize boundary effects at the analyzed sites.

To compute the neutral CDFs in Figure S17, we used a set of putatively neutral regions obtained by removing all GENCODE (v15) genes plus 1000 bp flank on either side of each exon, as well as all mammalian phastCons elements plus 100 bp of flanking sequence. From the remaining portion of the genome, we sampled 1000 sets of 69 regions with the same distribution of lengths as the non-CpG regions identified by [77].

To estimate the allele age at each polymorphic site, we considered all local genealogies sampled at that position, discarding any sampled genealogies that required more than one mutation to explain the observed data. In addition, we required that all of the retained genealogies implied the same derived allele, excluding positions that violated this condition from our analysis. For the remaining cases, we estimated the allele age for each sample as the average age of the branch on which the mutation leading to the derived allele was assumed to occur by parsimony, and averaged this value across samples.

## Supporting Information

Figure S1ARGs simulated under Discretized Sequentially Markov Coalescent model are similar to those simulated under continueous models. ARGs were simulated using the coalescent-with-recombination (red), Sequentially Markov Coalescent (green), and Discretized Sequentially Markov Coalescent (blue). Three versions of the DSMC were considered: ones with with 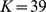 (dark blue), 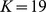 (medium blue), and 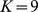 (light blue) time intervals. In all cases, we assumed 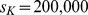 generations. Our standard simulation parameters were used (see Methods) except that sequences were of length 100 kb (rather than 1 Mb) to save in computation. (A) Numbers of recombinations at four different recombination rates corresponding to 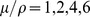 (in reverse order). To make the comparison fair, recombinations between nonancestral sequences (which are disallowed by the SMC/DSMC) are excluded in the case of the coalescent-with-recombination. However, “diamond” or “bubble” recombinations (ones that are immediately reversed by coalescence events, going backwards in time) were included, so any distortion from excluding these events in the SMC/DSMC is reflected in the figure. (B) Numbers of segregating sites at three different effective population sizes with 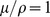.(PDF)

Figure S2 Illustration of “leaf trace.” An example leaf trace (highlighted in gray) is shown for a hypothetical 10-kb genomic segment and six haploid sequences. The ARG for these sequences contains two local trees (shown to left and right) separated by a single recombination event (red circle and arrow). In the leaf trace, each sequence is represented by a line, and these lines are ordered and spaced according to the local tree at each position. Spacing between adjacent lines is proportional to time to most recent common ancestry of associated sequences. (Notice, however, that it is not possible to impose a similar interpretation on non-adjacent lines in the diagram.) Nonrecombining genomic intervals are reflected by blocks of parallel lines. Recombinations lead to changes in spacing and/or order and produce vertical lines in the plot. Notice that aspects of the leaf ordering are arbitrary, because the two children between each ancestral node can be exchanged without altering the meaning of the diagram. In addition, this visualization device applies to a single ARG and does not easily generalize to distributions of possible ARGs. For our genome browser tracks, we use the single most likely ARG sampled by *ARGweaver* as the basis for the plots. Finally, note that the lines in the plot can be colored in various ways. In our current tracks, they are colored according to the population origin of each haploid sequence.
(PDF)

Figure S3Convergence of *ARGweaver* with simulated data. When the number of sequences exceeds 6–8, the Metropolis-Hastings algorithm and subtree threading operation are needed for *ARGweaver* to have acceptable convergence properties. This plot shows results for 20 1-Mb sequences, generated under our standard simulation parameters with 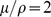 (Methods). Here the measure of convergence is the difference between the number of inferred recombination events and the number of true recombination events. Other measures show similar patterns.(PDF)

Figure S4Recovery of global features of simulated data for various values of 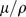. This figure is the same as Figure 4A, except that it shows results for four different values of the mutation-to-recombination rate ratio, ranging from 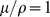 (bottom row) to 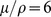 (top row). The second row from the bottom (with 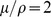) is identical to Figure 4A. Notice that high values of 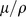 lead to reduced variance in all estimates, owing to larger numbers of mutations per local genealogy, but that the estimates remain reasonably accurate in all cases. However, there does appear to be a slight tendency to under-estimate the number of recombinations, particularly at low values of 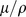, probably due to approximations inherent in the DSMC (see text). Note that these are generated by the full coalescent with recombination, not the DSMC.(PDF)

Figure S5Recovery of TMRCA along simulated sequences for various values of 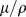. This figure is the same as Figure 4B except that it shows results for four diffrent values of the mutation-to-recombination rate ratio, ranging from 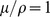 (bottom panel) to 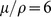 (top panel). Each panel represents one randomly selected simulated data set. Pearson's correlation coefficients (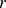) for true vs. estimated TMRCAs across all local trees are shown in the top right corner of each panel. As expected, the quality of the estimates generally improves with 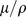, but this example suggests there is limited improvement above 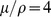.(PNG)

Figure S6Recovery of recombination rates from simulated data. We simulated an alignment of 100 sequences with 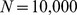 and 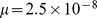, allowing for variable recombination rates based on estimates along the human genome. Despite the assumption in the prior of a constant recombination rate of 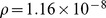, the posterior mean estimate of the average number of recombinations in a 1 kb sliding window (red line) correlates well with the true recombination rates used during simulation (black line). Notice that recombination hotspots are clearly identifiable by peaks in the inferred rates but the magnitudes of these peaks are dampened by the use of a uniform prior. Only recombinations that produced changes in tree topology (the class that is detectable by our methods) were considered for the plot of the true recombination rate.(PDF)

Figure S7Estimating ages of derived alleles in simulated data. (A,C,E,G) Inferred allele age correlates well with true allele age according to both Pearson's (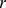) and Spearman's rank (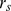) correlation coefficients. Correlation is strongest for high mutation/recombination rate ratios. Ages were estimated by calculating the midpoint of the branch on which the mutation was inferred to occur, under an infinite sites model, and averaging across sample from the posterior distribution. Points are colored on a spectrum from blue to green in proportion to derived allele frequencies. (B,D,F,H) Allele frequency has significantly lower correlation with true allele age, implying that the ARG will enable much better estimates of allele age than allele frequencies alone. Ages are measured in generations before the present. Our standard simulated data sets were used (Methods).(PDF)

Figure S8Recovery of local tree topologies. Sequences were simulated under the coalescent-with-recombination using our standard parameters (Methods), ARGs were inferred using *ARGweaver*, then 100 equally spaced local trees were extracted from the sampled ARGs. The topologies of these trees were compared with the true trees generated during simulation at corresponding positions in the alignment. We compared *ARGweaver* with the heuristic programs *Margarita* [34] and *treesim* using two measures: (A) branch correctness (one minus the normalized Robinson-Foulds (RF) distance [108]) and (B) Maximum Agreement Subtree (MAST) percentages (the size of the largest leaf-set such that induced subtrees are topologically equivalent, expressed as a percentage of the total number of leaves), across a range of mutation to recombination rate ratios (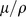). In both (A) and (B), error bars reflect one standard error assuming independence of 100 local trees ×10 simulation replicates.(PDF)

Figure S9Local tree branch posterior probabilities inferred by *ARGweaver* accurately reflect their probability of correctness. The branch posterior probabilities found by *ARGweaver* (red) and *treesim* (green) more accurately reflect the probability of the branch being correct than the frequency at which *Margarita* (blue) infers a branch. For each method, branches were binned by their posterior probability (windows of 5%) and compared against their frequency of branch correctness. Shaded regions represent the 95% binomial confidence interval. This plot is based on our standard simulated data set with 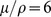. Posterior probabilities for *ARGweaver* are based on 1000 samples from the Markov chain, and the probabilities for *Margarita* and *treesim* reflect 100 independent samples.(PDF)

Figure S10Illustration of relative TMRCA halflife (RTH). Expected genealogies under (A) neutral drift, (B) background selection, and (C) a partial selective sweep. In each panel, the arrows to the left indicate the complete TMRCA (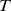) and the “half TMRCA” (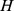), that is, the minimum time required for half of all lineages to find a single most recent common ancestor. The relative TMRCA halflife (RTH) is defined by the ratio 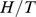. Because background selection (B) should primarily reduce the overall rate of coalescence, in a manner more or less homogeneous with respect to time, it is expected to have little effect on the RTH. Partial sweeps (C), however, will tend to produce a “burst” of coalescent events following a causal mutation (red circle), leading to reduced values of 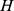. Nevertheless, because some lineages escape the sweep, the full TMRCA 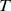 is likely to remain similar to its value under neutrality. As a result, the RTH will be reduced.(PDF)

Figure S11 Measures of genetic variation near protein-coding genes and partial selective sweeps for African populations. This figures is identical to Figure 5 except that it shows results for 17 African individuals or 34 haploid genomies (from the YRI, MKK, and LWK populations). Panel (A) is based on the same 17,845 protein-coding genes as in Figure 5A. Panel (B) is based on 271 100-kb regions predicted to have undergone partial selective sweeps in the YRI population based on the iHS statistic [72].
(PDF)

Figure S12 Time to most recent common ancestry (TMRCA) in the human leukocyte antigen (HLA) region. Genome browser track displaying the sitewise time to most recent common ancestry (TMRCA) estimated by *ARGweaver* based on the Complete Genomics individual human genome sequence data (track is available at http://genome-mirror.bscb.cornell.edu, assembly hg19). The human leukocyte antigen (HLA) region on human chromosome 6 contains many genomic intervals with extremely elevated expected TMRCAs, including four of the top 20 10-kb regions in the genome (highlighted here in gold; see descriptions in Table 2). The red line indicates the posterior mean of the TMRCA (estimated by averaging over the sampled local trees) and the blue lines above and below indicate a Bayesian 95% credible interval.
(PDF)

Figure S13 Mutation-rate normalized polymorphism rates in the 1000 Genomes Phase 1 data are elevated in the top twenty 10 kb regions by TMRCA. Shown are cumulative distribution fuctions for normalized polymorphism rates (computed as for Table 2) in all 10 kb windows across the human genome (black), the top twenty regions shown in Table 2 (red), and the fifteen regions not identified as possible CNVs in Table 2 (blue).
(PDF)

Figure S14 *ARGweaver* tracks near *KCNE4*. Shown is a ∼10-kb peak in the estimated TMRCA about 20 kb downstream of the *KCNE4* gene (shown in blue), which encodes a potassium voltage-gated channel strongly expressed in the embryo and adult uterus. The peak overlaps two ChIP-seq-supported transcription factor binding sites analyzed by Arbiza et al. [109] (“INSIGHT Regulatory Selection” track). The four tracks below the TMRCA plot show that the region in question displays elevated rates of both low-frequency (<10% derived allele frequency; shown in blue) and high-frequency (≥10%; shown in red) polymorphisms in the Complete Genomics data set, despite that divergence-based estimates of the mutation rate are at or below the genome-wide average (average values are indicated by horizontal black lines). *ARGweaver* explains these observations by inferring one of the deepest average TMRCAs in the human genome (#5 in Table 2). Additional tracks show no indication of copy number variation or recent duplications in this region. The leaf trace indicates that the signal for a deep TMRCA is driven by individuals from African populations (shown in green; the European and East Asian populations are shown in blue and red, respectively), suggesting that this region may contain ancient haplotypes specific to Africa.
(PDF)

Figure S15 *ARGweaver* tracks near *BCAR3*. Shown is a large region of elevated TMRCA in an intron of the *BCAR3* gene, which is involved in the development of anti-estrogen resistance in breast cancer. One 10-kb segment of this region has an average expected TMRCA of 377,017 generations, or approximately 9.4 My (#9 in Table 2). As in the previous example, this region shows elevated polymorphism rates but average or below-average mutation rates and overlaps ChIP-seq-supported transcription factor binding sites (INSIGHT track) [109]. Again, the regions of extreme TMRCA do not seem to be explained by copy number variation or recent duplications. In this case, however, the leaf trace demonstrates that the ancient haplotypes are distributed across all three major population groups (African = green, European = blue, East Asian = red).
(PDF)

Figure S16 *ARGweaver* tracks near *TULP4*. Another large region of elevated TMRCA upstream of the *TULP4* gene, which is thought to be involved in ubiquitination and proteosomal degradation and has a possible association with cleft lip. One 10-kb segment has an average expected TMRCA of 345,382 generations (8.6 My; #16 in Table 2). As in the previous two examples, this region has elevated polymorphism rates but not mutation rates, overlaps ChIP-seq-supported transcription factor binding sites (INSIGHT track), and does not seem to be an artifact of copy number variation or recent duplications.
(PDF)

Figure S17 Distribution of TMRCAs in regions predicted to be under balancing selection. Cumulative distribution functions (CDFs) are shown for the 125 regions identified by Leffler et al. [77] based on segregating haplotypes shared between humans and chimpanzees (black circles), the subset of 69 loci containing no shared polymorphisms in CpG dinucleotides (red circles) and a collection of 69 putatively neutral regions having the same length distribution. Neutral regions consisted of noncoding regions from which known genes, binding sites, and conserved elements had been removed (see [109]). Notice the pronounced shift toward larger TMRCAs in the regions predicted to be under balancing selection, and a slightly more pronounced shift for the subset not containing CpGs (which are less likely to have undergone parallel mutations on both lineages). TMRCAs are measured in generations, as in all other figures and tables.
(PDF)

Figure S18 *ARGweaver* tracks near locus containing segregating haplotypes shared in humans and chimpanzees. Elevated TMRCA corresponding to a region identified by Leffler et al. [77] between the *FREM3* and *GYPE* genes (#11 in Table 3; see black square in track at bottom). The shared polymorphisms in this region are in strong linkage disequilibrium with eQTLs for *GYPE*, a paralog of *GYPA*, which may be under balancing selection. The leaf trace indicates that the ancient haplotypes are shared across major human population groups (African = green, European = blue, East Asian = red).
(PDF)

Figure S19Reduction in mean allele age as a function of annotation class and derived allele frequency. This figure shows the same information as Figure 6B, but instead of plotting absolute values of the estimated allele ages, it plots the estimated *reduction* in allele age relative to neutrality, which is defined as the differences between the estimated age for each annotation type and the estimate for the corresponding neutral class (in generations). This representation shows clearly that the reduction in allele age increases with allele frequency much more rapidly for annotation classes under strong selection than for those under weak selection. The contrast between the nearly neutral classes (4d, PPh:Benign, CV:NonPath) and the strongly selected classes (PPh:ProbDam, CV:Path) is particularly striking. This difference can be understood as follows. Reductions in allele age at nearly neutral sites will primarily be a consequence of selection at linked sites, which, to a first approximation, will decrease the local effective population size. This will have the effect of approximately re-scaling allele ages by a constant factor across all ages, making the reduction in age roughly proportional to the absolute age. Mutations under stronger direct selection, by contrast, will spend disproportionally less time at higher frequencies, making their reductions in age at high frequencies disproportionally larger than those for nearly neutral mutations (see [79]). This effect will occur even in the absence of dominance (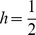), but it could be exascerbated by dominance, which will tend to make low-frequency alleles invisible to direct selection. In any case, this plot shows that selection from linked sites can produce comparable, or even larger, reductions in age than direct selection at low allele frequencies, but at high frequencies, direct selection tends to dominate in age reduction.(PDF)

Figure S20 Human population phylogenies inferred from sampled ancestral recombination graphs. Phylogenetic networks for the eleven populations represented in the Complete Genomics data set were reconstructed using the PhyloNet program [90], [91]. As input to PhyloNet, we used 2,304 local trees extracted from the ARG at approximately 1 Mb intervals, with one randomly sampled chromosome per population (see Text S1). (A) Population phylogeny inferred in the absence of hybridization/admixture, showing the expected primary relationships among populations. (B) Population networks inferred when between one and five hybridization nodes are allowed. Populations inferred to be admixed are indicated by gray lines and the inferred hybridization nodes are shown as gray circles. Numbers indicate the order in which these nodes appear. For example, when one hybridization node is allowed, the MKK population is inferred to be admixed, and when two are allowed, the MXL population is also inferred to be admixed. The inferred network is consistent with other recent studies in many respects, but PhyloNet is unable to reconstruct the precise topology of the complex subnetwork consisting of the GIH, MXL, PUR, CEU, and TSI populations (see Text S1). Population names follow the convention used by the HapMap 3 and 1000 Genomes projects: CHB = Han Chinese in Beijing, China; JPT = Japanese in Tokyo, Japan; GIH = Gujarati Indians in Houston, Texas; MXL = Mexican ancestry in Los Angeles, California; PUR = Puerto Ricans in Puerto Rico; CEU = Utah residents with Northern and Western European ancestry from the Centre d'Etude de Polymorphisme Humain (CEPH) collection; TSI = Toscani in Italy; MKK = Maasai in Kinyawa, Kenya; LWK = Luhya in Webuye, Kenya; ASW = African ancestry in Southwest USA; YRI = Yoruba in Ibadan, Nigeria.
(PDF)

Figure S21Cases for new recombination 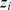 given re-coalescence point 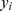. (A) In the main case, the recombination 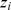 (blue point) occurs on the branch that is being threaded into the ARG (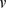; shown in red). After a recombination on this branch, a re-coalescence can occur at any point 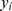 (green points) in the local tree 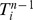 such that 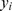 is at least as old as 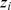. Therefore, when enumerating the possible 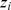 consistent with a given 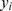, one must consider all points on branch 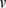 at least as recent as 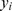. This set is denoted 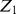 in the text. (B) There is an additional special case to consider when branch 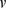 coalesces to the same branches of 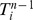 at positions 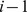 and 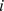, that is, when 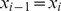. In this case, it is possible that the recombination 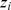 (blue point) occurs not on the new branch 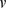 but on 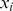 (black branch) at a time point no older than the re-coalescence time 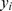 (green points). A recombination of this kind will leave an identical signature to the symmetric case of a recombination on 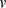 in the same time interval followed by a re-coalescence of 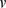 to 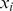. Therefore, when enumerating the possible 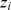 consistent with a given 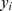 such that 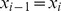, one must also consider the set 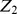 consisting of all 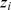 on 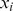 such that 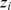 is at least as recent as 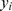. Notice that, in both (A) and (B), the tree excluding 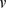 is unchanged by all recombination and coalescence scenarios 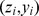 under consideration, i.e., 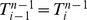 (black branches).(PDF)

Text S1 Supplementary methods and analyses.
(PDF)

## References

[1] Hein J, Schierup M, Wiuf C (2005) Gene genealogies, variation and evolution: a primer in coalescent theory. Oxford: Oxford University Press.

[2] Wakeley J (2009) Coalescent theory: an introduction. Greenwood Village: Roberts & Co. Publishers.

[3] Fisher RA (1930) The Genetical Theory of Natural Selection. Oxford: Oxford University Press.

[4] Wright S (1931) Evolution in Mendelian Populations. Genetics 16: 97–159. 17246615 10.1093/genetics/16.2.97 PMC1201091

[5] Kimura M (1962) On the probability of fixation of mutant genes in a population. Genetics 47: 713–719. 14456043 10.1093/genetics/47.6.713 PMC1210364

[6] Felsenstein J (1973) Maximum-likelihood and minimum-step methods for estimating evolutionary trees from data on discrete characters. Syst Zool 22: 240–249.

[7] Felsenstein J (1981) Evolutionary trees from DNA sequences: a maximum likelihood approach. J Mol Evol 17: 368–376. 7288891 10.1007/BF01734359

[8] Menozzi P, Piazza A, Cavalli-Sforza L (1978) Synthetic maps of human gene frequencies in Europeans. Science 201: 786–792. 356262 10.1126/science.356262

[9] Kingman J (1982) The coalescent. Stoch Process Appl 13: 235–248.

[10] Sawyer SA, Hartl DL (1992) Population genetics of polymorphism and divergence. Genetics 132: 1161–1176. 1459433 10.1093/genetics/132.4.1161 PMC1205236

[11] Voight BF, Adams AM, Frisse LA, Qian Y, Hudson RR, et al (2005) Interrogating multiple aspects of variation in a full resequencing data set to infer human population size changes. Proc Natl Acad Sci USA 102: 18508–18513. 16352722 10.1073/pnas.0507325102 PMC1311907

[12] Keightley PD, Eyre-Walker A (2007) Joint inference of the distribution of fitness effects of deleterious mutations and population demography based on nucleotide polymorphism frequencies. Genetics 177: 2251–2261. 18073430 10.1534/genetics.107.080663 PMC2219502

[13] Boyko AR, Williamson SH, Indap AR, Degenhardt JD, Hernandez RD, et al (2008) Assessing the evolutionary impact of amino acid mutations in the human genome. PLoS Genet 4: e1000083. 18516229 10.1371/journal.pgen.1000083 PMC2377339

[14] Lawson DJ, Hellenthal G, Myers S, Falush D (2012) Inference of population structure using dense haplotype data. PLoS Genet 8: e1002453. 22291602 10.1371/journal.pgen.1002453 PMC3266881

[15] Palamara PF, Lencz T, Darvasi A, Pe'er I (2012) Length distributions of identity by descent reveal fine-scale demographic history. Am J Hum Genet 91: 809–822. 23103233 10.1016/j.ajhg.2012.08.030 PMC3487132

[16] Ralph P, Coop G (2013) The geography of recent genetic ancestry across Europe. PLoS Biol 11: e1001555. 23667324 10.1371/journal.pbio.1001555 PMC3646727

[17] Harris K, Nielsen R (2013) Inferring demographic history from a spectrum of shared haplotype lengths. PLoS Genet 9: e1003521. 23754952 10.1371/journal.pgen.1003521 PMC3675002

[18] Hudson RR (1991) Gene genealogies and the coalescent process. In: Futuyma D, Antonovics J, editors. Oxford Surveys in Evolutionary Biology, volume 7. pp. 1–44.

[19] Griffiths RC, Marjoram P (1996) Ancestral inference from samples of DNA sequences with recombination. J Comput Biol 3: 479–502. 9018600 10.1089/cmb.1996.3.479

[20] Griffiths R, Marjoram P (1997) An ancestral recombination graph. In: Donnelly P, Tavaré S, editors, Progress in Population Genetics and Human Evolution. Springer Verlag. pp. 257–270.

[21] Hudson RR (1983) Properties of a neutral allele model with intragenic recombination. Theor Popul Biol 23: 183–201. 6612631 10.1016/0040-5809(83)90013-8

[22] Fearnhead P, Donnelly P (2001) Estimating recombination rates from population genetic data. Genetics 159: 1299–1318. 11729171 10.1093/genetics/159.3.1299 PMC1461855

[23] Stephens M, Donnelly P (2000) Inference in molecular population genetics. Journal of the Royal Statistical Society Series B (Statistical Methodology) 62: 605–655.

[24] Kuhner MK, Yamato J, Felsenstein J (2000) Maximum likelihood estimation of recombination rates from population data. Genetics 156: 1393–1401. 11063710 10.1093/genetics/156.3.1393 PMC1461317

[25] Nielsen R (2000) Estimation of population parameters and recombination rates from single nucleotide polymorphisms. Genetics 154: 931–942. 10655242 10.1093/genetics/154.2.931 PMC1460954

[26] Kuhner MK (2006) LAMARC 2.0: maximum likelihood and Bayesian estimation of population parameters. Bioinformatics 22: 768–770. 16410317 10.1093/bioinformatics/btk051

[27] O'Fallon BD (2013) ACG: rapid inference of population history from recombining nucleotide sequences. BMC Bioinformatics 14: 40. 23379678 10.1186/1471-2105-14-40 PMC3575405

[28] Hein J (1990) Reconstructing evolution of sequences subject to recombination using parsimony. Math Biosci 98: 185–200. 2134501 10.1016/0025-5564(90)90123-g

[29] Hein J (1993) A heuristic method to reconstruct the history of sequences subject to recombination. J Mol Evol 36: 396–405.

[30] Kececioglu J, Gusfield D (1998) Reconstructing a history of recombinations from a set of sequences. Discrete Applied Mathematics 88: 239–260.

[31] Wang L, Zhang K, Zhang L (2001) Perfect phylogenetic networks with recombination. J Comput Biol 8: 69–78. 11339907 10.1089/106652701300099119

[32] Song YS, Hein J (2005) Constructing minimal ancestral recombination graphs. J Comput Biol 12: 147–169. 15767774 10.1089/cmb.2005.12.147

[33] Song YS, Wu Y, Gusfield D (2005) Efficient computation of close lower and upper bounds on the minimum number of recombinations in biological sequence evolution. Bioinformatics 21 Suppl 1: i413–422. 15961486 10.1093/bioinformatics/bti1033

[34] Minichiello MJ, Durbin R (2006) Mapping trait loci by use of inferred ancestral recombination graphs. Am J Hum Genet 79: 910–922. 17033967 10.1086/508901 PMC1698562

[35] Wu Y (2009) New methods for inference of local tree topologies with recombinant SNP sequences in populations. IEEE/ACM Trans Comput Biol Bioinform 8: 182–193. 21071806 10.1109/TCBB.2009.27

[36] Wiuf C, Hein J (1999) Recombination as a point process along sequences. Theor Popul Biol 55: 248–259. 10366550 10.1006/tpbi.1998.1403

[37] McVean GAT, Cardin NJ (2005) Approximating the coalescent with recombination. Philos Trans R Soc Lond B Biol Sci 360: 1387–1393. 16048782 10.1098/rstb.2005.1673 PMC1569517

[38] Marjoram P, Wall JD (2006) Fast “coalescent” simulation. BMC Genet 7: 16. 16539698 10.1186/1471-2156-7-16 PMC1458357

[39] Hobolth A, Christensen OF, Mailund T, Schierup MH (2007) Genomic relationships and speciation times of human, chimpanzee, and gorilla inferred from a coalescent hidden Markov model. PLoS Genet 3: e7. 17319744 10.1371/journal.pgen.0030007 PMC1802818

[40] Mailund T, Dutheil JY, Hobolth A, Lunter G, Schierup MH (2011) Estimating divergence time and ancestral effective population size of Bornean and Sumatran orangutan subspecies using a coalescent hidden Markov model. PLoS Genet 7: e1001319. 21408205 10.1371/journal.pgen.1001319 PMC3048369

[41] Mailund T, Halager AE, Westergaard M, Dutheil JY, Munch K, et al (2012) A new isolation with migration model along complete genomes infers very different divergence processes among closely related great ape species. PLoS Genet 8: e1003125. 23284294 10.1371/journal.pgen.1003125 PMC3527290

[42] Li H, Durbin R (2011) Inference of human population history from individual whole-genome sequences. Nature 475: 493–496. 21753753 10.1038/nature10231 PMC3154645

[43] Li N, Stephens M (2003) Modeling linkage disequilibrium and identifying recombination hotspots using single-nucleotide polymorphism data. Genetics 165: 2213–2233. 14704198 10.1093/genetics/165.4.2213 PMC1462870

[44] Stephens M, Scheet P (2005) Accounting for decay of linkage disequilibrium in haplotype inference and missing-data imputation. Am J Hum Genet 76: 449–462. 15700229 10.1086/428594 PMC1196397

[45] Marchini J, Howie B, Myers S, McVean G, Donnelly P (2007) A new multipoint method for genome-wide association studies by imputation of genotypes. Nat Genet 39: 906–913. 17572673 10.1038/ng2088

[46] Howie BN, Donnelly P, Marchini J (2009) A flexible and accurate genotype imputation method for the next generation of genome-wide association studies. PLoS Genet 5: e1000529. 19543373 10.1371/journal.pgen.1000529 PMC2689936

[47] Price AL, Tandon A, Patterson N, Barnes KC, Rafaels N, et al (2009) Sensitive detection of chromosomal segments of distinct ancestry in admixed populations. PLoS Genet 5: e1000519. 19543370 10.1371/journal.pgen.1000519 PMC2689842

[48] Li Y, Willer CJ, Ding J, Scheet P, Abecasis GR (2010) MaCH: using sequence and genotype data to estimate haplotypes and unobserved genotypes. Genet Epidemiol 34: 816–834. 21058334 10.1002/gepi.20533 PMC3175618

[49] Paul JS, Song YS (2010) A principled approach to deriving approximate conditional sampling distributions in population genetics models with recombination. Genetics 186: 321–338. 20592264 10.1534/genetics.110.117986 PMC2940296

[50] Paul JS, Steinrücken M, Song YS (2011) An accurate sequentially Markov conditional sampling distribution for the coalescent with recombination. Genetics 187: 1115–1128. 21270390 10.1534/genetics.110.125534 PMC3070520

[51] Sheehan S, Harris K, Song YS (2013) Estimating variable effective population sizes from multiple genomes: a sequentially Markov conditional sampling distribution approach. Genetics 194: 647–662. 23608192 10.1534/genetics.112.149096 PMC3697970

[52] Steinrucken M, Paul JS, Song YS (2013) A sequentially Markov conditional sampling distribution for structured populations with migration and recombination. Theor Popul Biol 87: 51–61. 23010245 10.1016/j.tpb.2012.08.004 PMC3532580

[53] Jukes TH, Cantor CR (1969) Evolution of protein molecules. In: Munro H, editor, Mammalian Protein Metabolism, New York: Academic Press. pp. 21–132.

[54] Husmeier D, Wright F (2001) Detection of recombination in DNA multiple alignments with hidden Markov models. J Comput Biol 8: 401–427. 11571075 10.1089/106652701752236214

[55] Kong A, Gudbjartsson DF, Sainz J, Jonsdottir GM, Gudjonsson SA, et al (2002) A high-resolution recombination map of the human genome. Nat Genet 31: 241–247. 12053178 10.1038/ng917

[56] Kong A, Frigge ML, Masson G, Besenbacher S, Sulem P, et al (2012) Rate of de novo mutations and the importance of father's age to disease risk. Nature 488: 471–475. 22914163 10.1038/nature11396 PMC3548427

[57] Sun JX, Helgason A, Masson G, Ebenesersdottir SS, Li H, et al (2012) A direct characterization of human mutation based on microsatellites. Nat Genet 44: 1161–1165. 22922873 10.1038/ng.2398 PMC3459271

[58] Drmanac R, Sparks AB, Callow MJ, Halpern AL, Burns NL, et al (2010) Human genome sequencing using unchained base reads on self-assembling dna nanoarrays. Science 327: 78–81. 19892942 10.1126/science.1181498

[59] Delaneau O, Zagury JF, Marchini J (2013) Improved whole-chromosome phasing for disease and population genetic studies. Nat Methods 10: 5–6. 23269371 10.1038/nmeth.2307

[60] McVicker G, Gordon D, Davis C, Green P (2009) Widespread genomic signatures of natural selection in hominid evolution. PLoS Genet 5: e1000471. 19424416 10.1371/journal.pgen.1000471 PMC2669884

[61] Cai JJ, Macpherson JM, Sella G, Petrov DA (2009) Pervasive hitchhiking at coding and regulatory sites in humans. PLoS Genet 5: e1000336. 19148272 10.1371/journal.pgen.1000336 PMC2613029

[62] Hernandez RD, Kelley JL, Elyashiv E, Melton SC, Auton A, et al (2011) Classic selective sweeps were rare in recent human evolution. Science 331: 920–924. 21330547 10.1126/science.1198878 PMC3669691

[63] Gottipati S, Arbiza L, Siepel A, Clark AG, Keinan A (2011) Analyses of X-linked and autosomal genetic variation in population-scale whole genome sequencing. Nat Genet 43: 741–743. 21775991 10.1038/ng.877 PMC3145052

[64] Lohmueller KE, Albrechtsen A, Li Y, Kim SY, Korneliussen T, et al (2011) Natural selection affects multiple aspects of genetic variation at putatively neutral sites across the human genome. PLoS Genet 7: e1002326. 22022285 10.1371/journal.pgen.1002326 PMC3192825

[65] Charlesworth B, Morgan MT, Charlesworth D (1993) The effect of deleterious mutations on neutral molecular variation. Genetics 134: 1289–1303. 8375663 10.1093/genetics/134.4.1289 PMC1205596

[66] Hudson RR, Kaplan NL (1995) Deleterious background selection with recombination. Genetics 141: 1605–1617. 8601498 10.1093/genetics/141.4.1605 PMC1206891

[67] Nordborg M, Charlesworth B, Charlesworth D (1996) The effect of recombination on background selection. Genet Res 67: 159–174. 8801188 10.1017/s0016672300033619

[68] Charlesworth B (2012) The effects of deleterious mutations on evolution at linked sites. Genetics 190: 5–22. 22219506 10.1534/genetics.111.134288 PMC3249359

[69] Maynard Smith J, Haigh J (1974) The hitch-hiking effect of a favourable gene. Genet Res 23: 23–35. 4407212

[70] Barton NH (1998) The effect of hitch-hiking on neutral genealogies. Genet Res 72: 123–133.

[71] Walczak AM, Nicolaisen LE, Plotkin JB, Desai MM (2012) The structure of genealogies in the presence of purifying selection: a fitness-class coalescent. Genetics 190: 753–779. 22135349 10.1534/genetics.111.134544 PMC3276618

[72] Voight BF, Kudaravalli S, Wen X, Pritchard JK (2006) A map of recent positive selection in the human genome. PLoS Biol 4: e72. 16494531 10.1371/journal.pbio.0040072 PMC1382018

[73] Hughes AL, Nei M (1988) Pattern of nucleotide substitution at major histocompatibility complex class I loci reveals overdominant selection. Nature 335: 167–170. 3412472 10.1038/335167a0

[74] Apanius V, Penn D, Slev PR, Ruff LR, Potts WK (1997) The nature of selection on the major histocompatibility complex. Crit Rev Immunol 17: 179–224. 9094452 10.1615/critrevimmunol.v17.i2.40

[75] Hughes AL, Yeager M (1998) Natural selection at major histocompatibility complex loci of vertebrates. Annu Rev Genet 32: 415–435. 9928486 10.1146/annurev.genet.32.1.415

[76] Hodgkinson A, Eyre-Walker A (2010) The genomic distribution and local context of coincident SNPs in human and chimpanzee. Genome Biol Evol 2: 547–557. 20675616 10.1093/gbe/evq039 PMC2997558

[77] Leffler EM, Ziyue G, Pfeifer S, Segurel L, Auton A, et al (2013) Multiple instances of ancient balancing selection shared between humans and chimpanzees. Science 339: 1578–1582. 23413192 10.1126/science.1234070 PMC3612375

[78] Maruyama T (1974) The age of a rare mutant gene in a large population. Am J Hum Genet 26: 669–673. 4440678 PMC1762845

[79] Kiezun A, Pulit SL, Francioli LC, van Dijk F, Swertz M, et al (2013) Deleterious alleles in the human genome are on average younger than neutral alleles of the same frequency. PLoS Genet 9: e1003301. 23468643 10.1371/journal.pgen.1003301 PMC3585140

[80] Hill WG, Robertson A (1966) The effect of linkage on limits to artificial selection. Genet Res 8: 269–294. 5980116

[81] Karlin S, McGregor J (1968) Rates and probabilities of fixation for two locus random mating finite populations without selection. Genetics 58: 141–159. 5656343 10.1093/genetics/58.1.141 PMC1211843

[82] Strobeck C, Morgan K (1978) The effect of intragenic recombination on the number of alleles in a finite population. Genetics 88: 829–844. 17248821 10.1093/genetics/88.4.829 PMC1213820

[83] Griffiths RC (1981) Neutral two-locus multiple allele models with recombination. Theor Popul Biol 19: 169–186.

[84] Rannala B, Reeve JP (2001) High-resolution multipoint linkage-disequilibrium mapping in the context of a human genome sequence. Am J Hum Genet 69: 159–178. 11410841 10.1086/321279 PMC1226031

[85] Larribe F, Lessard S, Schork NJ (2002) Gene mapping via the ancestral recombination graph. Theor Popul Biol 62: 215–229. 12167358 10.1006/tpbi.2002.1601

[86] Zollner S, Pritchard JK (2005) Coalescent-based association mapping and fine mapping of complex trait loci. Genetics 169: 1071–1092. 15489534 10.1534/genetics.104.031799 PMC1449137

[87] Wu Y (2008) Association mapping of complex diseases with ancestral recombination graphs: models and efficient algorithms. J Comput Biol 15: 667–684. 18651799 10.1089/cmb.2007.0116

[88] Besenbacher S, Mailund T, Schierup MH (2009) Local phylogeny mapping of quantitative traits: higher accuracy and better ranking than single-marker association in genomewide scans. Genetics 181: 747–753. 19064712 10.1534/genetics.108.092643 PMC2644962

[89] Prado-Martinez J, Sudmant PH, Kidd JM, Li H, Kelley JL, et al (2013) Great ape genetic diversity and population history. Nature 499: 471–475. 23823723 10.1038/nature12228 PMC3822165

[90] Than C, Nakhleh L (2009) Species tree inference by minimizing deep coalescences. PLoS Comput Biol 5: e1000501. 19749978 10.1371/journal.pcbi.1000501 PMC2729383

[91] Yu Y, Barnett RM, Nakhleh L (2013) Parsimonious inference of hybridization in the presence of incomplete lineage sorting. Syst Biol 62: 738–751. 23736104 10.1093/sysbio/syt037 PMC3739885

[92] Gronau I, Hubisz MJ, Gulko B, Danko CG, Siepel A (2011) Bayesian inference of ancient human demography from individual genome sequences. Nature Genetics 43: 1031–1034. 21926973 10.1038/ng.937 PMC3245873

[93] Tang H, Coram M, Wang P, Zhu X, Risch N (2006) Reconstructing genetic ancestry blocks in admixed individuals. Am J Hum Genet 79: 1–12. 16773560 10.1086/504302 PMC1474129

[94] Sankararaman S, Sridhar S, Kimmel G, Halperin E (2008) Estimating local ancestry in admixed populations. Am J Hum Genet 82: 290–303. 18252211 10.1016/j.ajhg.2007.09.022 PMC2664993

[95] Scheet P, Stephens M (2006) A fast and flexible statistical model for large-scale population genotype data: applications to inferring missing genotypes and haplotypic phase. Am J Hum Genet 78: 629–644. 16532393 10.1086/502802 PMC1424677

[96] Browning SR, Browning BL (2007) Rapid and accurate haplotype phasing and missing-data inference for whole-genome association studies by use of localized haplotype clustering. Am J Hum Genet 81: 1084–1097. 17924348 10.1086/521987 PMC2265661

[97] McVean GA, Myers SR, Hunt S, Deloukas P, Bentley DR, et al (2004) The fine-scale structure of recombination rate variation in the human genome. Science 304: 581–584. 15105499 10.1126/science.1092500

[98] Hobolth A, Jensen JL (2014) Markovian approximation to the finite loci coalescent with recombination along multiple sequences. Theor Popul Biol 2014: S0040–5809 doi:10.1016/j.tpb.2014.01.002 24486389 10.1016/j.tpb.2014.01.002

[99] Wu Y, Gusfield D (2007) Efficient computation of minimum recombination with genotypes (not haplotypes). Journal of Bioinformatics and Computational Biology 181–200. 17589959 10.1142/s0219720007002631

[100] Tavare S (1984) Line-of-descent and genealogical processes, and their applications in population genetics models. Theor Popul Biol 26: 119–164. 6505980 10.1016/0040-5809(84)90027-3

[101] Siepel A, Haussler D (2005) Phylogenetic hidden Markov models. In: Nielsen R, editor, Statistical Methods in Molecular Evolution, New York: Springer. pp. 325–351.

[102] Rabiner LR (1989) A tutorial on hidden Markov models and selected applications in speech recognition. Proceedings of the IEEE 77: 257–286.

[103] Durbin R, Eddy S, Krogh A, Mitchison G (1998) Biological Sequence Analysis: Probabilistic Models of Proteins and Nucleic Acids. Cambridge, UK: Cambridge University Press.

[104] Cawley SL, Pachter L (2003) HMM sampling and applications to gene finding and alternative splicing. Bioinformatics 19 Suppl 2: II36–II41. 14534169 10.1093/bioinformatics/btg1057

[105] Zhu J, Liu JS, Lawrence CE (1998) Bayesian adaptive sequence alignment algorithms. Bioinformatics 14: 25–39. 9520499 10.1093/bioinformatics/14.1.25

[106] International HapMap Consortium (2007) Frazer KA, Ballinger DG, Cox DR, Hinds DA, et al (2007) A second generation human haplotype map of over 3.1 million snps. Nature 449: 851–861. 17943122 10.1038/nature06258 PMC2689609

[107] Gronau I, Arbiza L, Mohammed J, Siepel A (2013) Inference of natural selection from interspersed genomic elements based on polymorphism and divergence. Mol Biol Evol 30: 1159–1171. 23386628 10.1093/molbev/mst019 PMC3697874

[108] Robinson DF, Foulds LR (1981) Comparison of phylogenetic trees. Mathematical Biosciences 53: 131–147.

[109] Arbiza L, Gronau I, Aksoy BA, Hubisz MJ, Gulko B, et al (2013) Genome-wide inference of natural selection on human transcription factor binding sites. Nat Genet 45: 723–729. 23749186 10.1038/ng.2658 PMC3932982

