# Supplementary figures and images for: Genome-Wide Inference of Ancestral Recombination Graphs
Source: PLoS Genet. 2014 May 15;10(5):e1004342. doi: 10.1371/journal.pgen.1004342 (PMC4022496; doi:10.1371/journal.pgen.1004342)

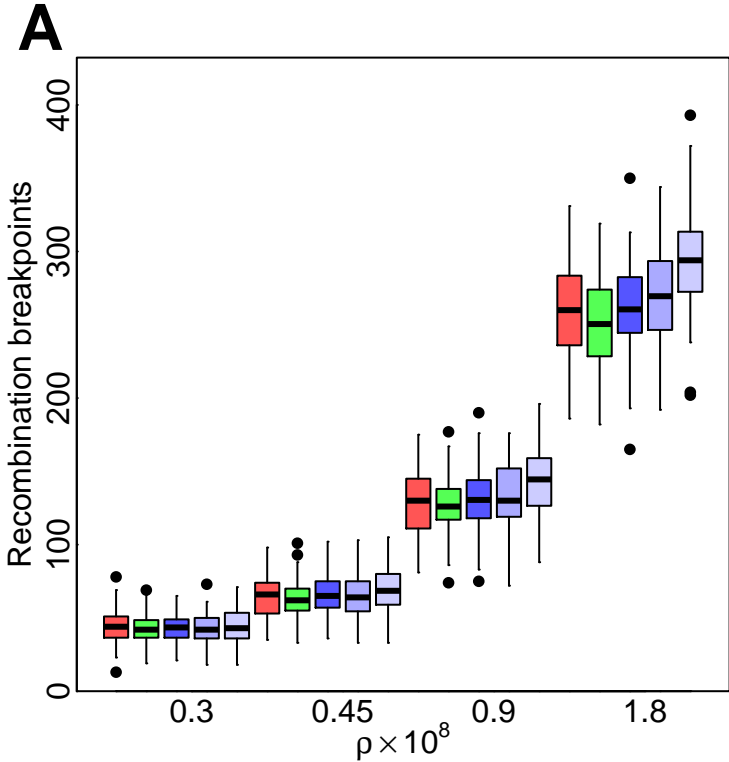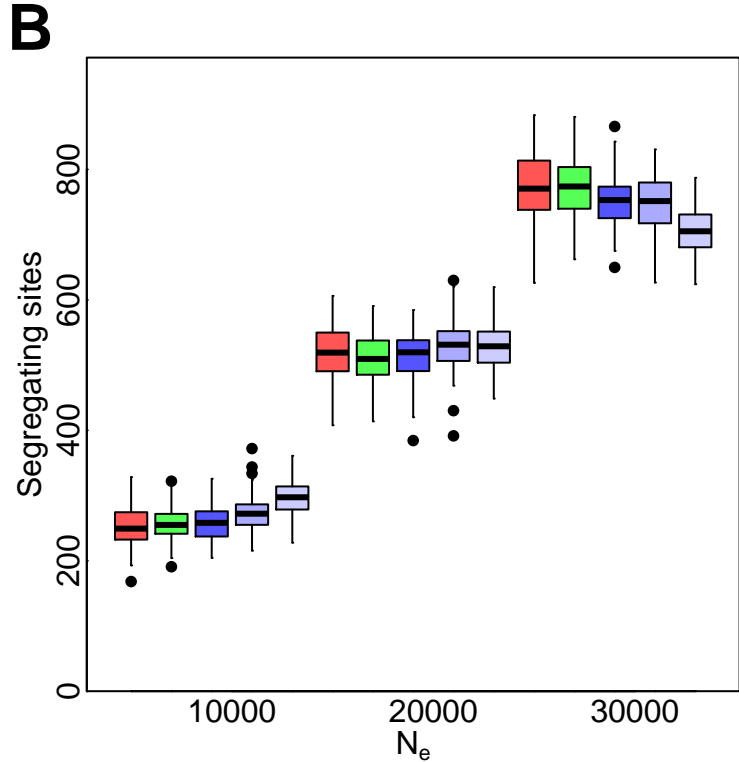

Supplement: Figure S1 — ARGs simulated under Discretized Sequentially Markov Coalescent model are similar to those simulated under continueous models. ARGs were simulated using the coalescent-with-recombination (red), Sequentially Markov Coalescent (green), and Discretized Sequentially Markov Coalescent (blue). Three versions of the DSMC were considered: ones with with (dark blue), (medium blue), and (light blue) time intervals. In all cases, we assumed generations. Our standard simulation parameters were used (see Methods) except that sequences were of length 100 kb (rather than 1 Mb) to save in computation. (A) Numbers of recombinations at four different recombination rates corresponding to (in reverse order). To make the comparison fair, recombinations between nonancestral sequences (which are disallowed by the SMC/DSMC) are excluded in the case of the coalescent-with-recombination. However, “diamond” or “bubble” recombinations (ones that are immediately reversed by coalescence events, going backwards in time) were included, so any distortion from excluding these events in the SMC/DSMC is reflected in the figure. (B) Numbers of segregating sites at three different effective population sizes with . (PDF) [file pgen.1004342.s001.pdf]

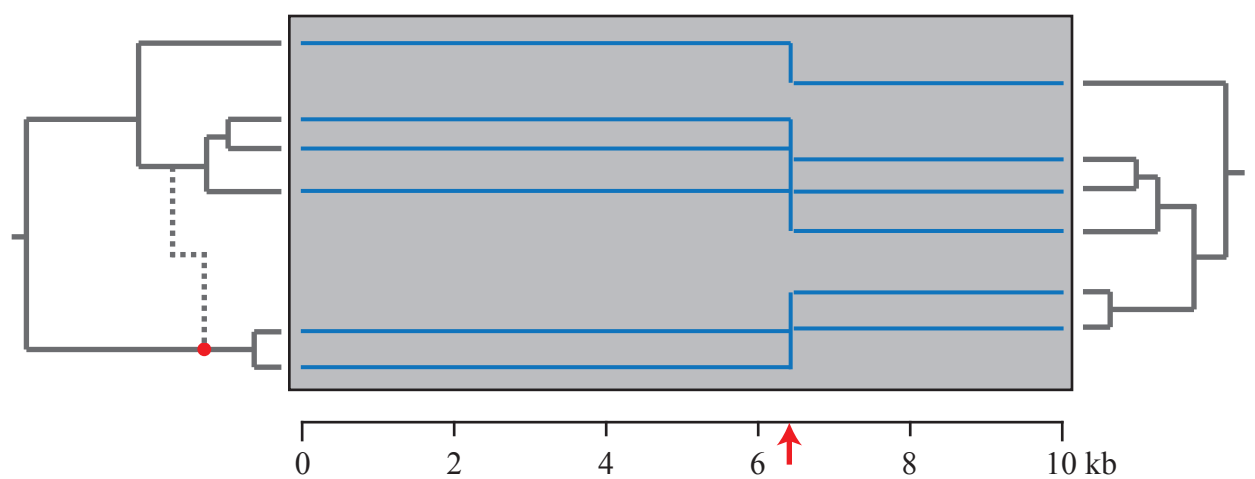

Supplement: Figure S2 — Illustration of “leaf trace.” An example leaf trace (highlighted in gray) is shown for a hypothetical 10-kb genomic segment and six haploid sequences. The ARG for these sequences contains two local trees (shown to left and right) separated by a single recombination event (red circle and arrow). In the leaf trace, each sequence is represented by a line, and these lines are ordered and spaced according to the local tree at each position. Spacing between adjacent lines is proportional to time to most recent common ancestry of associated sequences. (Notice, however, that it is not possible to impose a similar interpretation on non-adjacent lines in the diagram.) Nonrecombining genomic intervals are reflected by blocks of parallel lines. Recombinations lead to changes in spacing and/or order and produce vertical lines in the plot. Notice that aspects of the leaf ordering are arbitrary, because the two children between each ancestral node can be exchanged without altering the meaning of the diagram. In addition, this visualization device applies to a single ARG and does not easily generalize to distributions of possible ARGs. For our genome browser tracks, we use the single most likely ARG sampled by ARGweaver as the basis for the plots. Finally, note that the lines in the plot can be colored in various ways. In our current tracks, they are colored according to the population origin of each haploid sequence. (PDF) [file pgen.1004342.s002.pdf]

inferred minus true recombinations

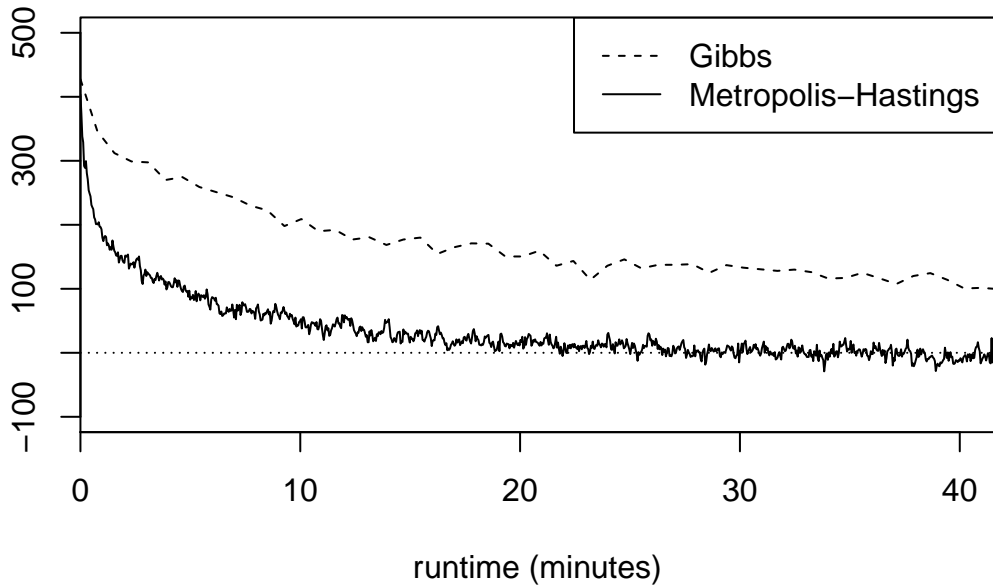

Supplement: Figure S3 — Convergence of ARGweaver with simulated data. When the number of sequences exceeds 6–8, the Metropolis-Hastings algorithm and subtree threading operation are needed for ARGweaver to have acceptable convergence properties. This plot shows results for 20 1-Mb sequences, generated under our standard simulation parameters with (Methods). Here the measure of convergence is the difference between the number of inferred recombination events and the number of true recombination events. Other measures show similar patterns. (PDF) [file pgen.1004342.s003.pdf]

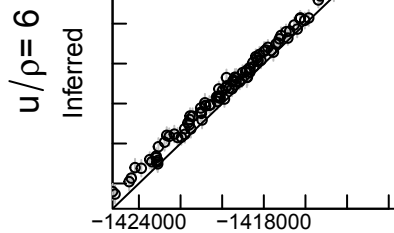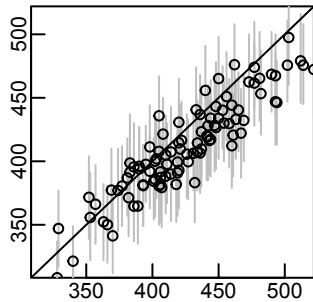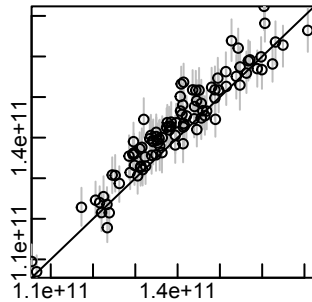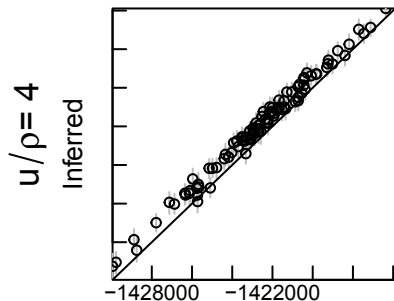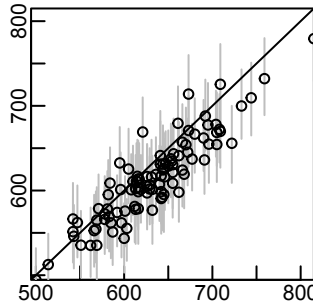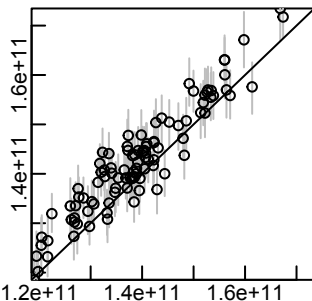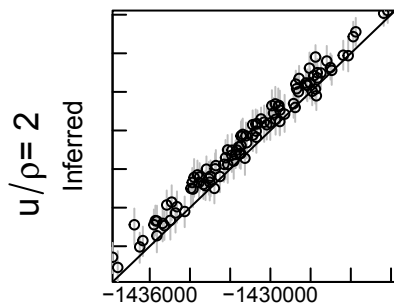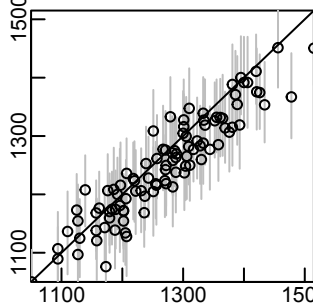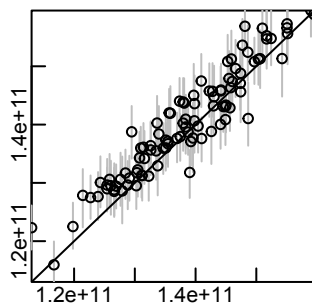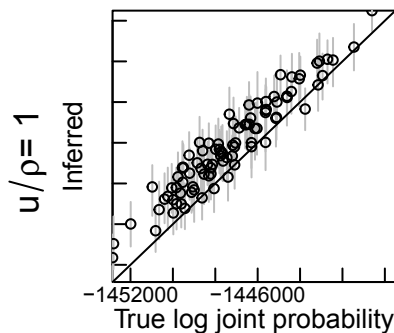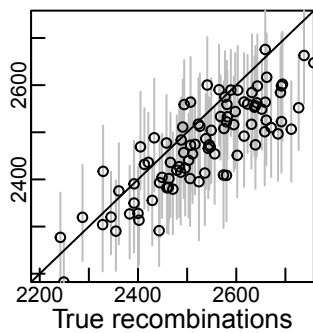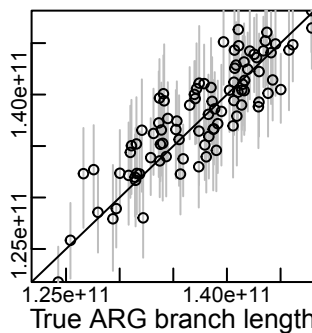

Supplement: Figure S4 — Recovery of global features of simulated data for various values of . This figure is the same as Figure 4A, except that it shows results for four different values of the mutation-to-recombination rate ratio, ranging from (bottom row) to (top row). The second row from the bottom (with ) is identical to Figure 4A. Notice that high values of lead to reduced variance in all estimates, owing to larger numbers of mutations per local genealogy, but that the estimates remain reasonably accurate in all cases. However, there does appear to be a slight tendency to under-estimate the number of recombinations, particularly at low values of , probably due to approximations inherent in the DSMC (see text). Note that these are generated by the full coalescent with recombination, not the DSMC. (PDF) [file pgen.1004342.s004.pdf]

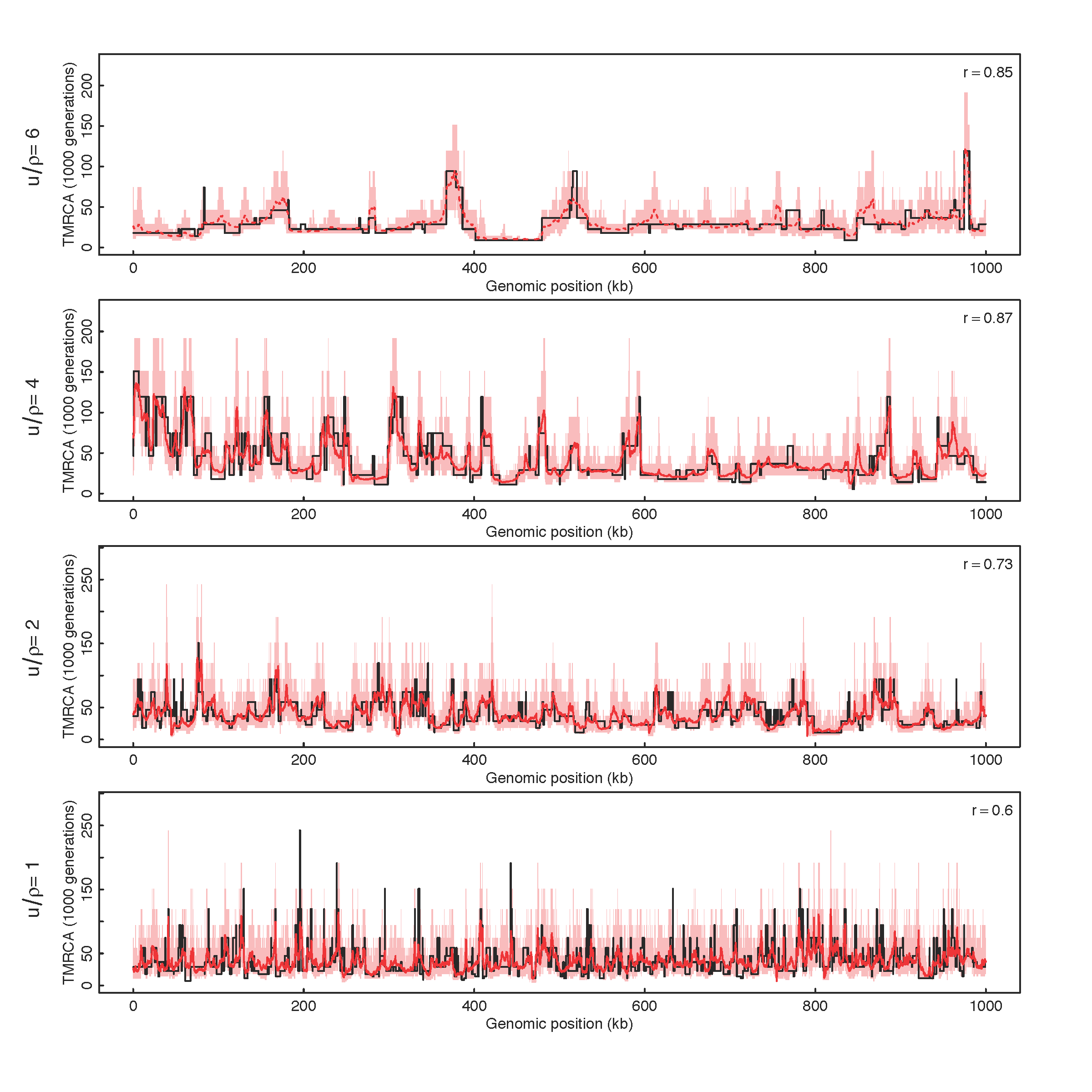

Supplement: Figure S5 — Recovery of TMRCA along simulated sequences for various values of . This figure is the same as Figure 4B except that it shows results for four diffrent values of the mutation-to-recombination rate ratio, ranging from (bottom panel) to (top panel). Each panel represents one randomly selected simulated data set. Pearson's correlation coefficients () for true vs. estimated TMRCAs across all local trees are shown in the top right corner of each panel. As expected, the quality of the estimates generally improves with , but this example suggests there is limited improvement above . (PNG) [file pgen.1004342.s005.png]

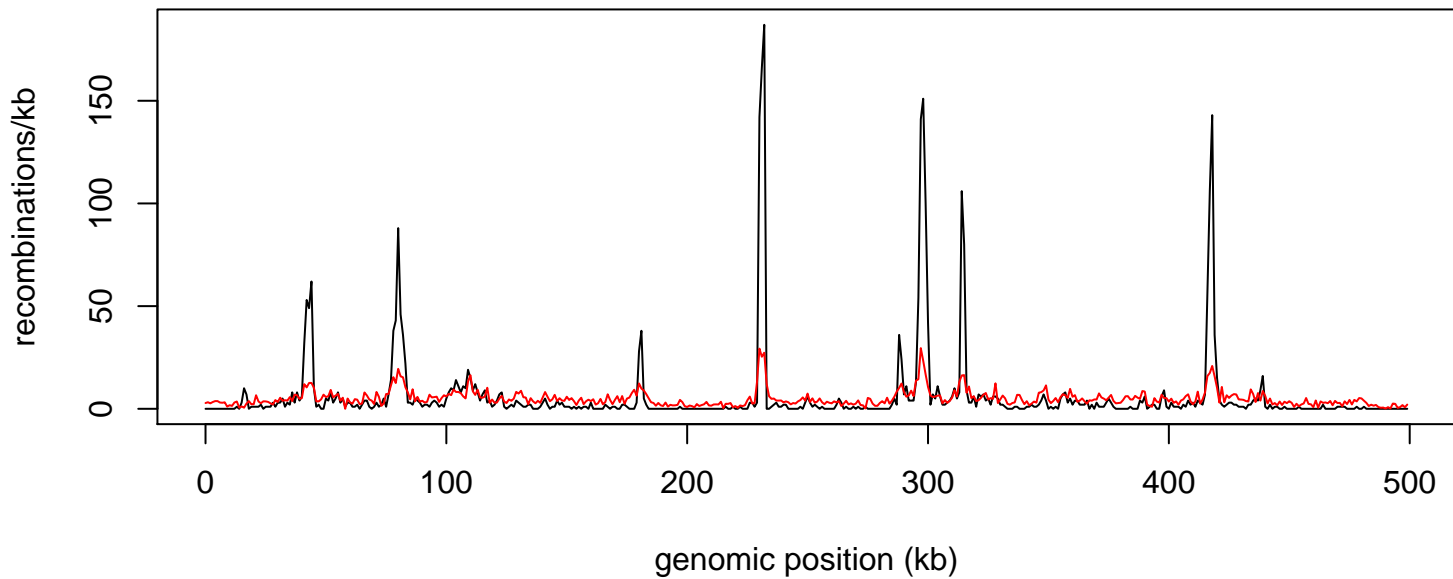

Supplement: Figure S6 — Recovery of recombination rates from simulated data. We simulated an alignment of 100 sequences with and , allowing for variable recombination rates based on estimates along the human genome. Despite the assumption in the prior of a constant recombination rate of , the posterior mean estimate of the average number of recombinations in a 1 kb sliding window (red line) correlates well with the true recombination rates used during simulation (black line). Notice that recombination hotspots are clearly identifiable by peaks in the inferred rates but the magnitudes of these peaks are dampened by the use of a uniform prior. Only recombinations that produced changes in tree topology (the class that is detectable by our methods) were considered for the plot of the true recombination rate. (PDF) [file pgen.1004342.s006.pdf]

$u/p=6$

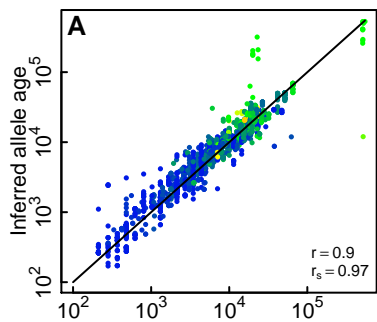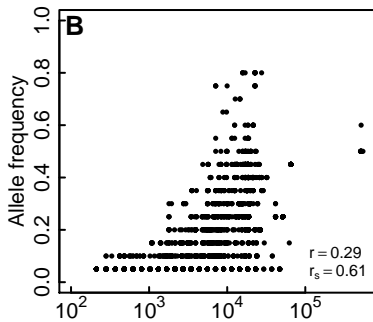

$u/p=4$

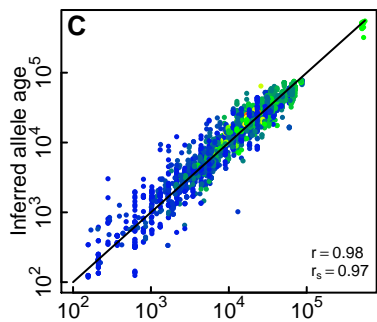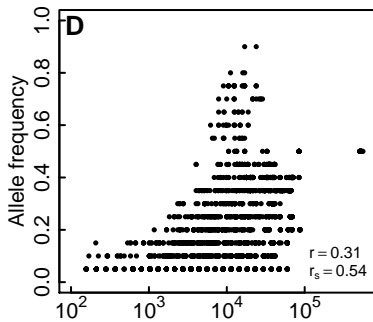

$u/p=2$

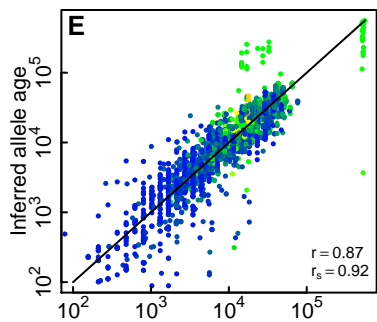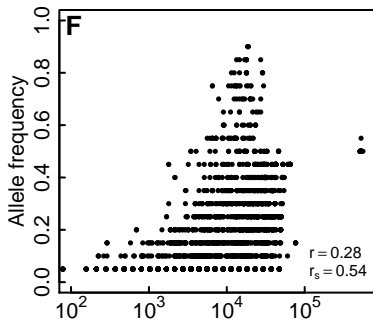

$u/p=1$

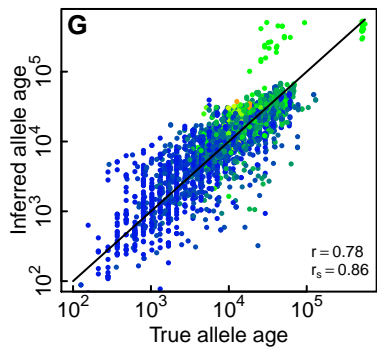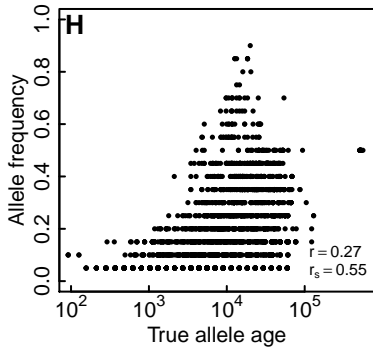

Supplement: Figure S7 — Estimating ages of derived alleles in simulated data. (A,C,E,G) Inferred allele age correlates well with true allele age according to both Pearson's () and Spearman's rank () correlation coefficients. Correlation is strongest for high mutation/recombination rate ratios. Ages were estimated by calculating the midpoint of the branch on which the mutation was inferred to occur, under an infinite sites model, and averaging across sample from the posterior distribution. Points are colored on a spectrum from blue to green in proportion to derived allele frequencies. (B,D,F,H) Allele frequency has significantly lower correlation with true allele age, implying that the ARG will enable much better estimates of allele age than allele frequencies alone. Ages are measured in generations before the present. Our standard simulated data sets were used (Methods). (PDF) [file pgen.1004342.s007.pdf]

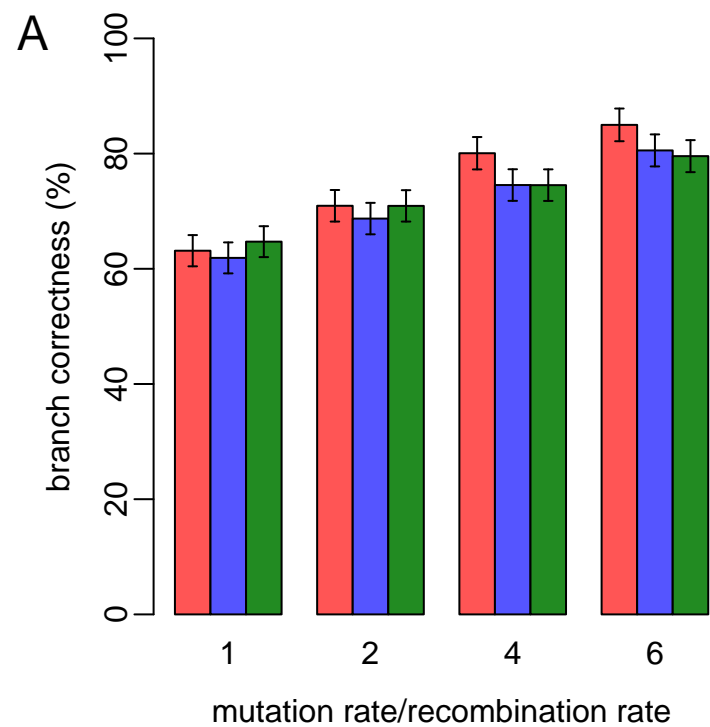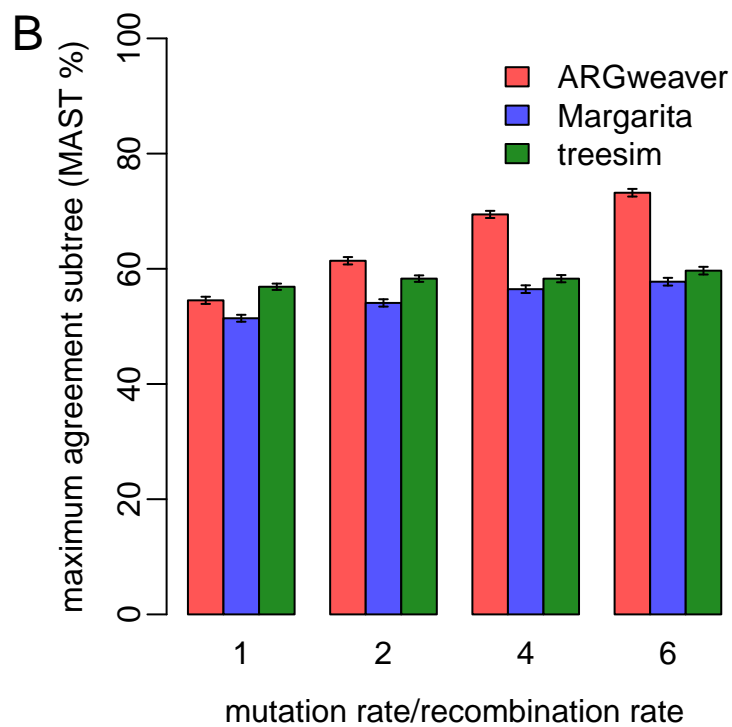

Supplement: Figure S8 — Recovery of local tree topologies. Sequences were simulated under the coalescent-with-recombination using our standard parameters (Methods), ARGs were inferred using ARGweaver, then 100 equally spaced local trees were extracted from the sampled ARGs. The topologies of these trees were compared with the true trees generated during simulation at corresponding positions in the alignment. We compared ARGweaver with the heuristic programs Margarita [34] and treesim using two measures: (A) branch correctness (one minus the normalized Robinson-Foulds (RF) distance [108]) and (B) Maximum Agreement Subtree (MAST) percentages (the size of the largest leaf-set such that induced subtrees are topologically equivalent, expressed as a percentage of the total number of leaves), across a range of mutation to recombination rate ratios (). In both (A) and (B), error bars reflect one standard error assuming independence of 100 local trees ×10 simulation replicates. (PDF) [file pgen.1004342.s008.pdf]

Probability of correctness

1.0  
0.8  
0.6  
0.4  
0.2  
0.0

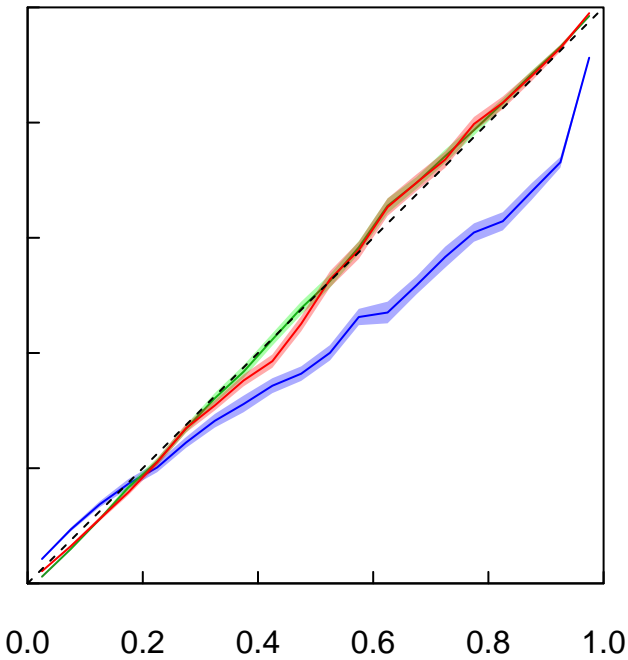

Supplement: Figure S9 — Local tree branch posterior probabilities inferred by ARGweaver accurately reflect their probability of correctness. The branch posterior probabilities found by ARGweaver (red) and treesim (green) more accurately reflect the probability of the branch being correct than the frequency at which Margarita (blue) infers a branch. For each method, branches were binned by their posterior probability (windows of 5%) and compared against their frequency of branch correctness. Shaded regions represent the 95% binomial confidence interval. This plot is based on our standard simulated data set with . Posterior probabilities for ARGweaver are based on 1000 samples from the Markov chain, and the probabilities for Margarita and treesim reflect 100 independent samples. (PDF) [file pgen.1004342.s009.pdf]

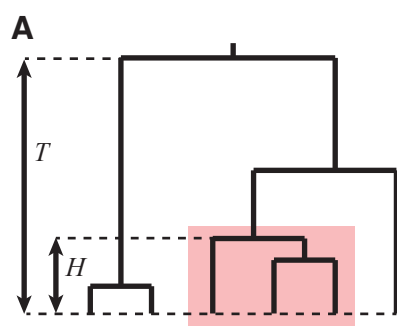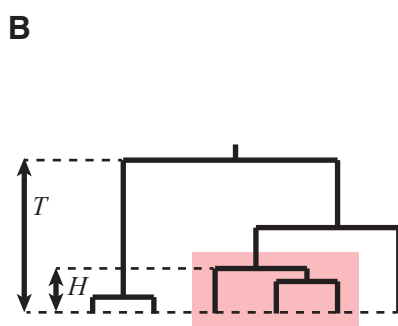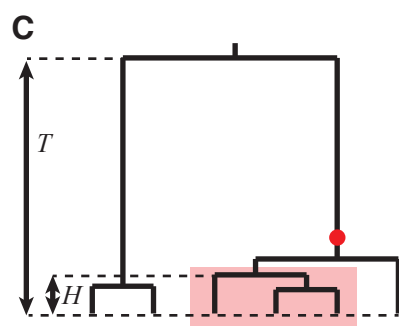

Supplement: Figure S10 — Illustration of relative TMRCA halflife (RTH). Expected genealogies under (A) neutral drift, (B) background selection, and (C) a partial selective sweep. In each panel, the arrows to the left indicate the complete TMRCA () and the “half TMRCA” (), that is, the minimum time required for half of all lineages to find a single most recent common ancestor. The relative TMRCA halflife (RTH) is defined by the ratio . Because background selection (B) should primarily reduce the overall rate of coalescence, in a manner more or less homogeneous with respect to time, it is expected to have little effect on the RTH. Partial sweeps (C), however, will tend to produce a “burst” of coalescent events following a causal mutation (red circle), leading to reduced values of . Nevertheless, because some lineages escape the sweep, the full TMRCA is likely to remain similar to its value under neutrality. As a result, the RTH will be reduced. (PDF) [file pgen.1004342.s010.pdf]

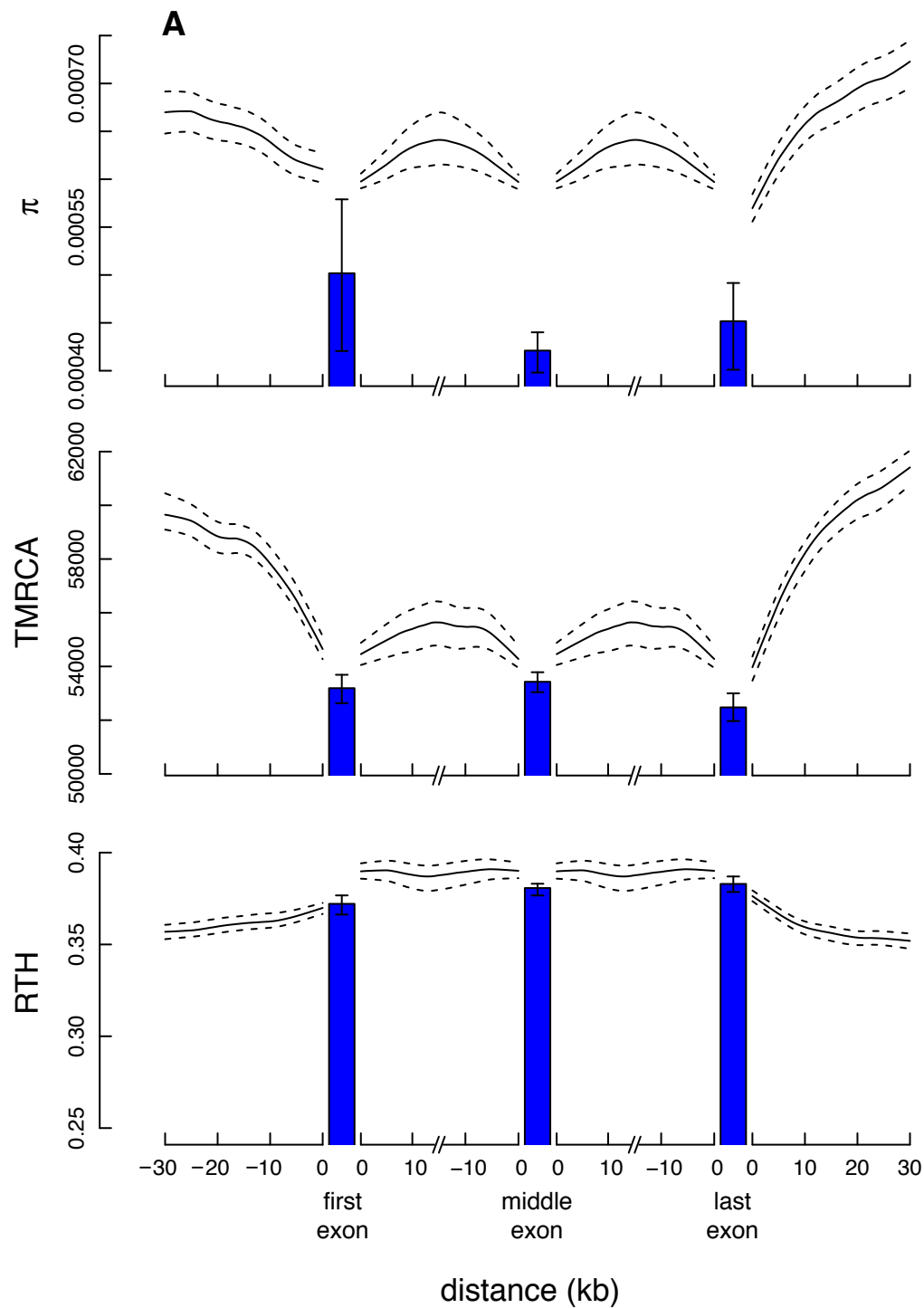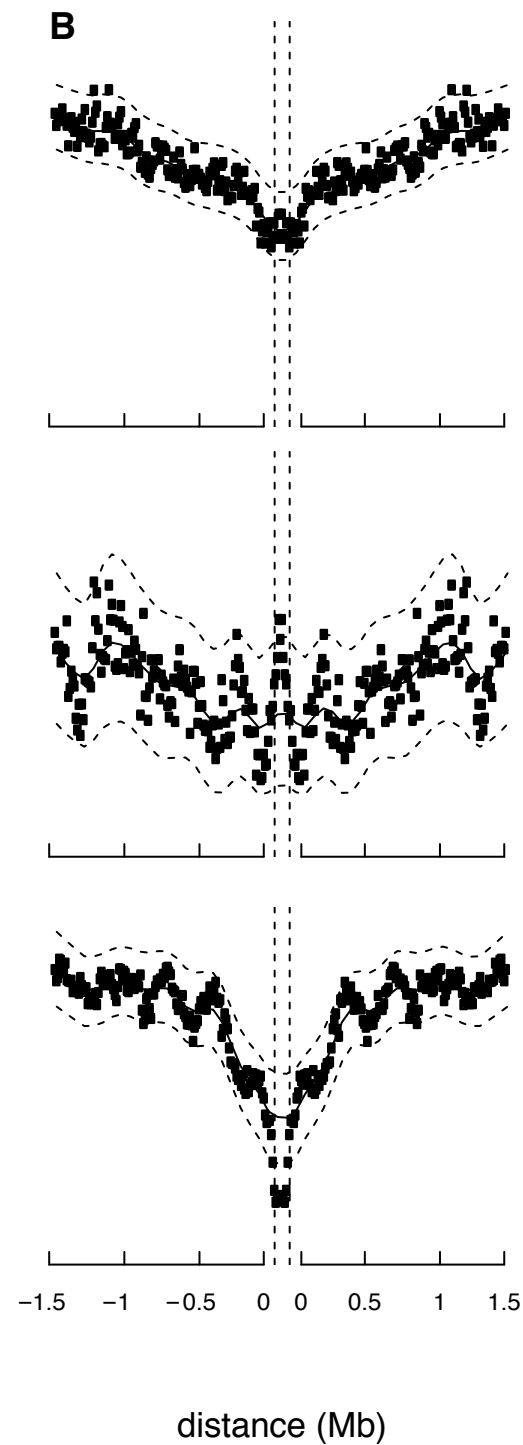

Supplement: Figure S11 — Measures of genetic variation near protein-coding genes and partial selective sweeps for African populations. This figures is identical to Figure 5 except that it shows results for 17 African individuals or 34 haploid genomies (from the YRI, MKK, and LWK populations). Panel (A) is based on the same 17,845 protein-coding genes as in Figure 5A. Panel (B) is based on 271 100-kb regions predicted to have undergone partial selective sweeps in the YRI population based on the iHS statistic [72]. (PDF) [file pgen.1004342.s011.pdf]

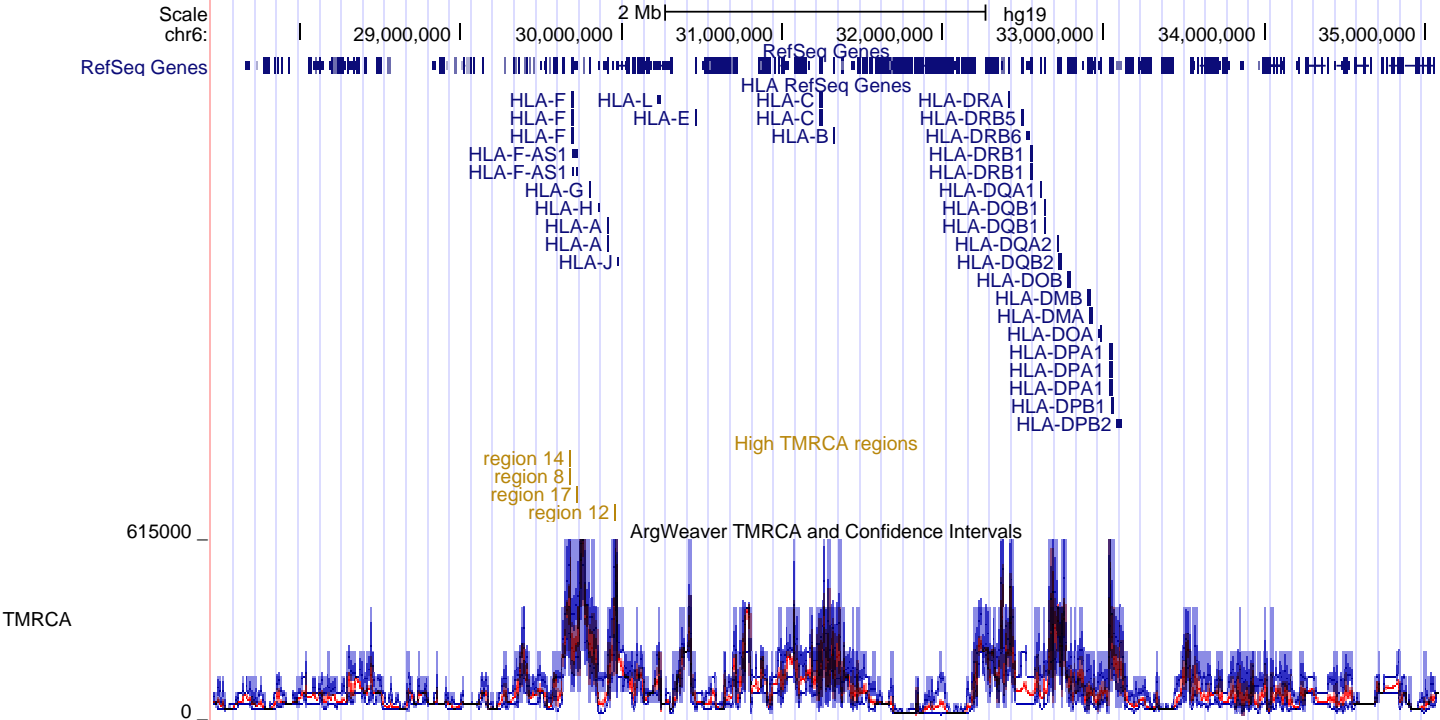

Supplement: Figure S12 — Time to most recent common ancestry (TMRCA) in the human leukocyte antigen (HLA) region. Genome browser track displaying the sitewise time to most recent common ancestry (TMRCA) estimated by ARGweaver based on the Complete Genomics individual human genome sequence data (track is available at http://genome-mirror.bscb.cornell.edu, assembly hg19). The human leukocyte antigen (HLA) region on human chromosome 6 contains many genomic intervals with extremely elevated expected TMRCAs, including four of the top 20 10-kb regions in the genome (highlighted here in gold; see descriptions in Table 2). The red line indicates the posterior mean of the TMRCA (estimated by averaging over the sampled local trees) and the blue lines above and below indicate a Bayesian 95% credible interval. (PDF) [file pgen.1004342.s012.pdf]

$F_n(x)$

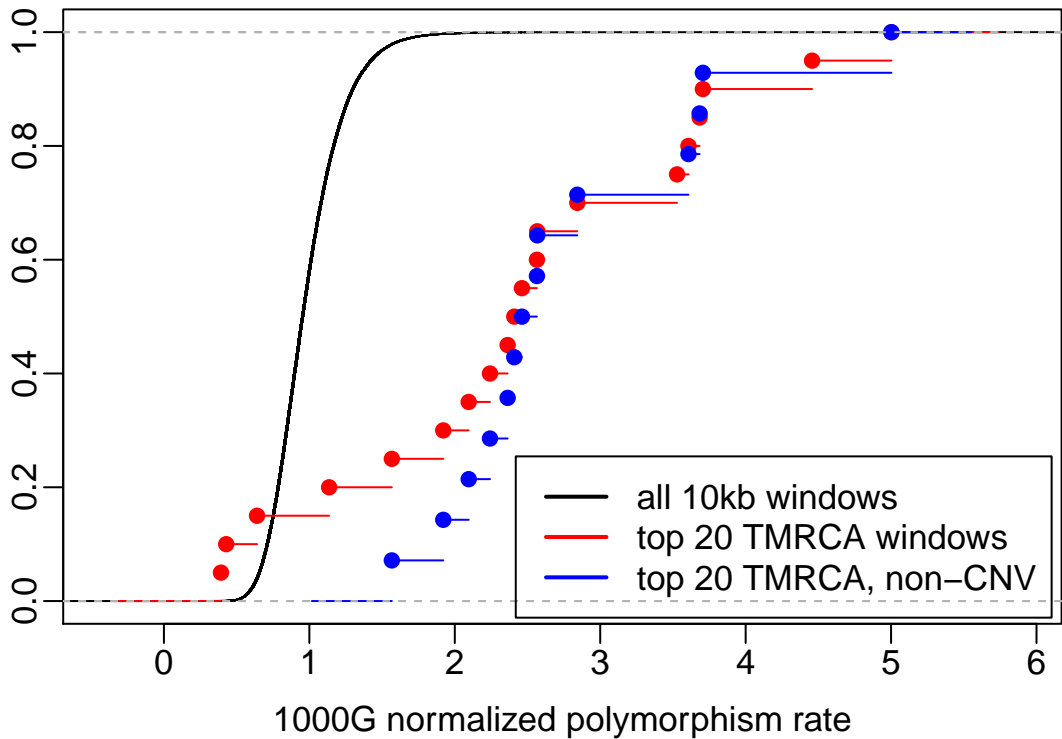

Supplement: Figure S13 — Mutation-rate normalized polymorphism rates in the 1000 Genomes Phase 1 data are elevated in the top twenty 10 kb regions by TMRCA. Shown are cumulative distribution fuctions for normalized polymorphism rates (computed as for Table 2) in all 10 kb windows across the human genome (black), the top twenty regions shown in Table 2 (red), and the fifteen regions not identified as possible CNVs in Table 2 (blue). (PDF) [file pgen.1004342.s013.pdf]

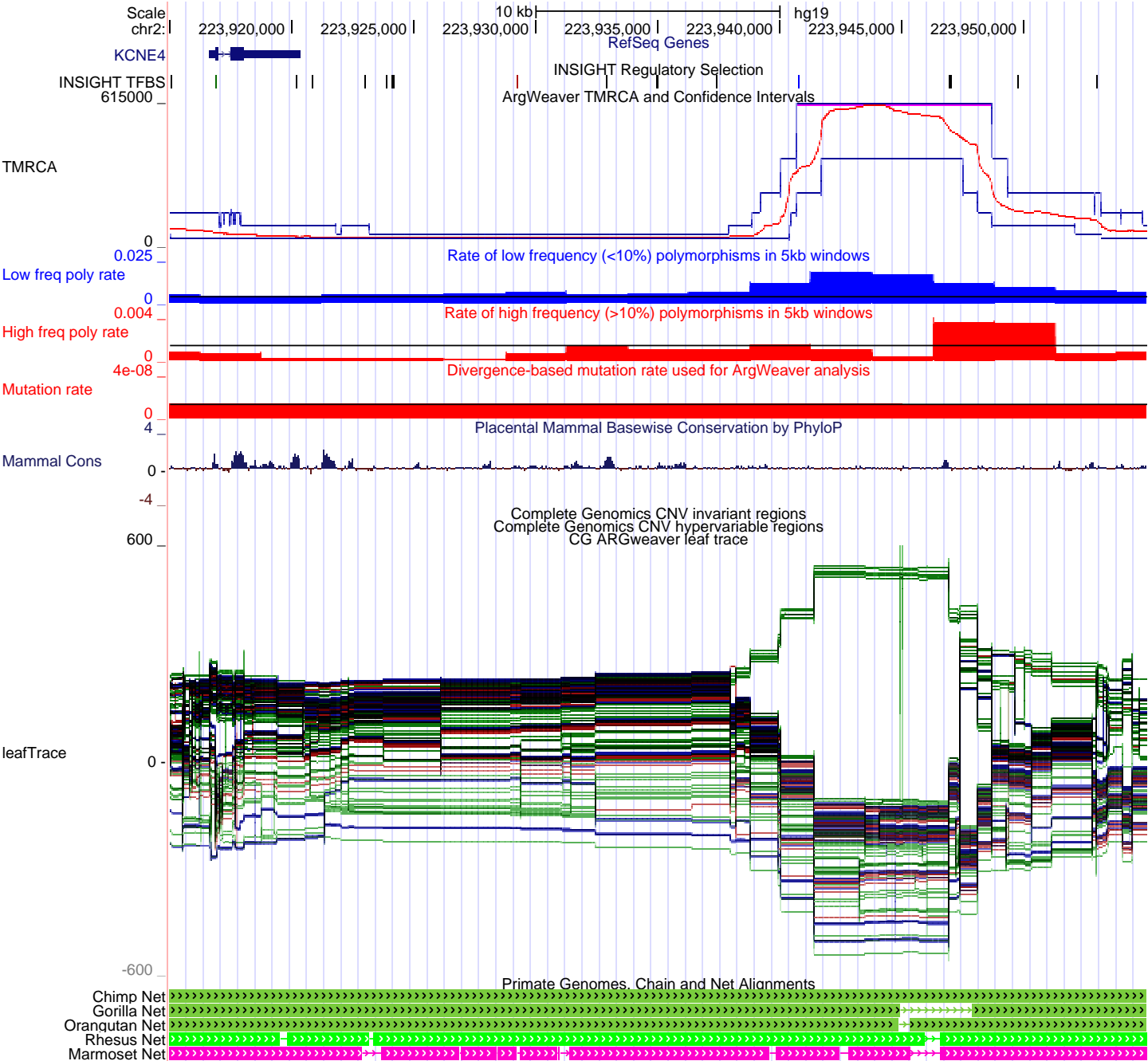

Supplement: Figure S14 — ARGweaver tracks near KCNE4. Shown is a ∼10-kb peak in the estimated TMRCA about 20 kb downstream of the KCNE4 gene (shown in blue), which encodes a potassium voltage-gated channel strongly expressed in the embryo and adult uterus. The peak overlaps two ChIP-seq-supported transcription factor binding sites analyzed by Arbiza et al. [109] (“INSIGHT Regulatory Selection” track). The four tracks below the TMRCA plot show that the region in question displays elevated rates of both low-frequency (<10% derived allele frequency; shown in blue) and high-frequency (≥10%; shown in red) polymorphisms in the Complete Genomics data set, despite that divergence-based estimates of the mutation rate are at or below the genome-wide average (average values are indicated by horizontal black lines). ARGweaver explains these observations by inferring one of the deepest average TMRCAs in the human genome (#5 in Table 2). Additional tracks show no indication of copy number variation or recent duplications in this region. The leaf trace indicates that the signal for a deep TMRCA is driven by individuals from African populations (shown in green; the European and East Asian populations are shown in blue and red, respectively), suggesting that this region may contain ancient haplotypes specific to Africa. (PDF) [file pgen.1004342.s014.pdf]

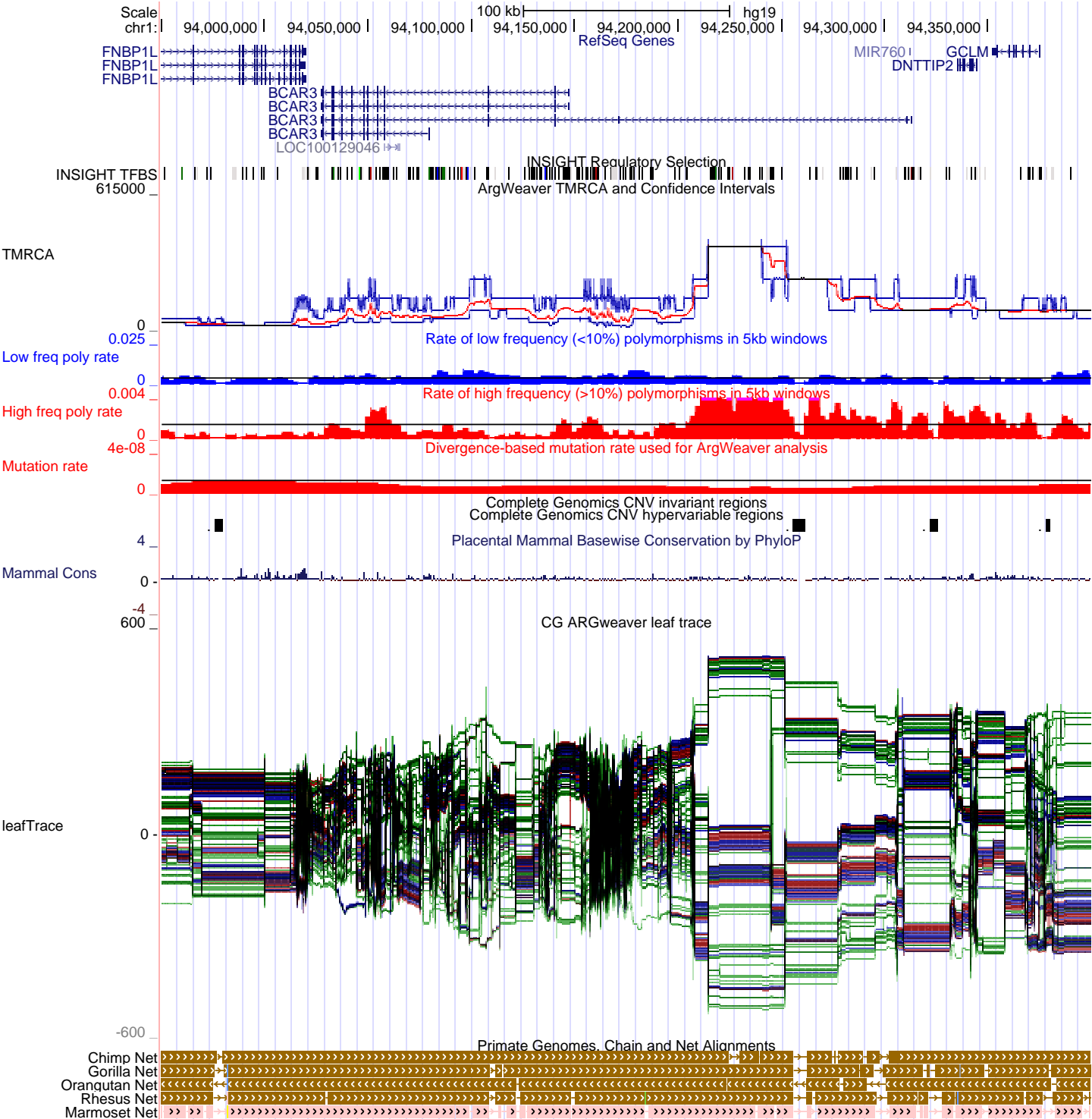

Supplement: Figure S15 — ARGweaver tracks near BCAR3. Shown is a large region of elevated TMRCA in an intron of the BCAR3 gene, which is involved in the development of anti-estrogen resistance in breast cancer. One 10-kb segment of this region has an average expected TMRCA of 377,017 generations, or approximately 9.4 My (#9 in Table 2). As in the previous example, this region shows elevated polymorphism rates but average or below-average mutation rates and overlaps ChIP-seq-supported transcription factor binding sites (INSIGHT track) [109]. Again, the regions of extreme TMRCA do not seem to be explained by copy number variation or recent duplications. In this case, however, the leaf trace demonstrates that the ancient haplotypes are distributed across all three major population groups (African = green, European = blue, East Asian = red). (PDF) [file pgen.1004342.s015.pdf]

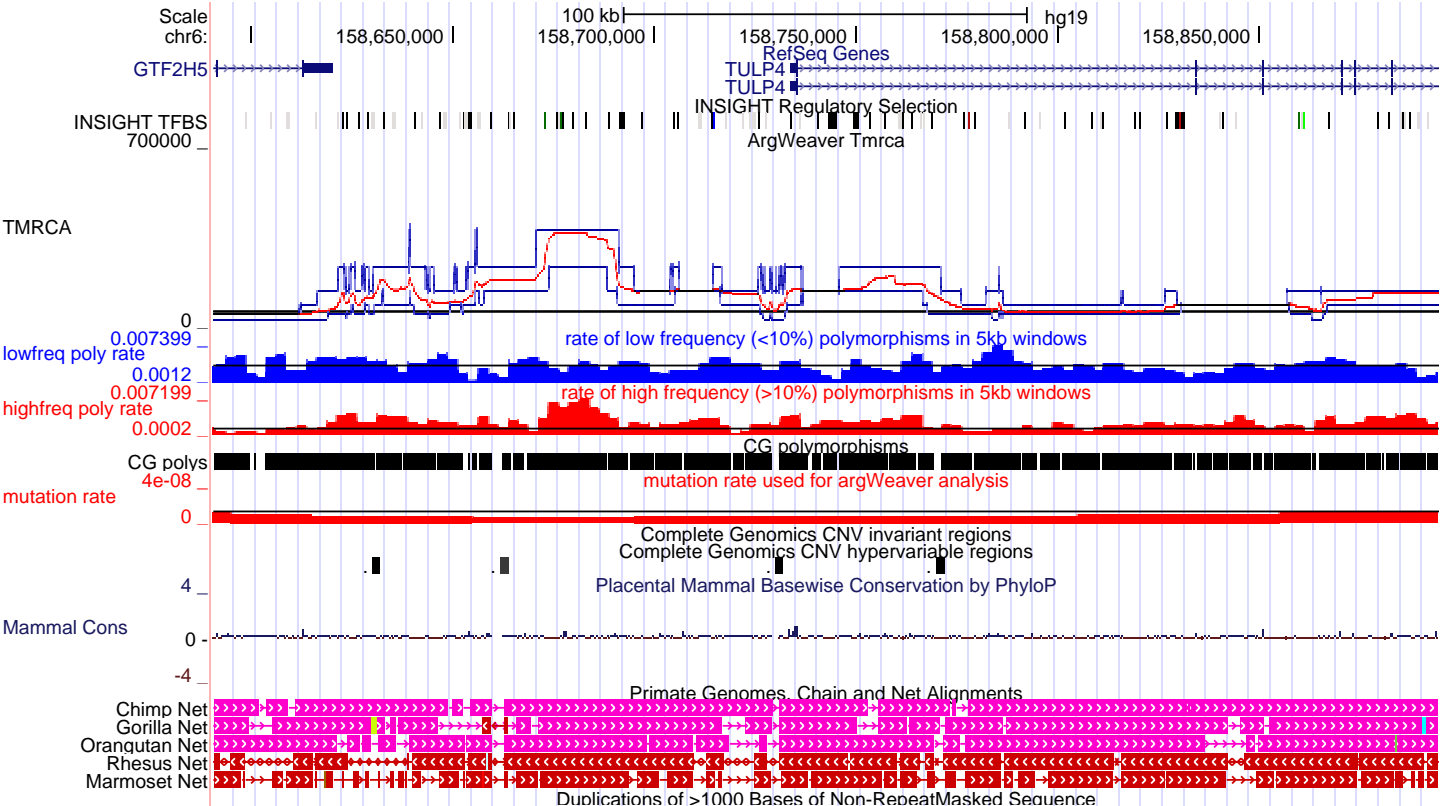

Supplement: Figure S16 — ARGweaver tracks near TULP4. Another large region of elevated TMRCA upstream of the TULP4 gene, which is thought to be involved in ubiquitination and proteosomal degradation and has a possible association with cleft lip. One 10-kb segment has an average expected TMRCA of 345,382 generations (8.6 My; #16 in Table 2). As in the previous two examples, this region has elevated polymorphism rates but not mutation rates, overlaps ChIP-seq-supported transcription factor binding sites (INSIGHT track), and does not seem to be an artifact of copy number variation or recent duplications. (PDF) [file pgen.1004342.s016.pdf]

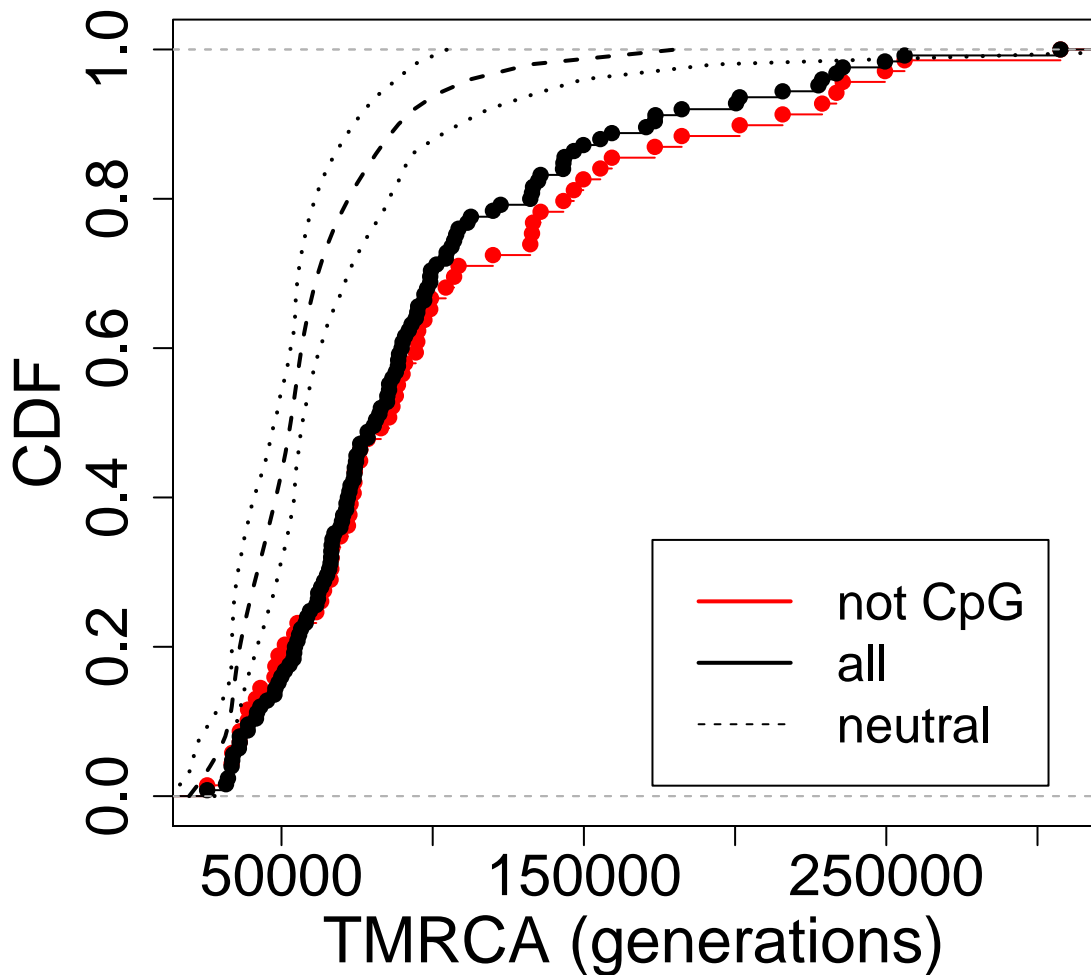

Supplement: Figure S17 — Distribution of TMRCAs in regions predicted to be under balancing selection. Cumulative distribution functions (CDFs) are shown for the 125 regions identified by Leffler et al. [77] based on segregating haplotypes shared between humans and chimpanzees (black circles), the subset of 69 loci containing no shared polymorphisms in CpG dinucleotides (red circles) and a collection of 69 putatively neutral regions having the same length distribution. Neutral regions consisted of noncoding regions from which known genes, binding sites, and conserved elements had been removed (see [109]). Notice the pronounced shift toward larger TMRCAs in the regions predicted to be under balancing selection, and a slightly more pronounced shift for the subset not containing CpGs (which are less likely to have undergone parallel mutations on both lineages). TMRCAs are measured in generations, as in all other figures and tables. (PDF) [file pgen.1004342.s017.pdf]

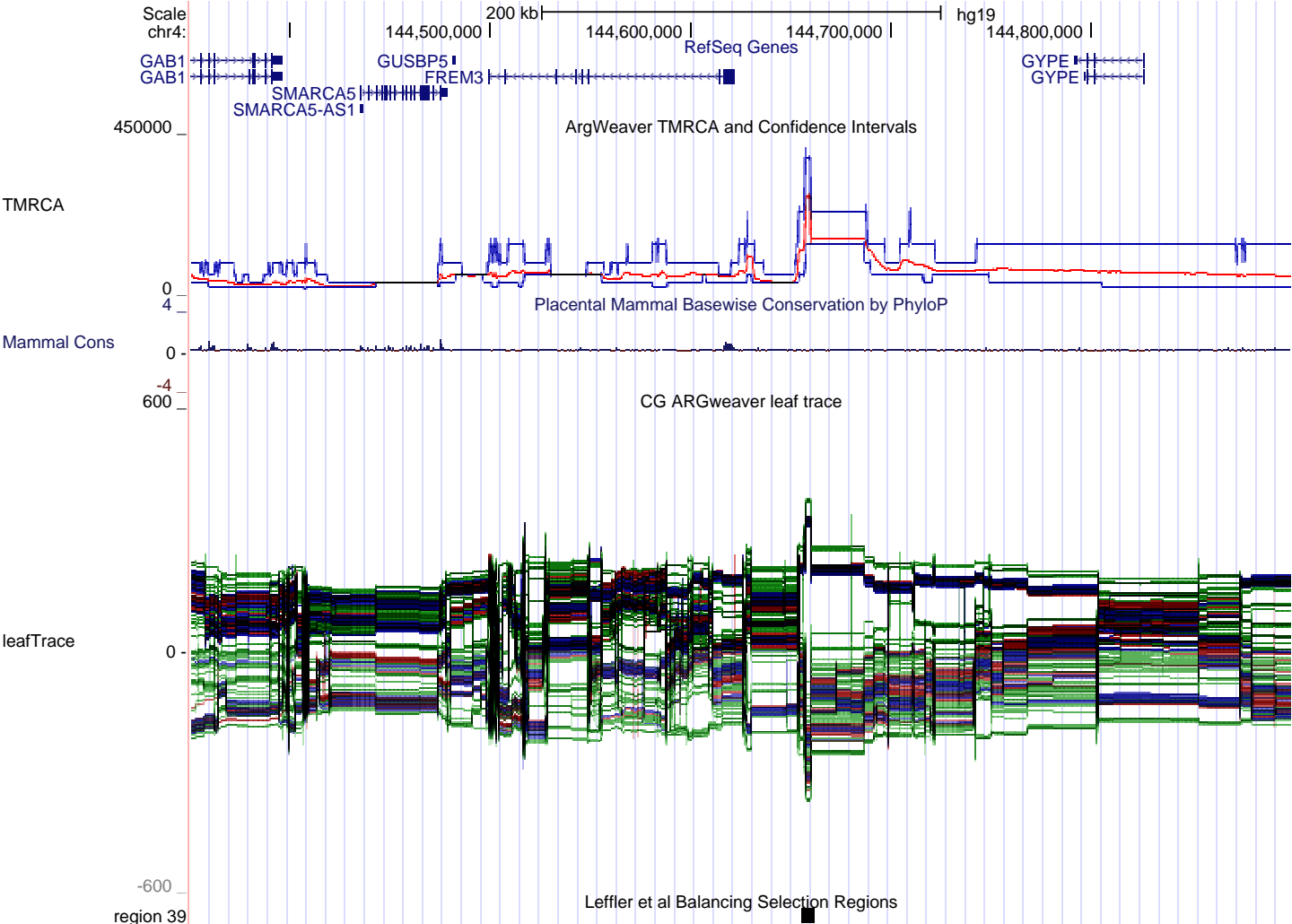

Supplement: Figure S18 — ARGweaver tracks near locus containing segregating haplotypes shared in humans and chimpanzees. Elevated TMRCA corresponding to a region identified by Leffler et al. [77] between the FREM3 and GYPE genes (#11 in Table 3; see black square in track at bottom). The shared polymorphisms in this region are in strong linkage disequilibrium with eQTLs for GYPE, a paralog of GYPA, which may be under balancing selection. The leaf trace indicates that the ancient haplotypes are shared across major human population groups (African = green, European = blue, East Asian = red). (PDF) [file pgen.1004342.s018.pdf]

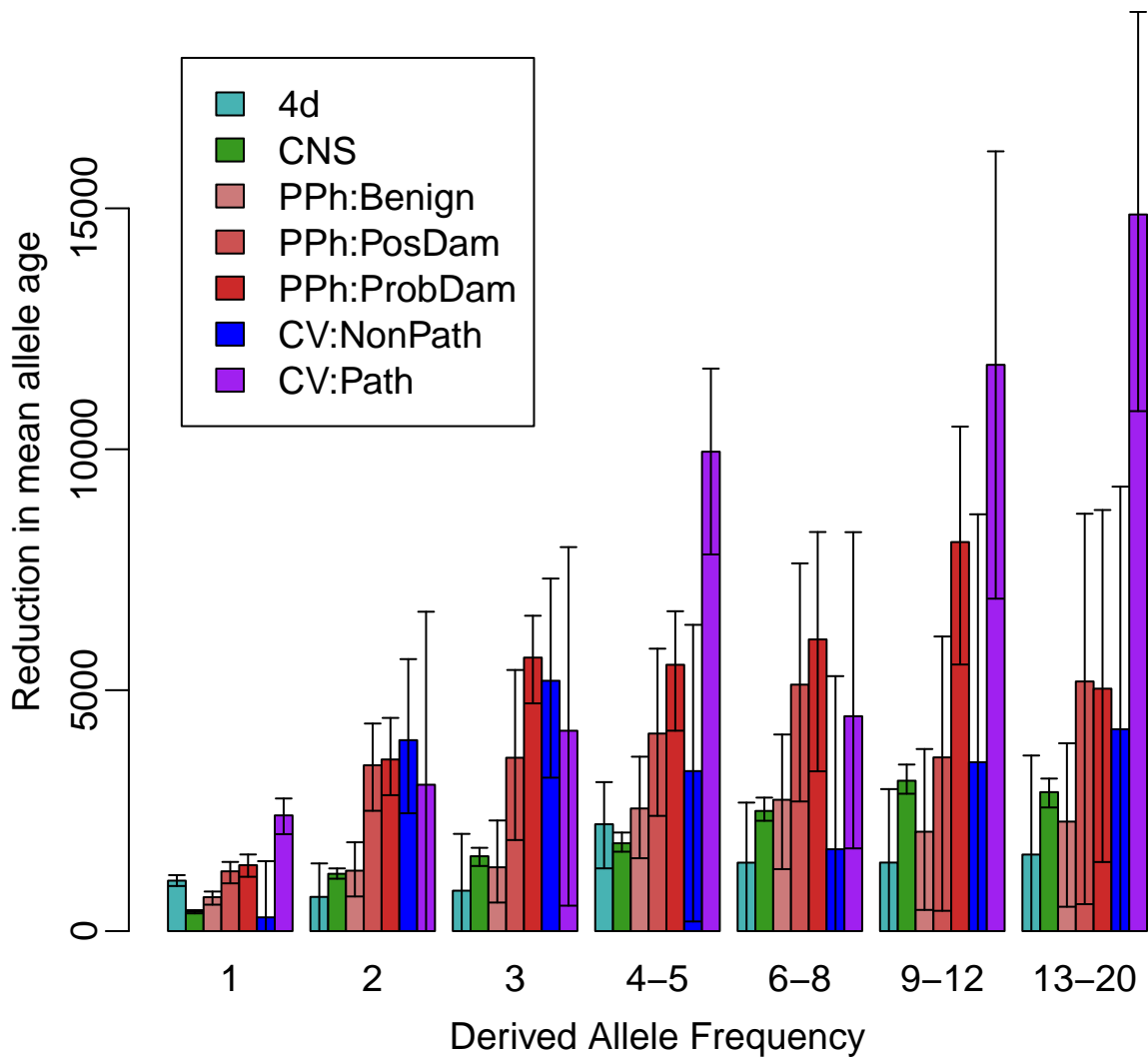

Supplement: Figure S19 — Reduction in mean allele age as a function of annotation class and derived allele frequency. This figure shows the same information as Figure 6B, but instead of plotting absolute values of the estimated allele ages, it plots the estimated reduction in allele age relative to neutrality, which is defined as the differences between the estimated age for each annotation type and the estimate for the corresponding neutral class (in generations). This representation shows clearly that the reduction in allele age increases with allele frequency much more rapidly for annotation classes under strong selection than for those under weak selection. The contrast between the nearly neutral classes (4d, PPh:Benign, CV:NonPath) and the strongly selected classes (PPh:ProbDam, CV:Path) is particularly striking. This difference can be understood as follows. Reductions in allele age at nearly neutral sites will primarily be a consequence of selection at linked sites, which, to a first approximation, will decrease the local effective population size. This will have the effect of approximately re-scaling allele ages by a constant factor across all ages, making the reduction in age roughly proportional to the absolute age. Mutations under stronger direct selection, by contrast, will spend disproportionally less time at higher frequencies, making their reductions in age at high frequencies disproportionally larger than those for nearly neutral mutations (see [79]). This effect will occur even in the absence of dominance (), but it could be exascerbated by dominance, which will tend to make low-frequency alleles invisible to direct selection. In any case, this plot shows that selection from linked sites can produce comparable, or even larger, reductions in age than direct selection at low allele frequencies, but at high frequencies, direct selection tends to dominate in age reduction. (PDF) [file pgen.1004342.s019.pdf]

**A**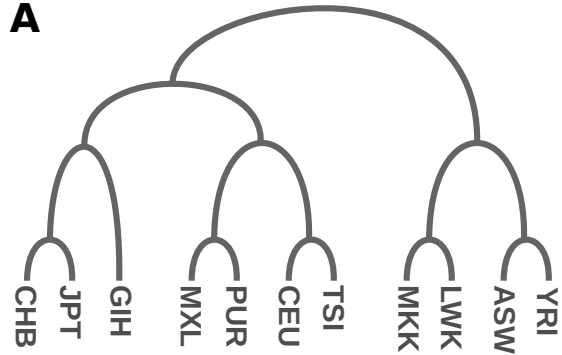**B**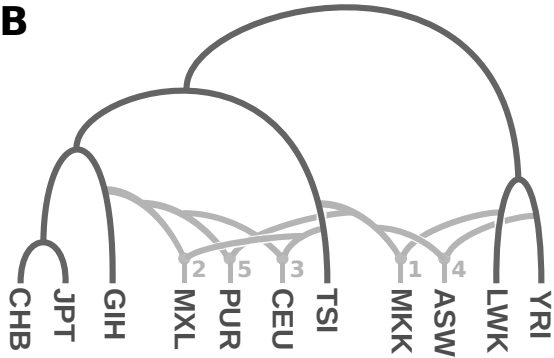

Supplement: Figure S20 — Human population phylogenies inferred from sampled ancestral recombination graphs. Phylogenetic networks for the eleven populations represented in the Complete Genomics data set were reconstructed using the PhyloNet program [90], [91]. As input to PhyloNet, we used 2,304 local trees extracted from the ARG at approximately 1 Mb intervals, with one randomly sampled chromosome per population (see Text S1). (A) Population phylogeny inferred in the absence of hybridization/admixture, showing the expected primary relationships among populations. (B) Population networks inferred when between one and five hybridization nodes are allowed. Populations inferred to be admixed are indicated by gray lines and the inferred hybridization nodes are shown as gray circles. Numbers indicate the order in which these nodes appear. For example, when one hybridization node is allowed, the MKK population is inferred to be admixed, and when two are allowed, the MXL population is also inferred to be admixed. The inferred network is consistent with other recent studies in many respects, but PhyloNet is unable to reconstruct the precise topology of the complex subnetwork consisting of the GIH, MXL, PUR, CEU, and TSI populations (see Text S1). Population names follow the convention used by the HapMap 3 and 1000 Genomes projects: CHB = Han Chinese in Beijing, China; JPT = Japanese in Tokyo, Japan; GIH = Gujarati Indians in Houston, Texas; MXL = Mexican ancestry in Los Angeles, California; PUR = Puerto Ricans in Puerto Rico; CEU = Utah residents with Northern and Western European ancestry from the Centre d'Etude de Polymorphisme Humain (CEPH) collection; TSI = Toscani in Italy; MKK = Maasai in Kinyawa, Kenya; LWK = Luhya in Webuye, Kenya; ASW = African ancestry in Southwest USA; YRI = Yoruba in Ibadan, Nigeria. (PDF) [file pgen.1004342.s020.pdf]

A

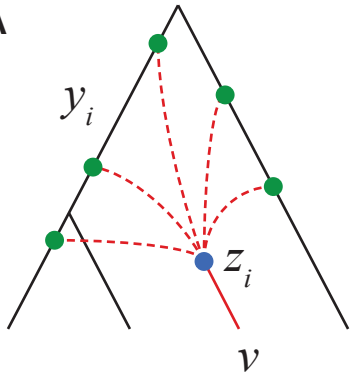

B

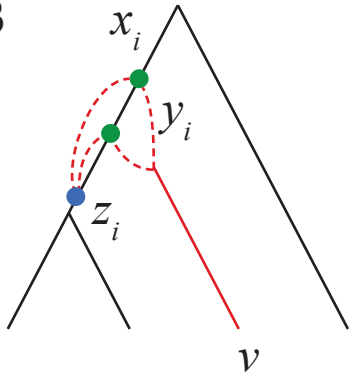

Supplement: Figure S21 — Cases for new recombination given re-coalescence point . (A) In the main case, the recombination (blue point) occurs on the branch that is being threaded into the ARG (; shown in red). After a recombination on this branch, a re-coalescence can occur at any point (green points) in the local tree such that is at least as old as . Therefore, when enumerating the possible consistent with a given , one must consider all points on branch at least as recent as . This set is denoted in the text. (B) There is an additional special case to consider when branch coalesces to the same branches of at positions and , that is, when . In this case, it is possible that the recombination (blue point) occurs not on the new branch but on (black branch) at a time point no older than the re-coalescence time (green points). A recombination of this kind will leave an identical signature to the symmetric case of a recombination on in the same time interval followed by a re-coalescence of to . Therefore, when enumerating the possible consistent with a given such that , one must also consider the set consisting of all on such that is at least as recent as . Notice that, in both (A) and (B), the tree excluding is unchanged by all recombination and coalescence scenarios under consideration, i.e., (black branches). (PDF) [file pgen.1004342.s021.pdf]
