# Supplementary material for: Genome-Wide Inference of Ancestral Recombination Graphs
Source: PLoS Genet. 2014 May 15;10(5):e1004342. doi: 10.1371/journal.pgen.1004342 (PMC4022496; doi:10.1371/journal.pgen.1004342)
Supplement: Text S1 — Supplementary methods and analyses. (PDF) [file pgen.1004342.s022.pdf]

Genome-wide inference of ancestral recombination graphs

Supplementary Information: Text S1

Matthew D. Rasmussen, Melissa J. Hubisz, Ilan Gronau, Adam Siepel

Department of Biological Statistics and Computational Biology, Cornell University, Ithaca, New York 14853, USA

Supplementary Methods

Calculation of transition probabilities

The general formula for the transition probabilities of the HMM (equation 20) can be simplified and its evaluation can be made more efficient by recognizing several distinct scenarios for the joint configuration of the previous local tree T^n_{i-1}, the current local tree T^n_i, and the recombination R^n_i. We will consider two main cases, corresponding to the presence ($R^{n-1}_i \neq \emptyset$) and absence ($R^{n-1}_i = \emptyset$) of "old" (previously sampled) recombinations, respectively (see Figure S1-1). In addition, we will consider three subcases of each of these main cases. Throughout this section, we will use the notation $y_{i-1} = (x_{i-1}, t_{i-1})$ and $y_i = (x_i, t_i)$ to indicate the previous and current coalescence points for the resampled branch, respectively, with each x_i indicating a branch and each t_i a time point. We will assume y_{i-1} is indexed by l and y_i by m, and we will assume their time points are indexed by a and b, respectively (i.e., $t_{i-1} = s_a$, $t_i = s_b$). In addition, $z_i = (w_i, u_i)$ will denote a new recombination between positions $i-1$ and i, with w_i indicating a branch and u_i a time point in T^n_{i-1}. We will use v to indicate the new branch that is being threaded into the ARG. We will assume the single sequence threading operation, so v must be an external branch, but the subtree threading setting is very similar (as discussed in later sections). We will also use the notation $S(u)$ to indicate the index of the time point associated with a node u in a local tree.

Major Case #1: No Old Recombinations

Let us first consider the case in which there are no old recombinations, $R^{n-1}_i = \emptyset$. Because of the restriction of at most one recombination per genomic position, this is the only case in which a new recombination is possible ($z_i \neq \emptyset$). The three subcases for the transition probabilities are as follows:

1. **Recoalescence to different branches:** $x_{i-1} \neq x_i$. In this subcase, the recombination must have occurred on the new branch, $w_i = v$, as discussed in the section entitled "Sampling a Recombination Threading" in the main text. In addition, the time of the recombination, u_i, must range between 0 and the minimum of t_{i-1} and t_i. Thus,

$$\begin{aligned} a^{i-1}_{l,m} &= \sum_{z_i} P(\bar{R}^{n-1}_i, z_i \mid \bar{T}^{n-1}_{i-1}, y_{i-1} = l)\, P(\bar{T}^{n-1}_i, y_i = m \mid \bar{R}^{n-1}_i, z_i, \bar{T}^{n-1}_{i-1}, y_{i-1} = l) \\ &= \sum_{k=0}^{\min(a,b)} P(\bar{R}^{n-1}_i = \emptyset, z_i = (v, s_k) \mid \bar{T}^{n-1}_{i-1}, y_{i-1} = l)\, P(\bar{T}^{n-1}_i, y_i = m \mid \bar{R}^{n-1}_i = \emptyset, z_i = (v, s_k), \bar{T}^{n-1}_{i-1}, y_{i-1} = l) \end{aligned} \quad \text{(S1)}$$

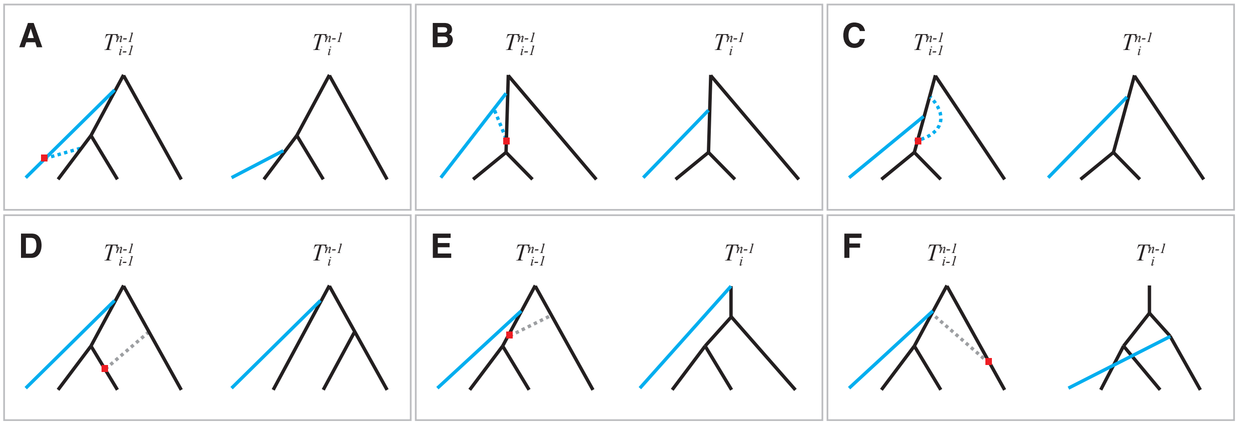

Supplementary Text Figure S1-1: **Examples of thread transitions.** (A) When no old recombinations are present ($R_i^{n-1} = \emptyset$) the thread state y_i can change if a new recombination z_i added. If this recombination is added to the new branch v (blue), that branch may re-coalesce anywhere else in the local tree. Alternatively, the new recombination can be placed on the branch associated with the previous thread state, x_{i-1}, in which case the recoalescence must occur on the new branch v (B) or on branch x_{i-1} (C). When an old recombination is present ($R_i^{n-1} \neq \emptyset$), the associated SPR operation often does not affect the thread location. However, it can cause the thread state to change its branch (D) or its time (E) in a deterministic manner. (F) If the recoalescence point is the same as the thread point, the thread state can change to recombination-bearing branch w_i and any time within the interval between the recombination and recoalescence times, $t_i \in [u_i, t]$. The thread state can also change to the branch above the recoalescence point and keep the same time (not shown).

where, for simplicity, we drop the explicit conditioning on the model parameters ρ and N. Notice that this sum has no more than $K+1$ terms.

As in the general case, the first term in equation S1 is given by equation 5 and the second term by equation 13 from the main text. However, these equations simplify in this case. Because we have assumed that $R_i^{n-1} = \emptyset$ and we can assume that $z_i = (v, s_k)$ represents a valid recombination, we can write the following in place of equation 5,

$$P(\bar{R}_i^{n-1} = \emptyset, z_i = (v, s_k) \mid \bar{T}_{i-1}^{n-1}, y_{i-1} = l) = \frac{1}{A'_k} \cdot \frac{B_k \Delta s_k}{C} \cdot \left[1 - \exp(-\rho |T_{i-1}^n|)\right], \tag{S2}$$

where,

$$A'_k = \begin{cases} 2 & \text{if } s_k = s_r \\ A_k & \text{otherwise} \end{cases} \tag{S3}$$

and all other terms are as defined for equation 5. Similarly, equation 13 simplifies in this case to,

$$P(\bar{T}_i^{n-1}, y_i = m \mid \bar{R}_i^{n-1} = \emptyset, z_i = (v, s_k), \bar{T}_{i-1}^{n-1}, y_{i-1} = l) = \frac{1}{A_b^{(-v)}} P(s_b \mid v, s_k, T_{i-1}^n) \tag{S4}$$

where $P(s_b \mid v, s_k, T_{i-1}^n)$ is given by equations 10–12.

2. **Recoalescence to same branch at different times:** $x_{i-1} = x_i$, $t_{i-1} \neq t_i$. In this case, the recombination may have occurred either on the new branch v or on the recoalescence branch x_i. If the recombination occurred on

branch v, then, as above, its time index can range between 0 and the minimum of a and b (the indices of t_{i-1} and t_i, respectively). If it occurred on branch x_i, then its time index can range between the time index at which x_i came into existence, which is given by $S(x_i)$, and the minimum of t_{i-1} and t_i. Thus,

$$
\begin{aligned}
a_{l,m}^{i-1} &= \sum_{z_i} P(\bar{R}_i^{n-1}, z_i \mid \bar{T}_{i-1}^{n-1}, y_{i-1} = l)\, P(\bar{T}_i^{n-1}, y_i = m \mid \bar{R}_i^{n-1}, z_i, \bar{T}_{i-1}^{n-1}, y_{i-1} = l) \\
&= \sum_{k=0}^{\min(a,b)} P(\bar{R}_i^{n-1} = \emptyset, z_i = (v, s_k) \mid \bar{T}_{i-1}^{n-1}, y_{i-1} = l)\, P(\bar{T}_i^{n-1}, y_i = m \mid \bar{R}_i^{n-1} = \emptyset, z_i = (v, s_k), \bar{T}_{i-1}^{n-1}, y_{i-1} = l) \\
&\quad + \sum_{k=S(x_i)}^{\min(a,b)} P(\bar{R}_i^{n-1} = \emptyset, z_i = (x_i, s_k) \mid \bar{T}_{i-1}^{n-1}, y_{i-1} = l)\, P(\bar{T}_i^{n-1}, y_i = m \mid \bar{R}_i^{n-1} = \emptyset, z_i = (x_i, s_k), \bar{T}_{i-1}^{n-1}, y_{i-1} = l)
\end{aligned}
\tag{S5}
$$

As in case (1), the first term in each of these sums is given by equation S2 and the second term is given by equation S4. Each of these sums also has no more than $K+1$ terms.

3. **Recoalescence to same branch at same time:** $x_{i-1} = x_i$, $t_{i-1} = t_i$. This case is similar to the previous one, except that it must also allow for the possibility of no recombination between positions $i-1$ and i ($z_i = \emptyset$). Thus,

$$
\begin{aligned}
a_{l,l}^{i-1} &= \sum_{z_i} P(\bar{R}_i^{n-1}, z_i \mid \bar{T}_{i-1}^{n-1}, y_{i-1} = l)\, P(\bar{T}_i^{n-1}, y_i = l \mid \bar{R}_i^{n-1}, z_i, \bar{T}_{i-1}^{n-1}, y_{i-1} = l) \\
&= \exp\left(-\rho |T_{i-1}^{n}|\right) \\
&\quad + \sum_{k=0}^{a} P(\bar{R}_i^{n-1} = \emptyset, z_i = (v, s_k) \mid \bar{T}_{i-1}^{n-1}, y_{i-1} = l)\, P(\bar{T}_i^{n-1}, y_i = m \mid \bar{R}_i^{n-1} = \emptyset, z_i = (v, s_k), \bar{T}_{i-1}^{n-1}, y_{i-1} = l) \\
&\quad + \sum_{k=S(x_i)}^{a} P(\bar{R}_i^{n-1} = \emptyset, z_i = (x_i, s_k) \mid \bar{T}_{i-1}^{n-1}, y_{i-1} = l)\, P(\bar{T}_i^{n-1}, y_i = m \mid \bar{R}_i^{n-1} = \emptyset, z_i = (x_i, s_k), \bar{T}_{i-1}^{n-1}, y_{i-1} = l)
\end{aligned}
\tag{S6}
$$

Major Case #2: Old Recombinations

The other major case to consider is when a recombination is already given, $R_i^{n-1} = (w_i, s_k) \neq \emptyset$. Our modeling assumptions prohibit a new recombination in this case, so it must be true that $z_i = \emptyset$. Thus,

$$
\begin{aligned}
a_{l,m}^{i-1} &= \sum_{z_i} P(\bar{R}_i^{n-1} = (w_i, s_k), z_i \mid \bar{T}_{i-1}^{n-1}, y_{i-1} = l)\, P(\bar{T}_i^{n-1}, y_i = m \mid \bar{R}_i^{n-1} = (w_i, s_k), z_i, \bar{T}_{i-1}^{n-1}, y_{i-1} = l) \\
&= P(\bar{R}_i^{n-1} = (w_i, s_k), z_i = \emptyset \mid \bar{T}_{i-1}^{n-1}, y_{i-1} = l)\, P(\bar{T}_i^{n-1}, y_i = m \mid \bar{R}_i^{n-1} = (w_i, s_k), z_i = \emptyset, \bar{T}_{i-1}^{n-1}, y_{i-1} = l)
\end{aligned}
\tag{S7}
$$

Because we can assume in this setting that $\bar{R}_i^{n-1}$ represents a valid recombination, the first term has a form similar to that of equation S2, that is,

$$
P(\bar{T}_i^{n-1}, y_i = m \mid \bar{R}_i^{n-1} = (w_i, s_k), z_i = \emptyset, \bar{T}_{i-1}^{n-1}, y_{i-1} = l) = \frac{1}{A'_k} \cdot \frac{B_k \Delta s_k}{C} \cdot \left[1 - \exp(-\rho |T_{i-1}^{n}|)\right], \tag{S8}
$$

where A'_k is given by equation S3 and all other terms are as defined for equation 5. Similarly, the second term has a form similar to that of equation S4,

$$P(\bar{T}_i^{n-1}, y_i = m \mid \bar{R}_i^{n-1} = (w_i, s_k), z_i = \emptyset, \bar{T}_{i-1}^{n-1}, y_{i-1} = l) = \frac{1}{A_b^{(-w_i)}} P(s_b \mid w_i, s_k, T_{i-1}^n). \tag{S9}$$

The calculation of these transition probabilities can be further simplified by considering three subcases. In defining these cases, we use the notation (x'_i, t'_i) to indicate the recoalescence point associated with the old recombination $R_i^{n-1} = (w_i, u_i)$. Notice that, in the case of an old recombination, this is not the same as the state $y_i = (x_i, t_i)$, which represents the new coalescence point for branch v (not branch w_i). Here, the time index b corresponds to the recoalescence time t'_i, that is, $s_b = t'_i$.

1. **Deterministic case.** If the previous state $y_{i-1} = (x_{i-1}, t_{i-1})$ does not equal either the recoalescence point (x'_i, t'_i) or the recombination point $z_i = (w_i, s_k)$, then the transition process is completely deterministic, meaning that there is only one transition with non-zero probability. A series of well-defined rules identifies the state y_i that must follow y_{i-1} (Figure S1-2). Note that, because the HMM is unnormalized, the probability of the permitted state transition will generally not be equal to one.

2. **Recombination-point case.** If the previous state y_{i-1} equals the recombination point $z_i = (w_i, s_k)$, then the recombination point can be either above the new branch v or below it. If the recombination is above branch v, then the new state must be the same as the old one, $y_i = y_{i-1}$. If, on the other hand, the recombination is below branch v, then branch x_{i-1} "escapes" and the new branch v must coalesce up higher in the tree (see Figure S1-2 for a similar calculation).

3. **Recoalescence-point case.** If the previous state equals the recoalescence point, $(x_{i-1}, t_{i-1}) = (x'_i, t'_i)$, we must consider the possibility that the recombining branch w_i recoalesces at (x'_i, t'_i) as well as the possibility that w_i recoalesces at any location along the new branch v. The reason is that all such scenarios allow T_i^{n-1} to have the same configuration after removal of v. Note that this case is always distinct from case (2) because the recoalescence cannot be on the same branch as the recombination (this would imply a *bubble*, which are not allowed in the SMC process). The destination states y_i that are relevant for this scenario are $y_i = (x_{i-1}, t_i)$, $y_i = (p(w_i), t'_i)$, and $y_i = (w_i, t_l)$, where $p(w_i)$ is the new parent of w_i (and x_{i-1}), and $t_l \leq t'_i$ is any valid recoalescing point along v. Since the recombination point $R_i^{n-1} = (w_i, s_k)$ is not the same as the state $y_{i-1} = (x_{i-1}, t_{i-1})$ in this case, there is no ambiguity about the location of the recombination.

Dynamic programming

A limiting step in the calculation of the transition probabilities described in the previous sections is the evaluation of equation 10. A naive evaluation of this equation requires $O(K)$ time, resulting in a running time of $O(K^2)$ for the calculation of individual transition probabilities.

```
function get_deterministic_transition((x_{i-1},t_{i-1}), (w_i,s_k), (x'_i,t'_i), mapping) {
  if ((x_{i-1},t_{i-1}) == (x'_i,t'_i) || (x_{i-1},t_{i-1}) == (w_i,s_k)) {
    // not a deterministic case
    return NULL
  } else if (x_{i-1} ≠ w_i) {
    // SPR only removes a subset of descendents, if any
    // trace up from remaining leaf to find correct new state
    disrupt = false
    if (x_{i-1}.is_leaf()) {
      // SPR cannot disrupt leaf branch
      x_i = x_{i-1}
    } else {
      if (w_i == x_{i-1}.children[0]) {
        // left child is not disrupted
        x_i = mapping[x_{i-1}.children[0]]
        disrupt = true
      } else if (w_i == x_{i-1}.children[1]) {
        // right child is not disrupted
        x_i = mapping[x_{i-1}.children[1]]
        disrupt = true
      } else {
        // x_i is not disrupted
        x_i = mapping[x_{i-1}]
      }
    }
    // optionally walk up, if coalescence occurs under thread
    if ((x == x_{i-1} && t'_i < t_{i-1}) || (x'_i == x_i && t'_i < t_{i-1}) || (disrupt && x == x_i && t <= t_{i-1}))
      x_i = x_i.parent
    return (x_i,t_{i-1})
  } else {
    // SPR is on same branch as thread
    if (s_k > t_{i-1}) {
      // thread moves with SPR subtree
      return (mapping[w_i], t_{i-1})
    } else {
      // SPR subtree moves out from underneath thread, therefore the new
      // branch coalesces with the branch above the subtree
      parent = w_i.parent
      t_i = parent.age
      other = w_i.sibling()
      x_i = mapping[other]
      if (other == x'_i) x_i = x_i.parent
      return (x_i, t_i)
    }
  }
}
```

Supplementary Text Figure S1-2: **Deterministic rules.** When a previous recombination is given ($R_i^{n-1} = (w_i, s_k) \neq \emptyset$), most transitions are deterministic and can be determined by the set of rules shown here. The basic idea of this procedure is that the recombination R_i^{n-1} and recoalescence $y_i = (x_i, t_i)$ together define a subtree pruning and regrafting (SPR) operation on the local tree T_{i-1}^{n} such that the coalescence point y_i of the new branch v is unambiguous given y_{i-1} and the other available information. The variable `mapping` maps nodes in T_{i-1}^{n-1} to equivalent nodes in T_i^{n-1}.

Let us re-express equation 10 as,

$$P(s_j \mid w, s_k, T^n_{i-1}, \Theta) = \exp\left[-C_{k,j-2} - \frac{B^{(-w)}_{j-1}\,\Delta s_{j-1,j-\frac{1}{2}}}{2N_{j-1}}\right] \times \left[1 - \exp\left(-\frac{B^{(-w)}_{j-1}\,\Delta s_{j-\frac{1}{2},j}}{2N_{j-1}} - \frac{B^{(-w)}_{j}\,\Delta s_{j,j+\frac{1}{2}}}{2N_j}\right)\right], \quad \text{(S10)}$$

where,

$$C_{k,m} = \sum_{l=k}^{m} \frac{B^{(-w)}_l \Delta s_l}{2N_l}. \quad \text{(S11)}$$

Notice that,

$$\begin{aligned} C_{k,m} &= \sum_{l=k}^{m} \frac{B^{(-w)}_l \Delta s_l}{2N_l} \\ &= \left(\sum_{l=0}^{m} \frac{B^{(-w)}_l \Delta s_l}{2N_l}\right) - \left(\sum_{l=0}^{k-1} \frac{B^{(-w)}_l \Delta s_l}{2N_l}\right) \\ &= C_{0,m} - C_{0,k-1}. \end{aligned} \quad \text{(S12)}$$

The values of the form $C_{0,m}$ can be computed recursively in a preprocessing step for $m = 0, \ldots, K$, as follows:

$$C_{0,m} = \begin{cases} 0 & m = 0 \\ C_{0,m-1} + \frac{B^{(-w)}_m \Delta s_m}{2N_m} & 1 \le m \le K. \end{cases} \quad \text{(S13)}$$

Thus, after preprocessing, the evaluation of equation 10 can be accomplished in constant time and the calculation of individual transition probabilities can be accomplished in $O(K)$ time.

Further optimization of forward algorithm

Implemented in a direct manner, the forward algorithm would require $O(Ln^2K^2)$ time. However, by taking advantage of redundancies in the transition probabilities we can reduce this running time to $O(LnK^2)$. The approach used here is similar to that used by Paul et al. [1].

Recall that the forward algorithm computes a table of values of the form,

$$\begin{aligned} f_{i,m} =& P(\boldsymbol{T}^{n-1}_{1:i}, \boldsymbol{R}^{n-1}_{1:i}, \boldsymbol{T}^{n}_{1:i}, y_i = m \mid \Theta) \\ =& b^i_m(D^n_i) \sum_l f_{i-1,l}\, a^{i-1}_{l,m}, \end{aligned} \quad \text{(S14)}$$

where $b^i_m(D^n_i)$ is the emission probability for state m and alignment column i, and $a^{i-1}_{l,m}$ is the transition probability from state l to state m at position $i-1$ (see section entitled “Stochastic Traceback” in main text for complete details).

Again, let the state variable y_i be defined by a branch x_i and a time t_i. In addition, let $\mathcal{C}(x_i, j)$ be the index for state $y_i = (x_i, t_i = s_j)$, where s_j is the jth time point. These indices define the orders of the rows and columns of the transition matrix for position i, denoted $\boldsymbol{A}_i$. Now, observe that, for many choices of consecutive states $l = \mathcal{C}(x_{i-1}, j)$

and $m = \mathcal{C}(x_i, k)$, the transition probability $a^i_{l,m}$ does not depend on x_{i-1} and x_i but only depends on the time indices j and k. In particular, if $x_{i-1} \neq x_i$, the transition probability is independent of the identity of the branches, because of the symmetry among all branches at each time point in the coalescent model.

These symmetries mean that the true dimensionality of $\boldsymbol{A}_i$ is considerably reduced. To exploit this reduced dimensionality, let us define a reduced transition matrix $\boldsymbol{A}'_i = \{a'^i_{j,k}\}$ indexed by the time points (i.e., $0 \leq j \leq K$ and $0 \leq k \leq K$), such that $a'^i_{j,k}$ gives the transition probability from time point j to time point k assuming that $x_i \neq x_{i+1}$. We can now rewrite the recurrence in the forward algorithm as follows, assuming that k is the time point associated with index m:

$$\begin{aligned}
f_{i,m} =& b^i_m(D^n_i) \sum_l f_{i-1,l} a^{i-1}_{l,m} \\
=& b^i_m(D^n_i) \left[\left(\sum_{l:x_i \neq x_{i-1}} f_{i-1,l} a^{i-1}_{l,m} \right) + \left(\sum_{l:x_i = x_{i-1}} f_{i-1,l} a^{i-1}_{l,m} \right) \right] \\
=& b^i_m(D^n_i) \left[\left(\sum_{j=0}^{K} \sum_{l:l=\mathcal{C}(x_{i-1},j), x_i \neq x_{i-1}} f_{i-1,l} a'^{i-1}_{j,k} \right) + \left(\sum_{l:x_i = x_{i-1}} f_{i-1,l} a^{i-1}_{l,m} \right) \right] \\
=& b^i_m(D^n_i) \left[\left(\sum_{j=0}^{K} a'^{i-1}_{j,k} \sum_{l:l=\mathcal{C}(x_{i-1},j), x_i \neq x_{i-1}} f_{i-1,l} \right) + \left(\sum_{l:x_i = x_{i-1}} f_{i-1,l} a^{i-1}_{l,m} \right) \right] \\
=& b^i_m(D^n_i) \left[\left(\sum_{j=0}^{K} a'^{i-1}_{j,k} f'_{i-1,j} \right) + \left(\sum_{l:x_i = x_{i-1}} f_{i-1,l} a^{i-1}_{l,m} \right) \right]
\end{aligned} \tag{S15}$$

where

$$\begin{aligned}
f'_{i-1,j} &= \sum_{l:l=\mathcal{C}(x_{i-1},j), x_i \neq x_{i-1}} f_{i-1,l} \\
&= \left(\sum_{l:l=\mathcal{C}(x_{i-1},j)} f_{i-1,l} \right) - f_{i-1,\mathcal{C}(x_i,j)},
\end{aligned} \tag{S16}$$

and $f_{i-1,\mathcal{C}(x_i,j)}$ is zero if the state (x_i, s_j) does not exist.

Notice that the $f'_{i-1,j}$ terms can be reused in calculating $f_{i,m}$ for all values of m. As a result, computing each column of the forward table takes $O(nK^2)$ time instead of $O(n^2K^2)$ time, and the total running time of the algorithm is reduced to $O(LnK^2)$.

Subtree sampling

In this section, we outline our strategy for *subtree sampling* (i.e., resampling of internal branches in the local trees) in greater detail. As described in the main text, subtree sampling is needed to enable efficient mixing of the MCMC sampler with more than few sequences. Unlike the single-sequence threading operation, subtree sampling allows the "deep structure" of the ARG to be perturbed in a reasonably efficient manner.

For each local tree T^n_i, imagine that one of the internal branches v is removed, thus producing two trees: a *main*

tree $T_i^{M,n}$ and a *subtree* $T_i^{S,n}$. The main tree has the same root node as the original full tree T_i^n and has a basal branch that extends to the maximum time s_K. The subtree $T_i^{S,n}$ has v as its root node and does not have a basal branch. In this setting, the effect of the recoalescence operation is to allow the partial local tree $(T_i^{M,n}, T_i^{S,n})$ to be reconnected into a full local tree. This is accomplished by allowing introducing a lineage leading to v and allowing it to recoalesce with the main tree. Notice that this is a direct generalization of the single sequence recoalescence operation. In that case, v is required to be a leaf node, but in the general case, it is allowed to be any node (other than the root) in the local tree. As in the single sequence case, we can denote the recoalescence point at site i by $y_i = (x_i, t_i)$.

Let the age of the subtree root v be s_q. Notice that the structures of the main tree and the subtree below age s_q do not affect the coalescence rate of the new branch v. Thus, resampling the coalescence point for the internal branch is essentially the same as resampling the coalescence point for an external branch. The only restriction is that the recoalesce point must be at least as old as s_q. This operation can be used within any local block having a single local tree, i.e., for which $\boldsymbol{T}_i^n = \boldsymbol{T}_j^n$ for all i and j. However, a new problem arises in the case in which the local trees differ across sites, as discussed in the next section.

Resampling internal branches across multiple local blocks

Internal branches can also be resampled across multiple local blocks. This involves removing one branch from each tree in $\boldsymbol{T}^n$ to create a list of main trees $\boldsymbol{T}^{M,n}$ and subtrees $\boldsymbol{T}^{S,n}$. A coalescence threading $\boldsymbol{Y}$ can then be sampled to define how each subtree recoalesces to the corresponding main tree, thereby defining a new collection of complete local trees $\boldsymbol{T}^n$.

The problem is that a poor choice of a series of internal branches to remove and resample can result in a highly constrained threading distribution. To see why this is true, imagine that the selected series of internal branches is such that the branch for each local block is completely unrelated (e.g., in a different subtree of the full phylogeny) to the previous one. In this case, if a new recombination is sampled within a local block during the subtree threading operation, that recombination will have to be "undone" by the end of the local block to allow the new local tree for that block to be reconciled with the main tree and subtree for the next block. Thus, any "move" in ARG space must involve tightly coordinated sequences of recombinations that cancel one another out, in a sense. Because such sequences will be difficult to find, there will be a strong tendency to simply resample the previous threading, and the sampler will not mix well.

The solution to this problem is to select sequences of internal branches that are in some way mutually "compatible," so that these constraints on the reconciliation of local trees across blocks are relaxed. It turns out that it is sufficient to select sequences of branches such that adjacent branches in the sequence *share ancestry*, as defined below.

In order to identify such sequences we use an auxiliary data structure called a *branch graph* (Figure S1-3). The branch graph $\mathcal{B}$ is derived from the local trees $\boldsymbol{T}^n$ and recombinations $\boldsymbol{R}^n$. To construct $\mathcal{B}$ we only need one local tree from each non-recombining block. Let this subset of trees and recombinations be represented by the vectors $\boldsymbol{T}$ and $\boldsymbol{R}$, respectively (we will drop the superscript for simplicity). For each local tree T_i and node $v_{i,j} \in V(T_i)$, we create a node $u_{i,j} \in V(\mathcal{B})$. Then we add a directed edge $(u_{i,j}, u_{i+1,k}) \in E(\mathcal{B})$ if, and only if, the branches above $v_{i,j}$ and $v_{i+1,k}$

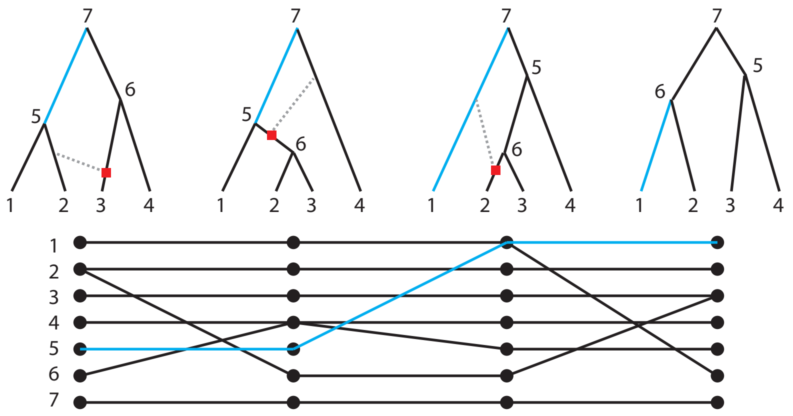

Supplementary Text Figure S1-3: **Use of branch graph to select a series of internal branches for removal.** The branch graph $\mathcal{B}$ (bottom) describes which branches in the local trees (top) share ancestry. Each node in the local trees has a corresponding node in the branch graph as indicated by the numbering scheme. Directed edges connect nodes in neighboring blocks if, and only if, the corresponding nodes in the local trees share ancestry. A path along the branch graph (blue at bottom) represents a valid series of branches (blue at top) in the local trees for removal and resampling.

share ancestry.

We define "shared ancestry" as follows. Let $z_{i+1} = (w_{i+1}, u_{i+1})$ represent the recombination point and $y_{i+1} = (x_{i+1}, t_{i+1})$ represent the recoalescing point leading from local tree T_i to local tree T_{i+1}. In addition, let M be a mapping such that $M(v_{i,j}) = v_{i+1,k}$ if $v_{i,j}$ and $v_{i+1,k}$ represent precisely the same coalescent event in trees T_i and T_{i+1}, respectively. Notice that the node above the recombination branch w_{i+1} does not map to any node in T_{i+1}, that is, $M(p(w_{i+1})) = \emptyset$. Also, the new node created in T_{i+1} by recoalescence, which we denote v^+_{i+1}, does not have any node mapping to it. However, all other nodes in T_i and T_{i+1} have a one-to-one mapping.

Shared ancestry can occur in three ways. Consider two arbitrary nodes in adjacent local trees, $v_{i,j}$ and $v_{i+1,k}$. First, if $M(v_{i,j}) \neq \emptyset$ and $v_{i+1,k} \neq v^+_{i+1}$, then $v_{i,j}$ and $v_{i+1,k}$ share ancestry if, and only if, $M(v_{i,j}) = v_{i+1,k}$. Second, if $M(v_{i,j}) = \emptyset$—meaning that $v_{i,j} = p(w_{i+1})$ is the node that is eliminated by the recombination between i and $i+1$—then $v_{i,j}$ and $v_{i+1,k}$ share ancestry if, and only if, $v_{i+1,k}$ is the *remaining child* of the eliminated node. By "remaining child" we mean that $v_{i+1,k} = M(\text{sibling}(w_{i+1}))$ if $\text{sibling}(w_i) \neq x_i$ or $v_{i+1,k} = p(M(\text{sibling}(w_{i+1})))$ otherwise. Finally, if $v_{i+1,k} = v^+_{i+1}$, then $v_{i,j}$ and $v_{i+1,k}$ share ancestry if, and only if, the recoalescence occurs on the branch above $v_{i,j}$, that is, $v_{i,j} = x_i$.

These rules produce a graph $\mathcal{B}$ such that, for each local block i, there is one node (the one above which the recoalescence occurs) with an out-degree of two, while all other nodes have an out-degree of one. Similarly, for each local block, there is one node with in-degree of two (the remaining child of the node eliminated by the recombination) and all other nodes have an in-degree of one. A directed path in the branch graph indicates a series of branches valid for removal.

The number of directed paths indicates the number of possible ways to remove internal branches according to this

scheme. This number can be computed in a straightforward way using dynamic programming. Let $P_{i,j}$ represent the number of paths ends in block i on branch j. This value can be computed recursively as follows:

$$P_{i,j} = \begin{cases} 1, & \text{if } i = 1 \\ \sum_k P_{i-1,k} I[(v_{i-1,k}, v_{i,j}) \in E(\mathcal{B})], & \text{otherwise.} \end{cases} \tag{S17}$$

Thus, the total number of directed paths can be computed as $\sum_j P_{m,j}$ where m is the index of the last local block. Paths can be sampled uniformly using a standard traceback procedure. Starting with the last block m, the last node j can be chosen with probability

$$\frac{P_{m,j}}{\sum_j P_{m,j}}. \tag{S18}$$

Given a chosen node $v_{i,j}$, the next node $v_{i-1,k}$ in the traceback can be chosen with probability,

$$\frac{P_{i-1,k}}{P_{i,j}}. \tag{S19}$$

Gibbs and Metropolis-Hastings Sampling of ARGs

Our goal is to sample ARGs G^n from the posterior distribution given the model parameters $\Theta = (\boldsymbol{N}, \mu, \rho)$ and the data D^n, namely

$$P(G^n \mid \Theta, D^n). \tag{S20}$$

Using our threading method, we can define both Gibbs and generalized Metropolis-Hastings Markov chain Monte Carlo (MCMC) methods for sampling ARGs. Let g and g' be two possible values for the random variable G^n. Let $q(g \to g')$ give the probability of proposing g' given g under some proposal procedure. The Metropolis-Hastings algorithm requires that the acceptance probability for the proposed move must be,

$$A(g \to g') = \min\left(1, \frac{P(G^n = g' \mid \Theta, D^n)}{P(G^n = g \mid \Theta, D^n)} \frac{q(g' \to g)}{q(g \to g')}\right). \tag{S21}$$

Let us now consider a particular type of probabilistic proposal procedure. Let S be a random variable representing a random subgraph of an ARG g and let $S(g)$ give a restricted set of subgraphs of g. Given a current ARG g, randomly choose a subgraph $S = s$ and then sample from the posterior a new ARG g' in which the subgraph s is held fixed (i.e., all changes occur outside of s). We can now write the proposal probability as,

$$\begin{aligned} q(g \to g') &= \sum_{s \in S(g)} P(S = s \mid G^n = g)\, P(G^n = g' \mid S = s, \Theta, D^n) \\ &= \sum_{s \in S(g,g')} P(S = s \mid G^n = g)\, P(G^n = g' \mid S = s, \Theta, D^n), \end{aligned} \tag{S22}$$

where we use the notation $S(g,g')$ to indicate $S(g) \cap S(g')$, thereby enforcing the constraint that the sampled subgraph s must belong to the restricted sets for both the original ARG g and the proposed ARG g'. This proposal probability can be further simplified as follows:

$$\begin{aligned}
q(g \to g') &= \sum_{s \in S(g,g')} P(S = s \mid G^n = g) \frac{P(G^n = g', S = s \mid \Theta, D^n)}{\sum_{h: s \in S(h)} P(G^n = h, S = s \mid \Theta, D^n)} \\
&= \sum_{s \in S(g,g')} P(S = s \mid G^n = g) \frac{P(G^n = g' \mid \Theta, D^n)}{\sum_{h: s \in S(h)} P(G^n = h \mid \Theta, D^n)} \\
&= P(G^n = g' \mid \Theta, D^n) \sum_{s \in S(g,g')} P(S = s \mid G^n = g) \left[\sum_{h: s \in S(h)} P(G^n = h \mid \Theta, D^n) \right]^{-1}
\end{aligned} \tag{S23}$$

where the simplification in the second line is possible because S is a subgraph of G^n.

If we choose subgraphs uniformly from the set $S(g)$, such that $P(S = s \mid G^n = g) = 1/|S(g)|$, we can then write the acceptance probability as

$$\begin{aligned}
&A(g \to g') \\
&= \min\left(1, \frac{P(G^n = g'|\Theta, D^n)}{P(G^n = g|\Theta, D^n)} \frac{P(G^n = g|\Theta, D^n) \sum_{s \in S(g,g')} P(S = s|G^n = g') \left[\sum_{h: s \in S(h)} P(G^n = h|\Theta, D^n)\right]^{-1}}{P(G^n = g'|\Theta, D^n) \sum_{s \in S(g,g')} P(S = s|G^n = g) \left[\sum_{h: s \in S(h)} P(G^n = h|\Theta, D^n)\right]^{-1}}\right) \\
&= \min\left(1, \frac{\sum_{s \in S(g,g')} |S(g')|^{-1} \left[\sum_{h: s \in S(h)} P(G^n = h|\Theta, D^n)\right]^{-1}}{\sum_{s \in S(g,g')} |S(g)|^{-1} \left[\sum_{h: s \in S(h)} P(G^n = h|\Theta, D^n)\right]^{-1}}\right) \\
&= \min\left(1, \frac{|S(g)| \sum_{s \in S(g,g')} \left[\sum_{h: s \in S(h)} P(G^n = h|\Theta, D^n)\right]^{-1}}{|S(g')| \sum_{s \in S(g,g')} \left[\sum_{h: s \in S(h)} P(G^n = h|\Theta, D^n)\right]^{-1}}\right) \\
&= \min\left(1, \frac{|S(g)|}{|S(g')|}\right).
\end{aligned} \tag{S24}$$

For cases where $|S(g)| = |S(g')|$ the acceptance probability is always 1 and the procedure is a valid a Gibbs sampler. This is true for the case when $S(g)$ is the set of subgraphs of g where one sequence is removed from the ARG. If there are n sequences then $|S(g)| = n$.

For resampling internal branches, $|S(g)|$ is not as trivial to calculate, but it can be calculated using dynamic programming (see previous section). However, $|S(g)|$ will not always be equal to $|S(g')|$ and therefore there is a chance of rejection.

The rationale for using a proposal procedure based on conditioning on the subgraph is that uses the data to drive the proposal. Without such a strategy, the acceptance probability would be driven by changes in likelihood which can vary wildly when resampling large ARGs.

In order to establish that the stationary distribution of this Markov chain equals the desired posterior distribution, we must show that the chain is irreducible, aperiodic, and positive recurrent. First, the chain is irreducible because, given any two ARGs g and g', it is possible to find a sequence of proposed moves that will transform g to g'. To see that this is true, consider a subgraph $S(g,L)$ of g that is defined by removing threads from g until only a set of leaves L

remains. First consider the base case of a single leaf, $L = \{l\}$. In this case, we trivially have $S(g,L) = S(g',L)$, because both subgraphs are simply trunk genealogies. Now let us add one leaf at a time to L. Each time we add a leaf l to the set L, we can ensure that $S(g,L) = S(g',L)$ by removing the thread for l from g and then re-threading l in such a way that $S(g,L) = S(g',L)$. In this way, we can obtain any g' from any g using the threading operation.

Next, to see that the chain is aperiodic and positive recurrent, note that self-transitions $g \to g$ have nonzero probability. In addition, every transition in the Markov chain is reversible. Specifically, for any transition $g \to g'$, we choose a subgraph s and then sample g' conditional on s. Notice that based on the design of our branch removal procedure, if g' was sampled conditioned on s, then s can be obtained by applying the branch removal procedure to g'. Since s is a subgraph of g, g can be sampled by the threading procedure conditioned on s. Thus, the reverse transition $g' \to g$ must have non-zero probability. Together, nonzero self transitions and reversible non-self transitions guarantee that the chain is aperiodic. The chain is positive recurrent because it has a finite state space and is irreducible.

Supplementary Data Analysis

Evaluation of Discretized Sequentially Markov Coalescent in Data Generation

Following McVean and Cardin [2], we compared data sets generated under the DSMC with ones generated under the sequentially Markov coalescent (SMC) and the coalescent-with-recombination (CwR). First, we simulated 100 kb regions of 20 sequences using our standard simulation parameters including four different μ/ρ ratios (see Methods). We carried out parallel simulations under the DSMC, the SMC, and the CwR, assuming various numbers of time intervals (K) for the DSMC. At all recombination rates, the DSMC, SMC, and CwR models produced very similar distributions of recombination counts (Supplementary Figure S1A). These distributions were essentially indistinguishable at lower recombination rates, and the DSMC exhibited only a slight excess of recombinations events at higher rates. Interestingly, the DSMC appeared not to be highly sensitive to the number of time intervals K, although the excess in recombination events at high rates was most pronounced under the most coarse-grained discretization scheme ($K = 9$).

In a second simulation experiment, we assumed a single ratio of $\mu/\rho = 2$ and considered four effective population sizes (N), ranging from 10,000 to 30,000 individuals. In this comparison, we used the number of segregating sites as the summary statistic of interest. As expected, this statistic increases approximately linearly with N under all models. Once again, we found that the CwR, SMC, and DSMC models produced nearly identical distributions of counts, with only a minor inflation under the coarsest discretization schemes (Supplementary Figure S1B). Overall, these comparisons indicate that the discretization scheme used by the DSMC has at most a minimal effect on measurable patterns of mutation and recombination at realistic parameter values for human populations, suggesting that the model will be adequate for use in inference.

Information in the ARG about Population Phylogenies

To explore the usefulness of *ARGweaver* in demographic analysis, we attempted to infer a population phylogeny with admixture edges for the 11 human populations represented in the Complete Genomics data set (see Supplementary Figure S20 for a list of populations). We began by extracting genealogies from two loci per ∼2-Mb block, such that each genealogy was approximately 1 Mb from the next one. We then computed a consensus tree at each locus using a standard majority rule for edges across the sampled local trees. Here we considered every 100th sample from our *ARGweaver* sampling run (21 trees per locus). In addition, we collapsed identical adjacent local consensus trees into a single tree. In the end, our analysis considered 2,304 trees from 1,376 ∼2-Mb blocks. Next, we reduced each tree to a single representative of each of the 11 represented populations by selecting one haploid sample per population at random, and extracting the subtree spanning these 11 leaves.

We then analyzed these 11-leaf trees with the PhyloNet program. PhyloNet finds a population tree that minimizes the number of "deep coalescences" required for reconciliation with a given set of local trees, allowing both for phylogenetic discordance from incomplete lineage sorting (see, e.g., [3]) and for a specified number of hybridization (admixture) events between groups [4, 5]. We ran PhyloNet version 3.5.0 on the 11-leaf consensus trees using the InferNetwork_parsimony option and specifying a maximum number of hybridization nodes in the range 0–5. Identical phylogenies and networks were obtained for four different random choices of haploid samples per population.

In the absence of hybridization (0 nodes), PhyloNet recovers the expected phylogeny for these populations, with the deepest divergence event between African and Eurasian populations, and successively more recent events separating the European from the Asian populations, the South Asian Indian population from the East Asian Han Chinese and Japanese, and the West African Yoruba from the East African Luhya and Maasai (Supplementary Figure S20A). The most recent events separate the relatively geographically and ethnically similar Han Chinese and Japanese populations, Luhya and Massai populations, and Tuscan and CEU (Utah residents of Northern and Western European ancestry) populations (Supplementary Figure S20A). The Mexican and Puerto Rican individuals cluster with the Europeans, and the African American individual clusters with the West African Yorubans, consistent with recent analyses of these admixed populations [6].

When admixture nodes are permitted, PhyloNet uses them to explain gene flow in several populations identified as admixed in other analyses [6, 7], including the Maasai (MKK), African Americans (ASW), Mexicans (MXL), and Puerto Ricans (PUR) (Supplementary Figure S20B). In most cases, the inferred source populations are consistent with previous studies, but there are two major anomalies in the inferred networks. The first anomaly is the use of the Gujarati Indians (GIH) as a source population for the admixed MXL and PUR populations. This may be a consequence of the absence in this data set of a better surrogate for Native American source populations for the MXL and PUR or it may reflect European admixture in India [7]. The second anomaly is the inference of admixture from the MXL and Tuscan (TSI) individuals in the CEU sample. Overall, it appears that the program correctly identifies a complex pattern of gene flow among the Latino, European, and African populations but is unable to reconstruct the precise topology of this subnetwork.

References

1. Paul JS, Steinrücken M, Song YS (2011) An accurate sequentially Markov conditional sampling distribution for the coalescent with recombination. Genetics 187: 1115–1128.

2. McVean GAT, Cardin NJ (2005) Approximating the coalescent with recombination. Philos Trans R Soc Lond B Biol Sci 360: 1387–1393.

3. Siepel A (2009) Phylogenomics of primates and their ancestral populations. Genome Res 19: 1929–1941.

4. Than C, Nakhleh L (2009) Species tree inference by minimizing deep coalescences. PLoS Comput Biol 5: e1000501.

5. Yu Y, Barnett RM, Nakhleh L (2013) Parsimonious inference of hybridization in the presence of incomplete lineage sorting. Syst Biol 62: 738–751.

6. Kidd JM, Gravel S, Byrnes J, Moreno-Estrada A, Musharoff S, et al. (2012) Population genetic inference from personal genome data: impact of ancestry and admixture on human genomic variation. Am J Hum Genet 91: 660–671.

7. The International HapMap 3 Consortium (2010) Integrating common and rare genetic variation in diverse human populations. Nature 467: 52–58.
